# Synthesis of 1-Substituted Bicyclo[2.1.1]hexan-2-ones via a Sequential SmI_2_-Mediated Pinacol Coupling and Acid-Catalyzed Pinacol Rearrangement Reaction

**DOI:** 10.1021/acs.orglett.4c03541

**Published:** 2024-10-22

**Authors:** Yung-Chi Lee, Yi-Chen Chen, Chun-Fu Wu, Woo-Jin Yoo

**Affiliations:** † Department of Chemistry, National Taiwan University, No. 1, Sec. 4, Roosevelt Road, Taipei 10617, Taiwan; ‡ Center for Emerging Materials and Advanced Devices, National Taiwan University, No. 1, Sec. 4, Roosevelt Road, Taipei 10617, Taiwan

## Abstract

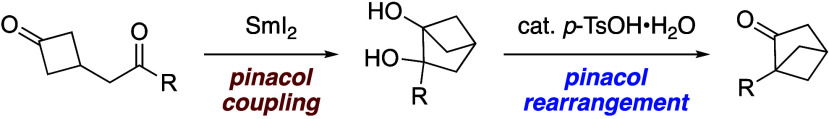

A two-step procedure, combining a SmI_2_-mediated transannular pinacol coupling reaction with an acid-catalyzed pinacol rearrangement process, was employed to prepare a diverse range of 1-substituted bicyclo[2.1.1]hexan-5-ones from cyclobutanedione derivatives. To underscore the significance of these bicyclic ketones in drug synthesis, an sp^3^-rich analog of nitazoxanide, a well-known antiparasitic and antiviral agent, was synthesized.

The concept of “escape from flatland” has become a point of emphasis in drug discovery programs as a means to improve the pharmacokinetic profiles of drug candidates, thereby increasing their probability of finding clinical success.^1^ To address the lack of 3-dimensionality in pharmaceuticals, the isosteric replacement of benzene rings, which are the most prominent ring system in small molecule drugs,^2^ with conformationally constrained polycyclic compounds has gained prominence in recent years.^3^ Among them, disubstituted bicyclo[2.1.1]hexanes (BCHs) have attracted considerable attention as promising candidates to act as isosteres for *ortho*- and *meta*-substituted benzenes (Scheme 1a).^3c^ Reflecting the growing importance of this class of nonplanar structures, several synthetic strategies based on intramolecular [2 + 2] cycloadditions of substituted 1,5-hexadienes,^4^ intermolecular [2 + 2] cycloadditions between bicyclobutanes with alkenes,^5^ intramolecular cyclization reactions,^6^ C–H functionalization of monosubstituted BCHs,^4f,7^ and molecular rearrangements^8^ have emerged as means to access disubstituted BCHs.

**Scheme 1:**
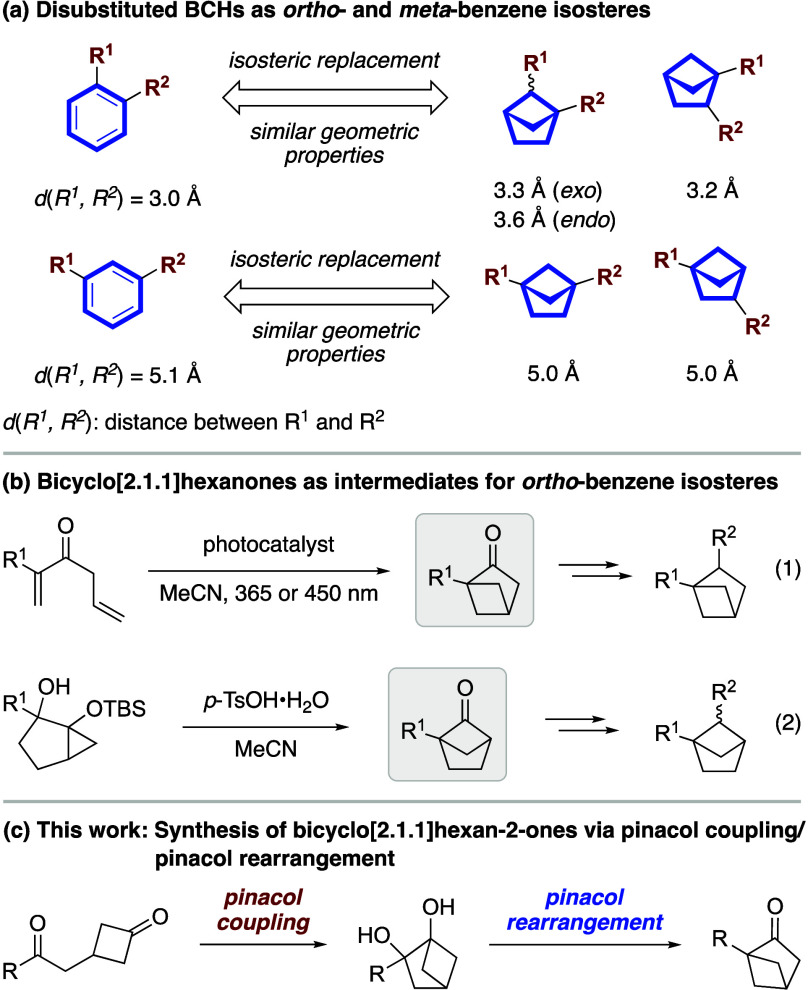
Disubstituted Bicyclo[2.1.1]hexanes as Potential *ortho*- and *meta*-Substituted Benzene Isosteres

Previously, it was shown that 1-substituted bicyclo[2.1.1]hexan-2-ones, derived from an intramolecular [2 + 2] cycloaddition reaction, could serve as a common intermediate to generate various 1,2-disubstituted BCHs (Scheme 1b, eq 1).^4e^ However, challenges related to the synthesis of 2-substituted hexa-1,5-dien-3-ones limited its substrate scope. On the other hand, we recently reported a *p*-TsOH-catalyzed pinacol rearrangement process to obtain 1-substituted bicyclo[2.1.1]hexan-5-ones, which functioned as a precursor to a wide range of 1,5-disubstituted BCHs (eq 2).^8e^ We envisioned that a similar strategy of employing the pinacol rearrangement reaction with 2-substituted bicyclo[2.1.1]hexane-1,2-diols could offer an alternative route to 1-substituted bicyclo[2.1.1]hexan-2-ones. Herein, we describe a sequential method that integrates intramolecular pinacol coupling with a pinacol rearrangement reaction to deliver a wide range of 1-substituted bicyclo[2.1.1]hexan-2-ones (Scheme 1c).

One of the most powerful methods to obtain vicinal diols is the pinacol coupling reaction,^9^ and we considered this approach to synthesize 2-substituted bicyclo[2.1.1]hexane-1,2-diols. Starting from commercially available 3-oxocyclobutane-1-carboxylic acid (**1**), aldehyde **2** was obtained through a previously reported 5-step procedure.^6c^ Next, **2** was then treated with Grignard reagents, followed by IBX-mediated oxidation and acetal deprotection using *p*-TsOH·H_2_O, to afford diones **3a**–**o** in moderate to good yields (Scheme 2).

**Scheme 2:**
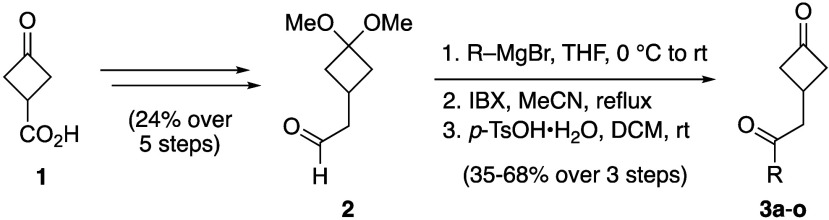
Synthetic Route Towards Diones **3a**–**o**

With a reliable method to access diones **3a**–**o** in hand, we began our optimization studies by examining the intramolecular pinacol coupling reaction using dione **3a** as a model substrate (Table 1). Although a wide range of reducing reagents can promote the pinacol coupling reaction, Kagan’s reagent (SmI_2_), a well-known single-electron reductant, is frequently employed in various reductive coupling processes, including the pinacol reaction.^10^ When it was applied to the pinacol reaction of **3a**, bicyclic diol **4a** was obtained in a moderate yield (entry 1). Since the behavior of SmI_2_ can be adjusted with various additives,^11^ several commonly used reagents were investigated (entries 2–4). Unfortunately, despite the high conversion of **3a**, vicinal diol **4a** was not detected, and other unproductive pathways, such as reduction to alcohols, were detected. In order to improve the conversion of **3a**, both the equivalents of SmI_2_ and the reaction time were increased, but no changes were observed (entries 5–6). Surprisingly, when the order of addition was reversed, with substrate **3a** being introduced to SmI_2_, full conversion was observed and the desired bicyclic diol **4a** was obtained in a good yield (entry 7).

**Table 1:**
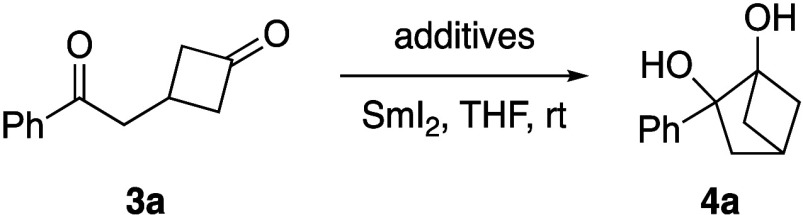
Optimization Studies for the SmI_2_-Mediated Pinacol Coupling of Diketone **3a**^a^

Entry	SmI_2_ (equiv)	Additive	Conv. (%)^b^	Yield (%)^b^
1	3.0	–	82	61
2	3.0	HMPA	>95	N.D.
3	3.0	MeOH	88	N.D.
4	3.0	HFIP, H_2_O	95	N.D.
5	4.0	–	78	58
6^c^	4.0	–	79	61
7^d^	4.0	–	>95	80

a Reaction conditions: SmI_2_ (0.10 M in THF) and additives (6.0 mmol) were added to a solution of dione **3a** (0.25 mmol) in THF (2.5 mL) at room temperature and stirred for 0.5 h.

b Conversion of **3a** and yield of **4a** were determined by ^1^H NMR analysis using triphenylmethane as an internal standard.

c Reaction time was increased to 2 h.

d The solution of **3a** was added to the solution of SmI_2_.

With an effective method established for the intramolecular pinacol reaction of dione **3a**, we began our investigations into the acid-catalyzed pinacol rearrangement of vicinal diol **4a**. However, due to the relative polarity of **4a**, we opted to directly use the crude bicyclic diol for the rearrangement step (Table 2). Taking inspiration from our previous work for the pinacol rearrangement of 1-silyloxybicyclo[3.1.0]hexan-2-ols,^8e^ we examined various protic and Lewis acids and found several viable catalysts (entries 1–4). In the end, we chose to proceed with *p*-TsOH·H_2_O as the catalyst due to its low cost and ease of use.

**Table 2:**
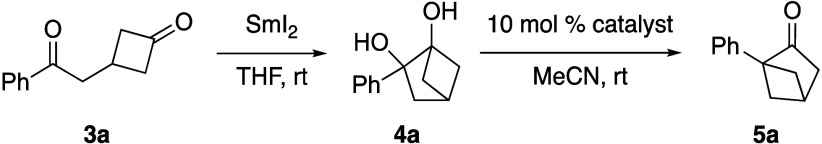
Optimization Studies for the Sequential SmI_2_-Mediated Pinacol Coupling and Acid-Catalyzed Pinacol Rearrangement of Diketone **3a**^a^

Entry	Catalyst	Yield (%)^b^
1	*p*-TsOH·H_2_O	68
2	TFA	N.D.
3	FeCl_3_	67
4	Cu(OTf)_2_	67

a Reaction conditions: step 1: dione **3a** (0.25 mmol) in THF (2.5 mL) was added to a solution of SmI_2_ (0.01 M in THF, 1.0 mmol) and stirred for 0.5 h; step 2: catalyst (0.025 mmol, 10 mol %) in MeCN (0.5 mL).

b Yield of **5a** was determined by ^1^H NMR analysis using triphenylmethane as an internal standard.

With the optimized conditions in hand, the substrate scope for the two-step procedure to convert diketones **3a**–**o** into 1-substituted bicyclic ketones **5a**–**o** was investigated (Table 3). Initial studies revealed that a minor modification was needed for the first step of the process. Specifically, increasing the reaction temperature for the pinacol coupling to 50 °C improved the overall yield of the two-step procedure.^12^ In general, diones **3b**-**g**, which bear electron-withdrawing moieties on the aryl substituent, were found to relatively poor substrates (entries 2–7). In contrast, aryl ketones **3h** and **3i** gave good yields of **5h** and **5i** (entries 8–9). These results can be attributed to the lower yields observed in the pinacol coupling reaction with substrates bearing electron-withdrawing groups, as well as the difficulty associated with generating a carbocationic intermediate during the pinacol rearrangement step. When diones **3l** and **3m** were examined for the two-step pinacol process, these heteroaromatic compounds did not produce the expected bicyclic ketones in any significant yields (entries 12–13). In the case of **3m**, the reduction of the pyridyl group was observed,^13^ as indicated by ^1^H NMR analysis of the crude reaction mixture. Finally, when aliphatic substituted diones **3n**–**o** were subjected to the optimized conditions, the anticipated bicyclic ketones **5n**–**o** were obtained in moderate yields (entries 14–15). To demonstrate the practical utility of our synthetic method, the sequential pinacol coupling and pinacol rearrangement of dione **3n** were carried out on a larger scale (Scheme 3). Subjecting **3n** to the optimized pinacol reaction conditions provided bicyclic diol **4n** in a moderate yield. Interestingly, **4n** was found to be relatively nonpolar and could be easily purified by column chromatography. Finally, treating **4n** with the optimized pinacol rearrangement conditions resulted in near-quantitative conversion to the desired bicyclic ketone **5n**.

**Scheme 3:**
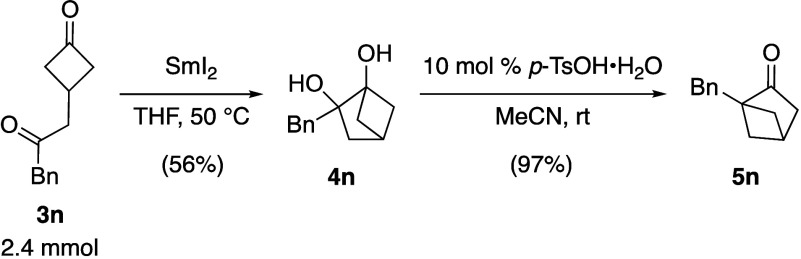
Large-Scale Sequential Pinacol Coupling/Pinacol Rearrangement of Dione **3n**

**Table 3:**
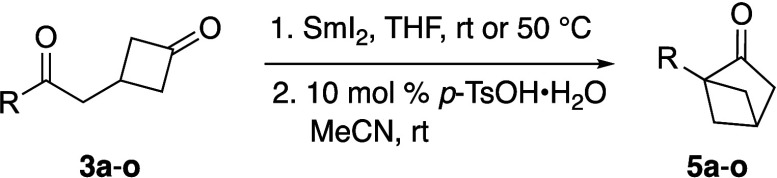
Substrate Scope of the Tandem Pinacol Coupling/Pinacol Rearrangement of Diones **3a**–**o**^a^

Entry	R	Yield (%)^b^
1^c^	Ph (**3a**)	63
2	4-Cl-C_6_H_4_ (**3b**)	40
3	4-F-C_6_H_4_ (**3c**)	65
4	3-F-C_6_H_4_ (**3d**)	57
5	2-F-C_6_H_4_ (**3e**)	40
6	4-CF_3_-C_6_H_4_ (**3f**)	12
7	4-CN-C_6_H_4_ (**3g**)	16
8	4-Me-C_6_H_4_ (**3h**)	80
9	4-MeO-C_6_H_4_ (**3i**)	82
10	3-MeO-C_6_H_4_ (**3j**)	62
11	2-MeO-C_6_H_4_ (**3k**)	56
12	2-Thiophenyl (**3l**)	>5^d^
13	3-Pyridyl (**3m**)	N.D.
14	Bn (**3n**)	42
15	*c*-Pentyl (**3o**)	45

a Reaction conditions: step 1: dione **3a**–**o** (0.50 mmol) in THF (5.0 mL) was added to a solution of SmI_2_ (0.01 M in THF, 2.0 mmol) heated at 50 °C and stirred for 0.5 h; step 2: *p*-TsOH·H_2_O (0.050 mmol, 10 mol %) in MeCN (1.0 mL).

b Yields of isolated products **5a**–**o** were based on **3a**–**o** over two steps.

c Step 1 was conducted at room temperature.

d Yield of **5l** was determined by ^1^H NMR analysis using triphenylmethane as an internal standard.

To highlight the potential of these bicyclic ketones as key building blocks in drug development, an sp^3^-rich analog of nitazoxanide, a widely used antiparasitic^14^ and antiviral^15^ compound for the treatment of diarrhea caused by protozoan parasites, was synthesized (Scheme 4). This synthesis began with the NaBH_4_-mediated reduction of **5a**, followed by acetylation of the resulting secondary alcohol to furnish the 1,2-disubstituted BCH **6** in an excellent yield. Next, RuCl_3_-catalyzed oxidative cleavage of the phenyl group,^16^ and the subsequent EDCI-mediated amide coupling reaction, afforded the saturated variant of nitazoxanide **7** with a 37% yield over two steps.

**Scheme 4:**
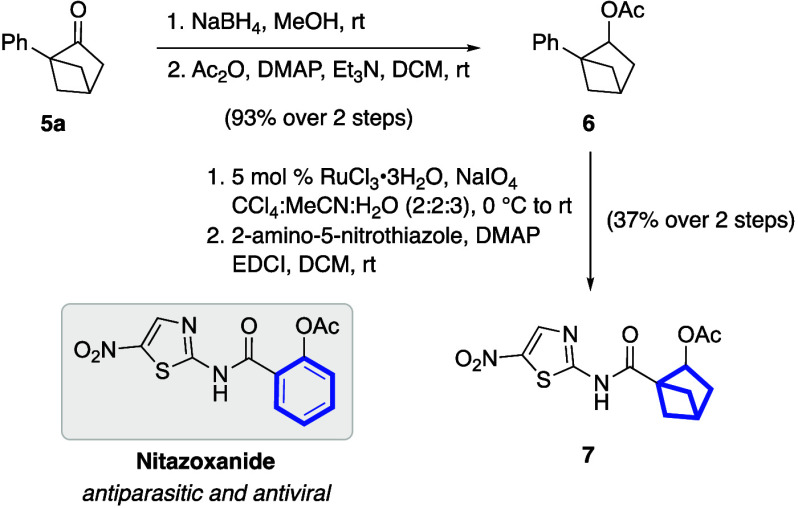
Synthesis of a Saturated Variant of Nitazoxanide

The probable mechanisms of the two key pinacol processes, which play crucial roles in the overall reaction, are depicted in Scheme 5. In the pinacol coupling reaction, the bidentate coordination of SmI_2_ to both ketone groups, accompanied by the inner-sphere single-electron reduction^17^ of the cyclobutanone,^18^ is expected to generate ketyl radical intermediate **8** (Scheme 5a). Next, there are two plausible reaction pathways **8** can undergo. In path a, intramolecular cyclization of the nucleophilic ketyl radical anion to the Lewis acid-activated ketone would produce alkoxy radical **9**, which could then engage in a second single-electron reduction to give rise to **10**.^19^ Finally, protonation of **10** results in the formation of vicinal diol **4**. Alternatively, ketyl anion **8** could go through a second single-electron reduction to form Streitwieser dimer **11**, which, upon dissociation of SmI_3_ and radical recombination, results in the generation of **10** (path b).^20^ In the pinacol rearrangement step, protonation of bicyclic diol **2** should selectively generate 3° carbocation **12**,^21^ which can then undergo a 1,2-alkyl shift to produce bicyclic ketone **3** (Scheme 5b).

**Scheme 5:**
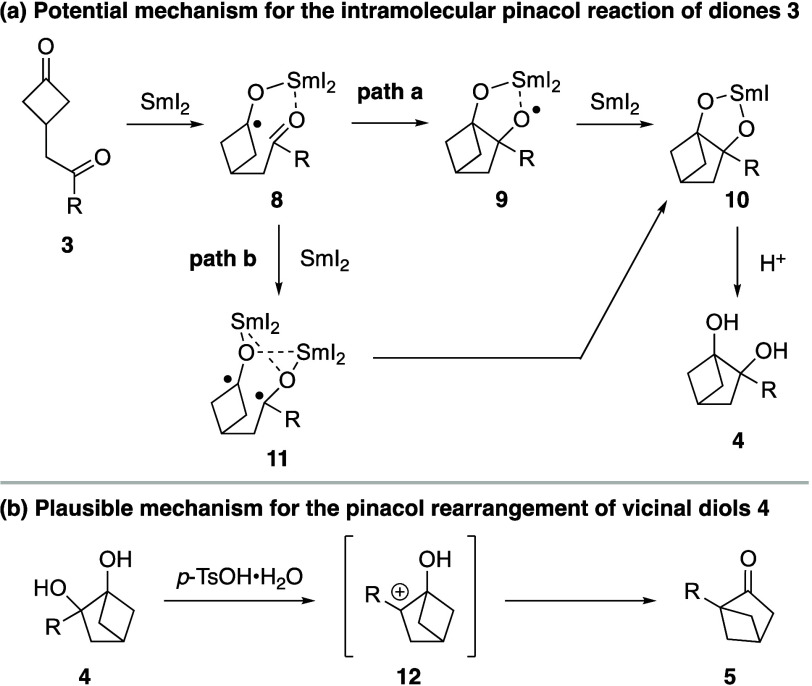
Proposed Mechanisms

In conclusion, a SmI_2_-mediated transannular pinacol reaction, followed by a *p*-TsOH-catalyzed pinacol rearrangement of the resulting bicyclic vicinal diol, led to the formation of a diverse range of 1-substituted bicyclo[2.1.1]hexan-2-ones. Additionally, the conversion of **5a** into a saturated analog of a marketed pharmaceutical demonstrates that these bicyclic ketones are promising intermediates for the synthesis of 3D-rich and molecularly complex compounds.

## Data Availability

The data underlying this study are available in the published article and its Supporting Information.

## Supporting Information Available

Additional information, experimental procedures, characterization of new compounds, spectra data (PDF)
