# Supplementary material for: Synthesis of 1-Substituted Bicyclo[2.1.1]hexan-2-ones via a Sequential SmI2-Mediated Pinacol Coupling and Acid-Catalyzed Pinacol Rearrangement Reaction
Source: Org Lett. 2024 Oct 22;26(43):9352–6. doi: 10.1021/acs.orglett.4c03541 (PMC11536404; doi:10.1021/acs.orglett.4c03541)
Supplement: Supplementary file 1 — ol4c03541_si_001.pdf [file ol4c03541_si_001.pdf]

# Supporting Information

***Synthesis of 1-Substituted Bicyclo[2.1.1]hexan-2-ones via a Sequential $SmI_2$-Mediated Pinacol Coupling and Acid-Catalyzed Pinacol Rearrangement Reaction***

*Yung-Chi Lee,[a] Yi-Chen Chen[a] Chun-Fu Wu,[a] and Woo-Jin Yoo[a,b]*

[a] Department of Chemistry, National Taiwan University, No. 1, Sec. 4, Roosevelt Road, Taipei 10617, Taiwan

[b] Center for Emerging Materials and Advanced Devices, National Taiwan University, No. 1, Sec. 4, Roosevelt Road, Taipei 10617, Taiwan

## Table of Contents

Part I: Preparation of Cyclobutanones **3a-o**..........S-2

Part II: Preliminary Results for the Tandem Pinacol Coupling/Pinacol Rearrangement of Diones..........S-8

Part III: Substrate Scope for the Synthesis of **5a-o** via the Tandem Pinacol Coupling/Pinacol Rearrangement of Diones **3a-o**..........S-9

Part IV: Larger Scale Synthesis of Bicyclic Ketone **5n**..........S-13

Part V: Synthesis of Saturated Analog of Nitazoxanide..........S-14

Part VI: References..........S-15

Part VII: Copies of NMR Spectra Data..........S-16

**General Information:** $^{1}H$, $^{13}C$, and $^{19}F$ NMR spectra were recorded on Bruker AVIII HD 400 spectrometer in $CDCl_3$. Chemical shifts were reported in parts per million (ppm) from chloroform using the solvent resonance as the internal standard ($CHCl_3$: δ 7.26 ppm) or tetramethylsilane (δ 0.00 ppm) for $^{1}H$ NMR and ($CDCl_3$: δ 77.00 ppm) for $^{13}C$ NMR. For $^{19}F$ NMR, trifluoroacetic acid was used as an external standard (δ –76.55 ppm). IR spectra were measured on a Thermo Scientific Nicolet iS5 FT-IR spectrometer. High-resolution mass spectroscopic data were collected at the Instrumentation Center of National Taiwan University using a Bruker microTOF-QII, National Taiwan Normal University using a Waters Xevo G2-S Tof, National Tsing Hua University using a JEOL JMS-T100LP 4G, and National Chung Hsing University using a Thermo Scientific Orbitrap LTQ XL. Column chromatography was carried out using either silica gel (Silica gel 60, 40-63 μm) or aluminium oxide (Aluminium oxide 90 active neutral, 63-200 μm) from Merck.

**Reagents:** Unless stated otherwise, commercial reagents (Energy Chemical, Nova Materials, Sigma-Aldrich, Thermo Fisher Scientific, Merck Millipore, Shimakyu Chemical, TCI, Tedia, Encore Chemicals, ECHO Chemical, Duksan Pure Chemicals, and Matrix Scientific) were used without purification. Solvents were either purified by distillation under dry nitrogen from $CaH_2$ or obtained from a solvent purification system (Vigor).

Part I: Preparation of Cyclobutanones **3a-o**

Cyclobutanones **3a**-**m** were prepared according to literature reports.[1]

General procedure for the synthesis of **S1n**-**o** *via* Grignard addition reaction

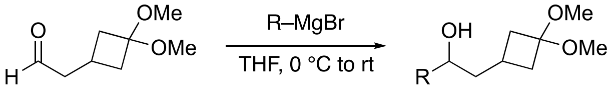


**2** **S1n-o**

In a flame-dried round bottom flask under a balloon of dry nitrogen was added **2** (316.4 mg, 2.000 mmol) and THF (4.0 mL). After the reaction vessel was cooled to 0 °C with an ice bath, a solution of the Grignard reagent (3.0 mL, 1.0 M in THF, 3.0 mmol) was added dropwise. After the addition of the organometallic reagent was finished, the reaction mixture was allowed to warm to room temperature and stirred for 1 hour. After the completion of the reaction (determined by TLC), the reaction mixture was cooled to 0 °C and then a saturated $NH_4Cl$ solution was added. The resulting biphasic mixture was extracted with DCM and the combined organic fractions were washed with brine, dried over $Na_2SO_4$, and concentrated under reduced pressure. The crude product was purified by flash column chromatography (silica gel) using EtOAc:hexane (20:80) as the eluent.

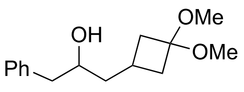
S1n

**1-(3,3-Dimethoxycyclobutyl)-3-phenylpropan-2-ol (S1n)** Following the above procedure with **2** (316.4 mg, 2.000 mmol) and benzylmagnesium chloride (3.0 mL, 1.0 M in THF, 3.0 mmol). The crude product was purified by flash column chromatography to afford **S1n** as a colorless oil (234.6 mg, 47%). $R_f$ = 1.2/4.0 (20% EtOAc in hexane); $^1$H NMR (400 MHz, $CDCl_3$) δ 7.36-7.29 (m, 2H), 7.24-7.18 (m, 3H), 3.80-3.75 (m, 1H), 3.14 (s, 3H), 3.11 (s, 3H), 2.81-2.77 (m, 1H), 2.66-2.60 (m, 1H), 2.39-2.31 (m, 2H), 2.30-2.21 (m, 1H), 1.80-1.60 (m, 5H); $^{13}$C NMR ($CDCl_3$, 101 MHz) δ 138.4, 129.3, 128.5, 126.4, 100.7, 71.4, 48.4, 48.2, 44.2, 43.5, 37.8, 37.4, 22.1; IR (neat, ZnSe, $cm^{-1}$) 3455, 2981, 2936, 2829, 1601, 1496, 1454, 1273, 1162, 1039, 852, 746, 701; HRMS (ESI/Q-TOF) m/z: $[M + Na]^+$ calcd for $C_{15}H_{22}NaO_3$ 273.1467; found 273.1469.

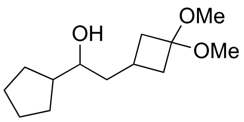
S1o

**1-Cyclopentyl-2-(3,3-dimethoxycyclobutyl)ethan-1-ol (S1o).** Following the above procedure with **2** (316.4 mg, 2.000 mmol) and cyclopentylmagnesium bromide (3.0 mL, 1.0 M in THF, 3.0 mmol). The crude product was purified by flash column chromatography to afford **S1o** as a colorless oil (296.8 mg, 65%). $R_f$ = 0.5/4.0 (20% EtOAc in hexane); $^1$H NMR (400 MHz, $CDCl_3$) δ 3.33-3.28 (m, 1H), 3.10 (s, 3H), 3.08 (s, 3H), 2.34-2.26 (m, 2H), 2.24-2.14 (m, 1H), 1.83-1.43 (m, 12H), 1.33-1.24 (m, 1H), 1.19-1.10 (m, 1H); $^{13}$C NMR ($CDCl_3$, 101 MHz) δ 100.7, 74.4, 48.4, 48.1, 16.4, 42.9, 37.8, 37.3, 29.1, 28.3, 25.6, 25.5, 22.1; IR (neat, ZnSe, $cm^{-1}$) 3423, 2947, 2687, 2829, 1450, 1272, 1221, 1189, 1161, 1134, 1072, 1042, 858; HRMS (ESI/Q-TOF) m/z: $[M + H]^+$ calcd for $C_{13}H_{24}NaO_3$ 251.1618; found 251.1615.

General procedure for the synthesis of **3a-o** *via* IBX oxidation and *p*-TsOH-mediated acetal deprotection

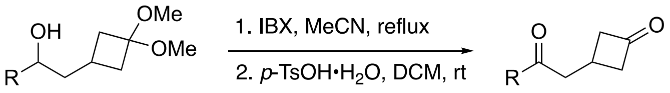

S1a-o 3a-o

In a round-bottom flask, **S1a-o** (3.0 mmol) was dissolved in acetonitrile (12 mL) and IBX (1.2601 g, 4.5000 mmol) was added. After stirring for 1 h under reflux, the mixture was filtered by celite and the filter cake was rinsed with EtOAc. Then the filtrate was concentrated under reduced pressure. Without further purification, the crude product was dissolved in DCM (6.0 mL) and then *p*-TsOH•$H_2O$ (2.8530 g, 14.998 mmol) was added. After the reaction mixture was stirred for 0.5 h at room temperature, water was added and the resulting biphasic mixture was extracted with EtOAc. The combined organic fractions were washed with brine, dried over $MgSO_4$, and then concentrated under reduced pressure. The crude product was purified by column chromatography (silica gel) using EtOAc:hexane (10:90) as the eluent.

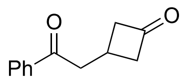

3a

**3-(2-Oxo-2-phenylethyl)cyclobutan-1-one (3a).** Following the above procedure with **S1a** (708.9 mg, 3.000 mmol), IBX (1.2601 g, 4.5000 mmol), and *p*-TsOH•$H_2O$ (2.8530 g, 14.998 mmol). The crude product was purified by flash column chromatography to afford **3a** as a pale yellow solid (435.2 mg, 77%). $R_f$ = 2.0/4.0 (25% EtOAc in hexane); $^1$H NMR (400 MHz, $CDCl_3$) δ 7.98-7.95 (m, 2H), 7.61-7.56 (m, 1H), 7.51-7.46 (m, 2H), 3.40-3.31 (m, 4H), 3.02-2.91 (m, 1H), 2.85-2.77 (m, 2H); $^{13}$C NMR ($CDCl_3$, 101 MHz) δ 207.4, 198.3. 136.4, 133.2, 128.5, 127.8, 52.6, 44.1, 19.6; IR (neat, ZnSe, $cm^{-1}$) 2915, 1776, 1735, 1684, 1654, 1597, 1578, 1448, 1406, 1369, 1322, 1227, 1203, 1180, 1098, 1001, 753; HRMS (ESI/Q-TOF) m/z: $[M + Na]^+$ calcd for $C_{12}H_{12}NaO_2$ 211.0730; found 211.0720.

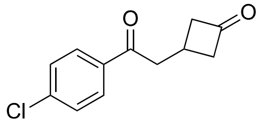

3b

**3-(2-(4-Chlorophenyl)-2-oxoethyl)cyclobutan-1-one (3b).** Following the above procedure with **S1b** (812.3 mg, 3.000 mmol), IBX (1.2601 g, 4.5000 mmol), and *p*-TsOH•$H_2O$ (2.8530 g, 14.998 mmol). The crude product was purified by flash column chromatography to afford **3b** as a pale yellow solid (509.4 mg, 76%). $R_f$ = 2.1/4.0 (25% EtOAc in hexane); $^1$H NMR (400 MHz, $CDCl_3$) δ 7.90 (d, *J* = 8.45 Hz, 2H), 7.45 (d, *J* = 8.45 Hz, 2H), 3.39-3.29 (m, 4H), 3.01-2.90 (m, 1H), 2.84-2.76 (m, 2H); $^{13}$C NMR ($CDCl_3$, 101 MHz) δ 207.3, 197.1, 139.7, 134.8, 129.3, 128.9, 52.7, 44.2, 19.6; IR (neat, ZnSe, $cm^{-1}$) 3089, 2984, 2918, 2894, 2568, 2434, 2354, 2289, 1788, 1678, 1586, 1489, 1409, 1385, 1211, 1092, 992, 822, 780; HRMS (ESI/Q-TOF) m/z: $[M + Na]^+$ calcd for $C_{12}H_{11}ClNaO_2$ 245.0340; found 245.0333.

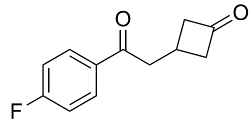

3c

**3-(2-(4-Fluorophenyl)-2-oxoethyl)cyclobutan-1-one (3c).** Following the above procedure with **S1c** (762.9 mg, 3.000 mmol), IBX (1.2601 g, 4.5000 mmol), and *p*-TsOH•$H_2O$ (2.8530 g, 14.998 mmol). The crude product was purified by flash column chromatography to afford **3c** as a pale yellow solid (552.7 mg, 90%). $R_f$ = 1.8/4.0 (25% EtOAc in hexane); $^1$H NMR (400 MHz, $CDCl_3$) δ 8.00 (d, *J* = 5.38 Hz, 1H), 7,98 (d, *J* = 5.38 Hz, 1H), 7.15 (t, *J* = 8.55 Hz, 2H), 3.40-3.29 (m, 4H), 3.01-2.90 (m, 1H), 2.84-2.76 (m, 2H); $^{13}$C NMR ($CDCl_3$, 101 MHz) δ 207.4, 196.7, 166.0 (d, *J* = 256.36 Hz), 133.0 (d, *J* = 2.5 Hz), 130.5 (d, *J* = 9.4 Hz), 115.8 (d, *J* = 22.4 Hz), 52.8, 44.2, 19.7; $^{19}$F NMR ($CDCl_3$, 376 MHz) δ -103.9; IR (neat, ZnSe, $cm^{-1}$) 2923, 1778, 1682, 1596, 1506, 1409, 1370, 1319, 1227, 1157, 1096, 994, 835; HRMS (ESI/Q-TOF) m/z: $[M + Na]^+$ calcd for $C_{12}H_{11}FNaO_2$ 229.0635; found 229.0637.

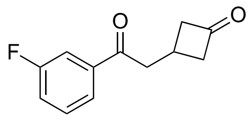

3d

**3-(2-(3-Fluorophenyl)-2-oxoethyl)cyclobutan-1-one (3d).** Following the above procedure with **S1d** (762.9 mg, 3.000 mmol), IBX (1.2601 g, 4.5000 mmol), and *p*-TsOH•$H_2O$ (2.8530 g, 14.998 mmol). The crude product was purified by flash column chromatography to afford **3d** as a pale yellow solid (573.4 mg, 93%). $R_f$ = 1.9/4.0 (25% EtOAc in hexane); $^1$H NMR (400 MHz, $CDCl_3$) δ 7.69 (dd, *J* = 7.83 Hz, *J* = 0.88 Hz, 1H), 7.59-7.56 (m, 1H), 7.43-7.38 (m, 1H), 7.24-7.19 (m, 1H), 3.31-3.24 (m, 4H), 2.95-2.83 (m, 1H), 2.78-2.72 (m, 2H); $^{13}$C NMR ($CDCl_3$, 101 MHz) δ 207.2, 197.0, 162.7

(d, $J$ = 245.1 Hz), 138.5 (d, $J$ = 6.1 Hz), 130.3 (d, $J$ = 7.5 Hz), 123.6 (d, $J$ = 2.5 Hz), 120.2 (d, $J$ = 21.6 Hz), 114.5 (d, $J$ = 22.9 Hz), 52.6, 44.3, 19.5; $^{19}$F NMR ($CDCl_3$, 376 MHz) δ -112.2; IR (neat, ZnSe, $cm^{-1}$) 2921, 1778, 1680, 1597, 1506, 1409, 1372, 1320, 1230, 1155, 1096, 994, 835; HRMS (EI) m/z: $[M]^+$ calcd for $C_{12}H_{11}FO_2$ 206.0743; found 206.0748.

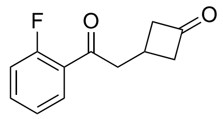


**3e**

**3-(2-(2-Fluorophenyl)-2-oxoethyl)cyclobutan-1-one (3e).** Following the above procedure with **S1e** (762.9 mg, 3.000 mmol), IBX (1.2601 g, 4.5000 mmol), and $p$-TsOH•$H_2O$ (2.8530 g, 14.998 mmol). The crude product was purified by flash column chromatography to afford **3e** as a pale yellow liquid (445.8 mg, 72%). $R_f$ = 2.0/4.0 (25% EtOAc in hexane); $^1$H NMR (400 MHz, $CDCl_3$) δ 7.90-7.86 (m, 1H), 7.56-7.51 (m, 1H), 7.25-7.22 (m, 1H), 7.17-7.12 (m, 1H), 3.36-3.29 (m, 4H), 2.98-2.89 (m, 1H), 2.83-2.76 (m, 2H); $^{13}$C NMR ($CDCl_3$, 101 MHz) δ 207.4, 196.4, 161.8 (d, $J$ = 252.2 Hz), 134.6 (d, $J$ = 8.9 Hz), 130.2, 124.9 (d, $J$ = 12.7 Hz), 124.3 (d, $J$ = 1.9 Hz), 116.5 (d, $J$ = 23.7 Hz), 49.1, 49.0, 19.3; $^{19}$F NMR ($CDCl_3$, 376 MHz) δ -109.2; IR (neat, ZnSe, $cm^{-1}$) 1782, 1684, 1609, 1576, 1480, 1452, 1372, 1321, 1270, 1210, 1195, 1153, 1101, 995, 837, 766; HRMS (ESI/Q-TOF) m/z: $[M + H]^+$ calcd for $C_{12}H_{12}FO_2$ 207.0821; found 207.0821.

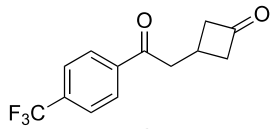


**3f**

**3-(2-Oxo-2-(4-(trifluoromethyl)phenyl)ethyl)cyclobutan-1-one (3f).** Following the above procedure with **S1f** (912.9 mg, 3.000 mmol), IBX (1.2601 g, 4.5000 mmol), and $p$-TsOH•$H_2O$ (2.8530 g, 14.998 mmol). The crude product was purified by flash column chromatography to afford **3f** as a pale yellow solid (554.5 mg, 72%). $R_f$ = 2.2/4.0 (25% EtOAc in hexane); $^1$H NMR (400 MHz, $CDCl_3$) δ 8.07 (d, $J$ = 8.26 Hz, 2H), 7.75 (d, $J$ = 8.26 Hz, 2H), 3.41-3.33 (m, 4H), 3.03-2.92 (m, 1H), 2.86-2.77 (m, 2H); $^{13}$C NMR ($CDCl_3$, 101 MHz) δ 207.1, 197.4, 139.1, 134.7 (q, $J$ = 32.9 Hz), 128.3, 125.8 (q, $J$ = 3.8 Hz), 123.4 (q, $J$ = 274.2 Hz), 52.9, 44.6, 19.6; $^{19}$F NMR ($CDCl_3$, 376 MHz) δ -64.2; IR (neat, ZnSe, $cm^{-1}$) 2282, 1781, 1690, 1409, 1370, 1324, 1203, 1126, 1167, 1109, 1065, 1016, 995, 829, 753; HRMS (ESI/Q-TOF) m/z: $[M + Na]^+$ calcd for $C_{13}H_{11}F_3NaO_2$ 279.0609; found 279.0608.

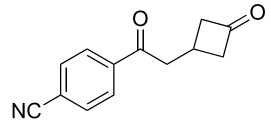


**3g**

**4-(2-(3-Oxocyclobutyl)acetyl)benzonitrile (3g).** Following the above procedure with **S1g** (784.0 mg, 3.000 mmol), IBX (1.2601 g, 4.5000 mmol), and $p$-TsOH•$H_2O$ (2.853 g, 14.998 mmol). The crude product was purified by flash column chromatography to afford **3g** as a pale yellow solid (510.2 mg, 80%). $R_f$ = 2.0/4.0 (25% EtOAc in hexane); $^1$H NMR (400 MHz, $CDCl_3$) δ 7.29 (d, $J$ = 8.61 Hz, 2H), 7.02 (d, $J$ = 8.61 Hz, 2H), 2.63-2.54 (m, 4H), 2.25-2.14 (m, 1H), 2.09-2.00 (m, 2H); $^{13}$C NMR ($CDCl_3$, 101 MHz) δ 206.9, 197.0, 139.2, 132.4, 128.2, 117.7, 116.4, 52.6, 44.4, 19.3; IR (neat, ZnSe, $cm^{-1}$) 2945, 2895, 2229, 1777, 1692, 1405, 1370, 1322, 1293, 1229, 1204, 1176, 1100, 998, 828; HRMS (ESI/Q-TOF) m/z: $[M + H]^+$ calcd for $C_{13}H_{12}NO_2$ 214.0868; found 214.0865.

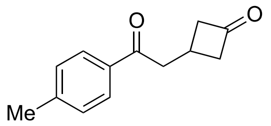

3h

**3-(2-Oxo-2-(*p*-tolyl)ethyl)cyclobutan-1-one (3h).** Following the above procedure with **S1h** (751.0 mg, 3.000 mmol), IBX (1.2601 g, 4.500 mmol), and *p*-TsOH•$H_2O$ (2.8530 g, 14.998 mmol). The crude product was purified by flash column chromatography to afford **3h** as a pale yellow solid (531.3 mg, 88%). $R_f$ = 1.9/4.0 (25% EtOAc in hexane); $^1$H NMR (400 MHz, $CDCl_3$) δ 7.86 (d, *J* = 8.16 Hz, 2H), 7.27 (d, *J* = 8.16 Hz, 2H), 3.38-3.29 (m, 4H), 3.01-2.89 (m, 1H), 2.84-2.76 (m, 2H), 2.42 (s, 3H); $^{13}$C NMR ($CDCl_3$, 101 MHz) δ 207.7, 198.0, 144.2, 134.1, 129.4, 128.1, 52.9, 44.2, 21.6, 19.8; IR (neat, ZnSe, cm$^{-1}$) 2957, 2876, 1781, 1679, 1605, 1407, 1370, 1320, 1229, 1203, 1181, 1097, 1002, 809; HRMS (ESI/Q-TOF) m/z: [M + Na]$^+$ calcd for $C_{13}H_{14}NaO_2$ 225.0892; found 225.0894.

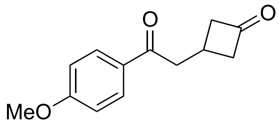

3i

**3-(2-(4-Methoxyphenyl)-2-oxoethyl)cyclobutan-1-one (3i).** Following the above procedure with **S1i** (799.0 mg, 3.000 mmol), IBX (1.2601 g, 4.5000 mmol), and *p*-TsOH•$H_2O$ (2.8530 g, 14.998 mmol). The crude product was purified by flash column chromatography to afford **3i** as a pale yellow solid (565.5 mg, 86%). $R_f$ = 1.8/4.0 (25% EtOAc in hexane); $^1$H NMR (400 MHz, $CDCl_3$) δ 7.90 (d, *J* = 8.75 Hz, 2H), 6.90 (d, *J* = 8.75 Hz, 2H), 3.83 (s, 3H), 3.32-3.22 (m, 4H), 2.95-2.84 (m, 1H), 2.80-2.72 (m, 2H); $^{13}$C NMR ($CDCl_3$, 101 MHz) δ 207.7, 196.8, 163.6, 130.1, 129.6, 113.7, 55.4, 52.7, 43.8, 19.8; IR (neat, ZnSe, cm$^{-1}$) 2973, 2841, 1778, 1668, 1600, 1575, 1509, 1458, 1419, 1369, 1314, 1232, 1211, 1202, 1172, 1098, 1026, 991, 833; HRMS (ESI/Q-TOF) m/z: [M + Na]$^+$ calcd for $C_{13}H_{14}NaO_3$ 241.0835; found 241.0834.

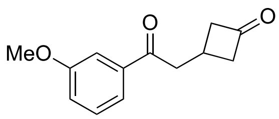

3j

**3-(2-(3-Methoxyphenyl)-2-oxoethyl)cyclobutan-1-one (3j).** Following the above procedure with **S1j** (799.0 mg, 3.000 mmol), IBX (1.2601 g, 4.5000 mmol), and *p*-TsOH•$H_2O$ (2.8530 g, 14.998 mmol). The crude product was purified by flash column chromatography to afford **3j** as a pale yellow solid (408.2 mg, 62%). $R_f$ = 1.9/4.0 (25% EtOAc in hexane); $^1$H NMR (400 MHz, $CDCl_3$) δ 7.53 (d, *J* = 7.53 Hz, 1H), 7.47 (s, 1H), 7.37 (t, *J* = 7.94 Hz, 1H), 7.11 (dd, *J* = 8.35 Hz, *J* = 2.56 Hz, 1H), 3.84 (s, 3H), 3.36-3.29 (m, 4H), 2.99-2.88 (m, 1H), 2.83-2.75 (m, 2H); $^{13}$C NMR ($CDCl_3$, 101 MHz) δ 207.4, 198.1, 159.7, 137.8, 129.5, 120.4, 119.5, 112.1, 55.2, 52.6, 44.2, 19.6; IR (neat, ZnSe, cm$^{-1}$) 2921, 2782, 1781, 1683, 1597, 1583, 1486, 1451, 1430, 1363, 1321, 1290, 1261, 1193, 1170, 1099, 1046, 786; HRMS (ESI/Q-TOF) m/z: [M + H]$^+$ calcd for $C_{13}H_{15}O_3$ 219.1021; found 219.1022.

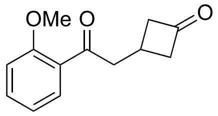

3k

**3-(2-(2-Methoxyphenyl)-2-oxoethyl)cyclobutan-1-one (3k).** Following the above procedure with **S1k** (799.0 mg, 3.000 mmol), IBX (1.2601 g, 4.5000 mmol), and *p*-TsOH•$H_2O$ (2.8530 g, 14.998 mmol). The crude product was purified by flash column chromatography to afford **3k** as a pale yellow liquid (557.3 mg, 85%). $R_f$ = 2.0/4.0 (25% EtOAc in hexane); $^1$H NMR (400 MHz, $CDCl_3$) δ 7.71-7.69 (m, 1H), 7.49-7.44 (m, 1H), 7.02-6.96 (m, 2H), 3.90 (s, 3H), 3.33-3.24 (m, 4H), 2.96-2.85 (m, 1H),

2.80-2.74 (m, 2H); $^{13}$C NMR ($CDCl_3$, 101 MHz) δ 208.3, 200.5, 158.7, 133.8, 130.2, 127.7, 120.7, 111.6, 55.4, 52.8, 49.6, 19.8; IR (neat, ZnSe, $cm^{-1}$) 2971, 2945, 2840, 1778, 1667, 1598, 1578, 1485, 1464, 1436, 1371, 1303, 1246, 1209, 1190, 1163, 1107, 1054, 1022, 997, 759; HRMS (ESI/Q-TOF) m/z: $[M + Na]^+$ calcd for $C_{13}H_{14}NaO_3$ 241.0841; found 241.0840.

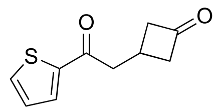


**3l**

**3-(2-Oxo-2-(thiophen-2-yl)ethyl)cyclobutan-1-one (3l).** Following the above procedure with **S1l** (727.0 mg, 3.000 mmol), IBX (1.2601 g, 4.5000 mmol), and *p*-TsOH•$H_2O$ (2.8530 g, 14.998 mmol). The crude product was purified by flash column chromatography to afford **3l** as a pale yellow solid (549.1 mg, 94%). $R_f$ = 2.0/4.0 (25% EtOAc in hexane); $^1$H NMR (400 MHz, $CDCl_3$) δ 7.71 (dd, *J* = 3.77 Hz, *J* = 1.07 Hz, 1H), 7.64 (dd, *J* = 4.89 Hz, *J* = 3.76 Hz, 1H), 7.12 (dd, *J* = 5.06 Hz, *J* = 3.72 Hz, 1H), 3.33-3.22 (m, 4H), 2.98-2.87 (m, 1H), 2.85-2.77 (m, 2H); $^{13}$C NMR ($CDCl_3$, 101 MHz) δ 207.2, 191.2, 143.8, 133.9, 131.9, 128.1, 52.8, 44.7, 19.9; IR (neat, ZnSe, $cm^{-1}$) 3089, 2921, 1778, 1658, 1518, 1415, 1373, 1309, 1235, 1210, 1098, 1057, 946, 857, 732; HRMS (ESI/Q-TOF) m/z: $[M + Na]^+$ calcd for $C_{10}H_{10}NaO_2S$ 217.0294; found 217.0290.

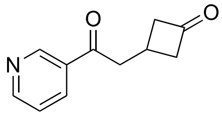


**3m**

**3-(2-Oxo-2-(pyridin-3-yl)ethyl)cyclobutan-1-one (3m).** Following the above procedure with **S1m** (711.9 mg, 3.000 mmol), IBX (1.2601 g, 4.5000 mmol), and *p*-TsOH•$H_2O$ (2.8530 g, 14.998 mmol). The crude product was purified by flash column chromatography to afford **3m** as a pale yellow solid (362.9 mg, 64%). $R_f$ = 2.0/4.0 (25% EtOAc in hexane); $^1$H NMR (400 MHz, $CDCl_3$) δ 9.13 (s, 1H), 8.75 (dd, *J* = 4.89 Hz, *J* = 0.98 Hz, 1H), 8.19 (dt, *J* = 8.12 Hz, *J* = 1.82 Hz, 1H), 7.40 (dd, *J* = 8.64 Hz, *J* = 4.85 Hz, 1H), 3.34-3.27 (m, 4H), 2.98-2.87 (m, 1H), 2.82-2.74 (m, 2H); $^{13}$C NMR ($CDCl_3$, 101 MHz) δ 206.7, 197.0, 153.1, 148.9, 134.7, 131.3, 123.2, 52.2, 43.9, 18.9; IR (neat, ZnSe, $cm^{-1}$) 2978, 2843, 1779, 1687, 1585, 1420, 1373, 1332, 1234, 1213, 1100, 1043, 1026, 997, 802, 704; HRMS (ESI/Q-TOF) m/z: $[M + Na]^+$ calcd for $C_{11}H_{11}NNaO_2$ 212.0688; found 212.0690.

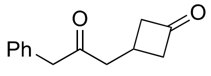


**3n**

**3-(2-Oxo-3-phenylpropyl)cyclobutan-1-one (3n).** Following the above procedure with **S1n** (751.0 mg, 3.000 mmol), IBX (1.2601 g, 4.5000 mmol), and *p*-TsOH•$H_2O$ (2.8530 g, 14.998 mmol). The crude product was purified by flash column chromatography to afford **3n** as a pale yellow solid (515.8 mg, 85%). $R_f$ = 2.4/4.0 (25% EtOAc in hexane); $^1$H NMR (400 MHz, $CDCl_3$) δ 7.36-7.31 (m, 2H), 7.29-7.25 (m, 1H), 7.21-7.18 (m, 2H), 3.69 (s, 2H), 3.25-3.16 (m, 2H), 2.79-2.77 (m, 2H), 2.76-2.67 (m, 1H), 2.65-2.57 (m, 2H); $^{13}$C NMR ($CDCl_3$, 101 MHz) δ 207.3, 206.6, 133.7, 129.3, 128.8, 127.2, 52.6, 50.3, 47.3, 19.3; IR (neat, ZnSe, $cm^{-1}$)3026, 2960, 2868, 2360, 1750, 1654, 1603, 1495, 1337, 1120, 1031, 950, 764, 703; HRMS (ESI/Q-TOF) m/z: $[M + Na]^+$ calcd for $C_{13}H_{14}NaO_2$ 225.0894; found 225.0892.

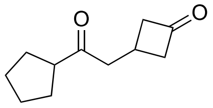

3o

**3-(2-Cyclopentyl-2-oxoethyl)cyclobutan-1-one (3o).** Following the above procedure with **S1o** (685.0 mg, 3.000 mmol), IBX (1.2601 g, 4.5000 mmol), and *p*-TsOH•$H_2O$ (2.8530 g, 14.998 mmol). The crude product was purified by flash column chromatography to afford **3o** as a pale yellow solid (292.5 mg, 54%). $R_f$ = 2.3/4.0 (25% EtOAc in hexane); $^1$H NMR (400 MHz, $CDCl_3$) δ 3.29-3.22 (m, 2H), 2.90-2.82 (m, 1H), 2.80-2.74 (m, 3H), 2.72-2.64 (m, 2H), 1.85-1.77 (m, 2H), 1.75-1.55 (m, 6H); $^{13}$C NMR ($CDCl_3$, 101 MHz) δ 211.5, 207.7, 52.8, 51.4, 47.4, 28.9, 25.9, 19.4; IR (neat, ZnSe, cm$^{-1}$) 2956, 2869, 1784, 1705, 1451, 1374, 1315, 1208, 1095; HRMS (ESI/Q-TOF) m/z: $[M + Na]^+$ calcd for $C_{11}H_{16}NaO_2$ 203.1048; found 203.1051.

Part II: Preliminary Results for the Tandem Pinacol Coupling/Pinacol Rearrangement of Diones

With the optimized conditions, the substrate scope was investigated (**Table S1**). While the yield of **5i** was similar to that of the model substrate, **5b** was obtained with a relatively low yield (entries 1-3). Based on our previous experience with the pinacol rearrangement reaction, we suspected that the pinacol coupling reaction could be the problematic step. Indeed, when the pinacol reaction was examined for **3b**, the yield of **5b** was 46% (with full conversion of **3b**). Fortunately, we found that by increasing the reaction temperature to 50 °C, **5b** was obtained with a yield of 63%. As such, we reevaluated diones **3a**, **3b**, and **3i** with the higher reaction temperature for the first step, and while virtually no change was observed for **5a**, the yields of **5b** and **5i** were improved (entries 4-6). With these results, we moved forward with the revised conditions.

**Table S1.** Preliminary Substrate Scope for the Tandem Pinacol Coupling/Pinacol Rearrangement of Diones **3a**, **3b**, and **3i**

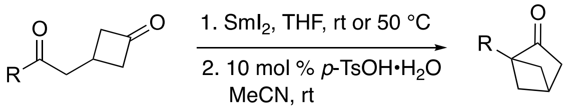


| entry | starting material | R | temperature | product | yield (%)[a] |
|---|---|---|---|---|---|
| 1 | **3a** | Ph | rt | **5a** | 65 |
| 2 | **3b** | 4-Cl-$C_6H_4$ | rt | **5b** | 20 |
| 3 | **3i** | 4-MeO-$C_6H_4$ | rt | **5i** | 64 |
| 4 | **3a** | Ph | 50 °C | **5a** | 63 |
| 5 | **3b** | 4-Cl-$C_6H_4$ | 50 °C | **5b** | 40 |
| 6 | **3i** | 4-MeO-$C_6H_4$ | 50 °C | **5i** | 82 |

[a] Yields of isolated products **5a,b,i** were based on **3a,b,i** over two steps.

Regarding the general trend of lower yields observed with aryl substituents bearing electron-withdrawing groups, it was suspected that the pinacol reaction was the problematic step. This suspicion was confirmed when the pinacol coupling was examined for **3b**, **3f**, and **3i**, revealing that the aryl group with the strongest electron-withdrawing group (R = $CF_3$) produced the lowest amount of vicinal diol (**Table S2**).

**Table S2.** $SmI_2$-Mediated Pinacol Coupling of Diones **3b**, **3f**, and **3i**

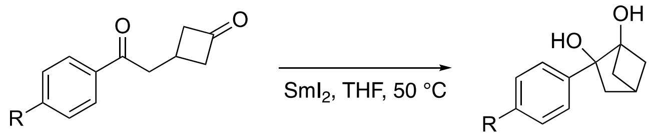


**3** **4**

| entry | starting material | R | product | Conv. (%)[a] | Yield (%)[a] |
|---|---|---|---|---|---|
| 1 | **3b** | Cl | **4b** | >95 | 63 |
| 2 | **3f** | $CF_3$ | **4f** | >95 | <27 |
| 3 | **3i** | OMe | **4i** | >95 | 75 |

[a] Yield determined by $^1H$ NMR analysis using triphenylmethane as an internal standard.

Part III: Substrate Scope for the Synthesis of **5a-o** via the Tandem Pinacol Coupling/Pinacol Rearrangement of Diones **3a-o**

General Procedure

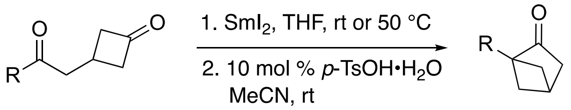


**3a-o** **5a-o**

In a flame-dried two-neck round bottom flask under a balloon of dry nitrogen gas was added $SmI_2$ (20.0 mL, 0.100 M, 2.00 mmol). This mixture was either kept at room temperature or placed in an oil bath and heated to 50 °C. Next, diketone **3a-o** (0.5000 mmol) was dissolved in THF (5.0 mL) and the resulting solution was added dropwise via syringe to the $SmI_2$ solution. After stirring for 0.5 h, saturated $NH_4Cl$ aqueous solution was added and the resulting mixture was extracted with DCM (several drops of 1N HCl were added to break the emulsion). The combined organic fractions were washed with brine, dried over $MgSO_4$, and then concentrated under reduced pressure. Without further purification, the crude product was placed in a round bottom flask and was dissolved in MeCN (1.0 mL). Next, *p*-TsOH•$H_2O$ (9.5 mg, 0.050 mmol, 10 mol %) was added and the reaction mixture was stirred for 0.5 h. Then the reaction was quenched with water and the resulting biphasic mixture was extracted with EtOAc. The combined organic fractions were washed with brine, dried over $MgSO_4$, and then concentrated under reduced pressure. The crude product was purified by column chromatography (silica gel) using EtOAc:hexane (8:92) as the eluent.

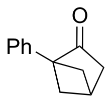

**5a**

**1-Phenylbicyclo[2.1.1]hexan-2-one (5a).** Following the above procedure with **3a** (94.1 mg, 0.500 mmol), $SmI_2$ (20.0 mL, 0.100 M, 2.00 mmol), and *p*-TsOH•$H_2O$ (9.5 mg, 0.050 mmol). The crude product was purified by flash column chromatography to afford **5a** as a white solid (55.0 mg, 63%). $R_f$ = 2.2/4.0 (25% EtOAc in hexane); $^1H$ NMR (400 MHz, $CDCl_3$) δ 7.38-7.33 (m, 2H), 7.30-7.25 (m, 1H), 7.16-7.13 (m, 2H), 2.86 (sep, *J*

= 1.80 Hz, 1H), 2.45-2.40 (m, 4H), 2.12-2.10 (m, 2H); $^{13}$C NMR ($CDCl_3$, 101 MHz) δ 211.6, 136.8, 128.2, 127.0, 126.6, 68.0, 44.8, 42.4, 30.9. The spectroscopic data are consistent with those reported previously.[2]

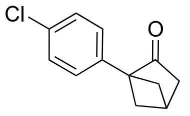

5b

**1-(4-Chlorophenyl)bicyclo[2.1.1]hexan-2-one (5b).** Following the above procedure with **3b** (111.3 mg, 0.4998 mmol), $SmI_2$ (20.0 mL, 0.100 M, 2.00 mmol), and *p*-TsOH•$H_2O$ (9.5 mg, 0.050 mmol). The crude product was purified by flash column chromatography to afford **5b** as a white solid (41.0 mg, 40%). $R_f$ = 2.3/4.0 (25% EtOAc in hexane); $^1$H NMR (400 MHz, $CDCl_3$) δ 7.31 (d, *J* = 8.29 Hz, 2H), 7.07 (d, *J* = 8.72 Hz, 2H), 2.86 (sep, *J* = 1.54 Hz, 1H), 2.42-2.39 (m, 4H), 2.09-2.07 (m, 2H); $^{13}$C NMR ($CDCl_3$, 101 MHz) δ 211.2, 135.3, 133.0, 128.5, 128.1, 67.4, 44.8, 42.3, 30.9; IR (neat, ZnSe, cm$^{-1}$) 2999, 2976, 2955, 2884, 1749, 1494, 1399, 1252, 1087, 1004, 826, 728; HRMS (ESI/Q-TOF) m/z: [M + Na]$^+$ calcd for $C_{12}H_{11}ClNaO$ 229.0396; found 229.0397.

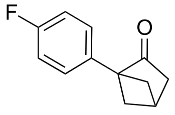

5c

**1-(4-Fluorophenyl)bicyclo[2.1.1]hexan-2-one (5c).** Following the above procedure with **3c** (103.1 mg, 0.5000 mmol), $SmI_2$ (20.0 mL, 0.100 M, 2.00 mmol), and *p*-TsOH•$H_2O$ (9.5 mg, 0.050 mmol). The crude product was purified by flash column chromatography to afford **5c** as a white solid (58.6 mg, 65%). $R_f$ = 2.2/4.0 (25% EtOAc in hexane); $^1$H NMR (400 MHz, $CDCl_3$) δ 7.13-7.08 (m, 2H), 7.05-6.99 (m, 2H), 2.85 (sep, *J* = 1.63 Hz, 1H), 2.42-2.38 (m, 4H), 2.08-2.06 (m, 2H); $^{13}$C NMR ($CDCl_3$, 101 MHz) δ 211.4, 161.9 (d, *J* = 245.1 Hz), 132.5 (d, *J* = 2.8 Hz), 128.3 (d, *J* = 8.2 Hz), 115.1 (d, *J* = 21.5 Hz), 67.2, 44.8, 42.2, 30.8; $^{19}$F NMR ($CDCl_3$, 376 MHz) δ -115.4. The spectroscopic data are consistent with those reported previously.[2]

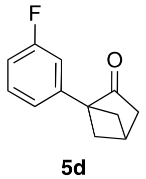

5d

**1-(3-Fluorophenyl)bicyclo[2.1.1]hexan-2-one (5d).** Following the above procedure with **3d** (103.1 mg, 0.5000 mmol), $SmI_2$ (20.0 mL, 0.100 M, 2.00 mmol), and *p*-TsOH•$H_2O$ (9.5 mg, 0.050 mmol). The crude product was purified by flash column chromatography to afford **5d** as a white solid (50.0 mg, 57%). $R_f$ = 2.1/4.0 (25% EtOAc in hexane); $^1$H NMR (400 MHz, $CDCl_3$) δ 7.33-7.27 (m, 1H), 6.97-6.92 (m, 1H), 6.92-6.89 (m, 1H), 6.86-6.83 (m, 1H), 2.86 (sep, *J* = 1.60 Hz, 1H), 2.43-2.39 (m, 4H), 2.09-2.08 (m, 2H); $^{13}$C NMR ($CDCl_3$, 101 MHz) δ 210.9, 162.8 (d, *J* = 260.1Hz), 139.3 (d, *J* = 7.7 Hz), 129.8 (d, *J* = 8.4 Hz), 122.3 (d, *J* = 2.6 Hz), 113.9 (d, *J* = 16.4 Hz), 113.7 (d, *J* = 16.7 Hz), 67.5, 44.8, 42.3, 30.8; $^{19}$F NMR ($CDCl_3$, 376 MHz) δ -113.6; IR (neat, ZnSe, cm$^{-1}$) 2985, 2963,2938, 2881, 1751, 1617, 1584, 1442, 1254, 1214, 1158, 1100, 894, 798, 694; HRMS (ESI/Q-TOF) m/z: [M + Na]$^+$ calcd for $C_{12}H_{11}FNaO$ 213.0692; found 213.0693.

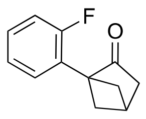

5e

**1-(2-Fluorophenyl)bicyclo[2.1.1]hexan-2-one (5e).** Following the above procedure with **3e** (103.1 mg, 0.5000 mmol), $SmI_2$ (20.0 mL, 0.100 M, 2.00 mmol), and *p*-TsOH•$H_2O$ (9.5 mg, 0.050 mmol). The crude product was purified by flash column chromatography to afford **5e** as a white solid (38 mg, 40%). $R_f$ = 2.4/4.0 (25% EtOAc in hexane); $^1$H NMR (400 MHz, $CDCl_3$) δ 7.28-7.22 (m, 1H), 7.10 (td, *J* = 7.32 Hz,

$J$ = 1.28 Hz, 1H), 7.06-6.99 (m, 1H), 6.86-6.83 (m, 1H), 2.89 (sep, $J$ = 1.37 Hz, 1H), 2.46-2.42 (m, 2H), 2.40-2.38 (m, 2H), 2.20-2.18 (m, 2H); $^{13}$C NMR ($CDCl_3$, 101 MHz) δ 210.1, 161.1 (d, $J$ = 246.7 Hz), 129.0 (d, $J$ = 7.8 Hz), 128.7 (d, $J$ = 5.0 Hz), 124.2 (d, $J$ = 15.8 Hz), 123.8 (d, $J$ = 3.0 Hz), 115.5 (d, $J$ = 20.1 Hz), 65.5, 44.6, 41.8, 32.0; $^{19}$F NMR ($CDCl_3$, 376 MHz) δ -116.1; IR (neat, ZnSe, $cm^{-1}$) 2987, 2957, 2885, 1759, 1616, 1585, 1497, 1450, 1254, 1217, 1117, 1005, 900, 768, 755; HRMS (ESI/Q-TOF) m/z: $[M + Na]^+$ calcd for $C_{12}H_{11}FNaO$ 213.0692; found 213.0693.

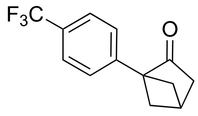

**5f**

**1-(4-(Trifluoromethyl)phenyl)bicyclo[2.1.1]hexan-2-one (5f).** Following the above procedure with **3f** (128.1 mg, 0.5000 mmol), $SmI_2$ (20.0 mL, 0.100 M, 2.00 mmol), and *p*-TsOH•$H_2O$ (9.5 mg, 0.050 mmol). The crude product was purified by flash column chromatography to afford **5f** as a white solid (16.0 mg, 12%). $R_f$ = 2.1/4.0 (25% EtOAc in hexane); $^1$H NMR (400 MHz, $CDCl_3$) δ 7.60 (d, $J$ = 8.04 Hz, 2H), 7.25 (d, $J$ = 8.23 Hz, 2H), 2.92-2.88 (m, 1H), 2.48-2.42(m, 4H), 2.14 (dd, $J$ = 4.63 Hz, $J$ = 1.60 Hz, 2H); $^{13}$C NMR ($CDCl_3$, 101 MHz) δ 210.8, 140.8, 129.3 (q, $J$ = 31.9 Hz), 127.1, 125.2 (q, $J$ = 3.5 Hz), 124.1 (q, $J$ = 271.8 Hz), 67.6, 44.8, 42.2, 31.0; $^{19}$F NMR ($CDCl_3$, 376 MHz) δ -63.8; IR (neat, ZnSe, $cm^{-1}$) 1749, 1618, 1323, 1275, 1130, 1120, 1104, 1067, 1041, 1004, 1018, 901, 837; HRMS (ESI/Q-TOF) m/z: $[M + H]^+$ calcd for $C_{13}H_{12}F_3O$ 241.0840; found 241.0840.

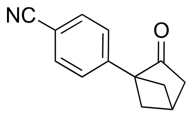

**5g**

**4-(2-Oxobicyclo[2.1.1]hexan-1-yl)benzonitrile (5g).** Following the above procedure with **3g** (106.6 mg, 0.4999 mmol), $SmI_2$ (20.0 mL, 0.100 M, 2.00 mmol), and *p*-TsOH•$H_2O$ (9.5 mg, 0.050 mmol). The crude product was purified by flash column chromatography to afford **5g** as a white solid (16.0 mg, 16%). $R_f$ = 2.2 /4.0 (25% EtOAc in hexane); $^1$H NMR (400 MHz, $CDCl_3$) δ 7.63 (dt, $J$ = 8.44 Hz, $J$ = 2.01 Hz, 2H), 7.23 (dt, $J$ = 8.16 Hz, $J$ = 1.61 Hz, 2H), 2.90 (sep, $J$ = 1.83 Hz, 1H), 2.46-2.43 (m, 4H), 2.13-2.11 (m, 2H); $^{13}$C NMR ($CDCl_3$, 101 MHz) δ 210.3, 142.3, 132.1, 127.6, 118.8, 110.9, 67.7, 44.8, 42.2, 31.1; IR (neat, ZnSe, $cm^{-1}$) 2983, 2963, 2931, 2227, 1748, 1612, 1515, 1283, 1255, 1185, 1172, 1101, 1004, 903, 836, 760; HRMS (ESI/Q-TOF) m/z: $[M + Na]^+$ calcd for $C_{13}H_{11}NNaO$ 220.0738; found 220.0736.

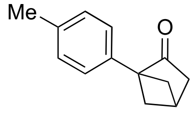

**5h**

**1-(*p*-Tolyl)bicyclo[2.1.1]hexan-2-one (5h).** Following the above procedure with **3h** (101.1 mg, 0.4999 mmol), $SmI_2$ (20.0 mL, 0.100 M, 2.00 mmol), and *p*-TsOH•$H_2O$ (9.5 mg, 0.050 mmol). The crude product was purified by flash column chromatography to afford **5h** as a white solid (75.2 mg, 80%). $R_f$ = 2.2/4.0 (25% EtOAc in hexane); $^1$H NMR (400 MHz, $CDCl_3$) δ 7.17 (d, $J$ = 7.54 Hz, 2H), 7.04 (d, $J$ = 7.93 Hz, 2H), 2.85 (sep, $J$ = 1.65 Hz, 1H), 2.42-2.39 (m, 4H), 2.35 (s, 3H), 2.09 (dd, $J$ = 4.77 Hz, $J$ = 2.20 Hz, 2H); $^{13}$C NMR ($CDCl_3$, 101 MHz) δ211.8, 136.6, 133.7, 129.0, 126.4, 67.8, 44.8, 42.3, 30.8, 21.1. The spectroscopic data are consistent with those reported previously.[2]

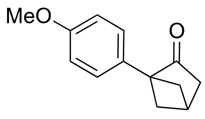

5i

**1-(4-Methoxyphenyl)bicyclo[2.1.1]hexan-2-one (5i).** Following the above procedure with **3i** (109.1 mg, 0.4999 mmol), $SmI_2$ (20.0 mL, 0.100 M, 2.00 mmol), and *p*-TsOH•$H_2O$ (9.5 mg, 0.050 mmol). The crude product was purified by flash column chromatography to afford **5i** as a white solid (84.0 mg, 82%). $R_f$ = 2.3/4.0 (25% EtOAc in hexane); $^1$H NMR (400 MHz, $CDCl_3$) δ 7.08 (d, *J* = 8.52 Hz, 2H), 6.89 (d, *J* = 8.52 Hz, 2H), 3.79 (s, 3H), 2.83 (sep, *J* = 1.74 Hz, 1H), 2.41-2.38 (m, 4H), 2.06 (dd, *J* = 4.76 Hz, *J* = 2.38 Hz, 2H); $^{13}$C NMR ($CDCl_3$, 101 MHz) δ 211.9, 158.6, 128.8, 127.7, 113.7, 77.2, 67.3, 55.1, 44.8, 42.3, 30.7; IR (neat, ZnSe, $cm^{-1}$) 3036, 2951, 2880, 2836, 1754, 1614, 1578, 1519, 1464, 1296, 1178, 1097, 1030, 1014, 896, 828, 758; HRMS (ESI/Q-TOF) m/z: [M + Na]$^+$ calcd for $C_{13}H_{14}NaO_2$ 225.0892; found 225.0894.

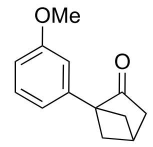

5j

**1-(3-Methoxyphenyl)bicyclo[2.1.1]hexan-2-one (5j).** Following the above procedure with **3j** (109.1 mg, 0.4999 mmol), $SmI_2$ (20.0 mL, 0.100 M, 2.00 mmol), and *p*-TsOH•$H_2O$ (9.5 mg, 0.050 mmol). The crude product was purified by flash column chromatography to afford **5j** as a white solid (56.0 mg, 56%). $R_f$ = 2.2/4.0 (25% EtOAc in hexane); $^1$H NMR (400 MHz, $CDCl_3$) δ 7.27 (dd, *J* = 8.20 Hz, *J* = 7.85 Hz, 1H), 6.81 (ddd, *J* = 8.21 Hz, *J* = 2.56 Hz, *J* = 1.02 Hz, 1H), 6.73-6.71 (m, 1H), 6.68-6.67 (m, 1H), 3.76 (s, 3H), 2.84 (sep, *J* = 1.48 Hz, 1H), 2.36-2.33 (m, 4H), 2.22 (dd, *J* = 4.76 Hz, *J* = 2.06 Hz, 2H); $^{13}$C NMR ($CDCl_3$, 101 MHz) δ 211.4, 159.5, 138.4, 129.3, 118.9, 112.5, 112.4, 67.9, 55.1, 44.8, 42.4, 30.8; IR (neat, ZnSe, $cm^{-1}$) 2957, 2924, 2885, 2854, 1752, 1582, 1451, 1349, 1288, 1228, 1161, 1100, 1037, 807, 782, 696; HRMS (ESI/Q-TOF) m/z: [M + Na]$^+$ calcd for $C_{13}H_{14}NaO_2$ 225.0892; found 225.0890.

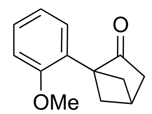

5k

**1-(2-Methoxyphenyl)bicyclo[2.1.1]hexan-2-one (5k).** Following the above procedure with **3k** (109.1 mg, 0.4999 mmol), $SmI_2$ (20.0 mL, 0.100 M, 2.00 mmol), and *p*-TsOH•$H_2O$ (9.5 mg, 0.050 mmol). The crude product was purified by flash column chromatography to afford **5k** as a white solid (62.6 mg, 62%). $R_f$ = 2.1/4.0 (25% EtOAc in hexane); $^1$H NMR (400 MHz, $CDCl_3$) δ 7.27-7.23 (m, 1H), 6.94-6.87 (m, 3H), 3.76 (s, 3H), 2.84 (sep, *J* = 1.48 Hz, 1H), 2.36-2.33 (m, 4H), 2.22 (dd, *J* = 4.76 Hz, *J* = 2.06 Hz, 2H); $^{13}$C NMR ($CDCl_3$, 101 MHz) δ 210.9, 158.0, 128.6, 128.0, 125.7, 120.3, 110.8, 67.1, 55.4, 44.8, 42.3, 31.7; IR (neat, ZnSe, $cm^{-1}$) 2981, 2958, 2927, 1754, 1601, 1495, 1465, 1242, 1178, 1128, 1025,1004,899, 773, 758; HRMS (ESI/Q-TOF) m/z: [M + H]$^+$ calcd for $C_{13}H_{15}O_2$ 203.1072; found 203.1071.

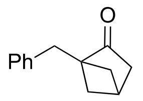

5n

**1-Benzylbicyclo[2.1.1]hexan-2-one (5n).** Following the above procedure with **3n** (101.1 mg, 0.4999 mmol), $SmI_2$ (20.0 mL, 0.100 M, 2.00 mmol), and *p*-TsOH•$H_2O$ (9.5 mg, 0.050 mmol). The crude product was purified by flash column chromatography to afford **5n** as a colorless liquid (40.5 mg, 42%). $R_f$ = 3.0/4.0 (25% EtOAc in hexane); $^1$H NMR (400 MHz, $CDCl_3$) δ 7.30-7.26 (m, 2H), 7.23-7.18 (m, 1H), 7.14-7.12 (m, 2H), 2.87 (s, 2H), 2.67-2.65 (m, 1H), 2.22-2.21 (m, 2H), 1.90-1.86 (m, 2H), 1.54-1.52 (m, 2H); $^{13}$C NMR ($CDCl_3$, 101 MHz) δ 214.0, 138.8, 129.5, 128.2, 126.0, 65.8, 42.8, 42.5, 33.7, 31.3, 29.6;

IR (neat, ZnSe, cm$^{-1}$) 3027, 2924, 2854, 1755, 1602, 1495, 1454, 1084, 1020. 748. 701; HRMS (ESI/Q-TOF) m/z: $[M + Na]^+$ calcd for $C_{13}H_{14}NaO$ 209.0942; found 209.0941.

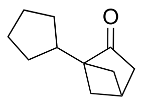

5o

**1-Cyclopentylbicyclo[2.1.1]hexan-2-one (5o).** Following the above procedure with **3o** (90.1 mg, 0.500 mmol), $SmI_2$ (20.0 mL, 0.100 M, 2.00 mmol), and *p*-TsOH•$H_2O$ (9.5 mg, 0.050 mmol). The crude product was purified by flash column chromatography to afford **5o** as a white solid (36.2 mg, 45%). $R_f$ = 2.3 /4.0 (25% EtOAc in hexane); $^1$H NMR (400 MHz, $CDCl_3$) δ 2.69 (m, 1H), 2.16-2.14 (m, 2H), 2.12-2.06 (m, 1H), 1.77-1.69 (m, 2H), 1.76-1.68 (m, 2H), 1.58-1.48 (m, 6H), 1.18-1.08 (m, 2H); $^{13}$C NMR ($CDCl_3$, 101 MHz) δ 214.7, 68.5, 42.9, 41.3, 36.8, 30.7, 28.7, 25.3; IR (neat, ZnSe, cm$^{-1}$) 2949, 2868, 1752, 1451, 1419, 1273, 1250, 1088, 1016, 888, 765; HRMS (ESI/Q-TOF) m/z: $[M + H]^+$ calcd for $C_{11}H_{17}O$ 165.1279; found 165.1282.

Part IV: Larger Scale Synthesis of Bicyclic Ketone **5n**

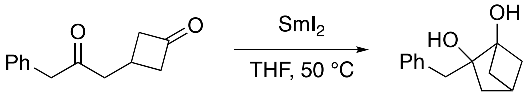


3n 4n

**2-Benzylbicyclo[2.1.1]hexane-1,2-diol.** In a flame-dried two-neck round bottom flask under a balloon of dry nitrogen gas was added $SmI_2$ (100.0 mL, 0.1000 M, 10.00 mmol). This mixture was placed in an oil bath and heated to 50 °C. Next, diketone **3n** (485.4 mg, 2.400 mmol) was dissolved in THF (25.0 mL) and the resulting solution was added dropwise via syringe to the $SmI_2$ solution. After stirring for 2 h, saturated $NH_4Cl$ aqueous solution was added and the resulting mixture was extracted with DCM (several drops of 1N HCl were added to break the emulsion). The combined organic fractions were washed with brine, dried over $MgSO_4$, and then concentrated under reduced pressure. The crude product was purified by column chromatography (silica gel) using EtOAc:hexane (20:80) as the eluent to afford **4n** as a colorless liquid (272.1 mg, 55%). $R_f$ = 0.8/4.0 (25% EtOAc in hexane); $^1$H NMR (400 MHz, $CDCl_3$) δ 7.34 -7.23 (m, 5H), 3.12 (d, $J$ = 14.00 Hz, 1H), 2.87 (d, $J$ = 14.00 Hz, 1H), 2.69 (br s, 2H), 2.20-2.17 (m, 1H), 2.04 (dd, $J$ = 9.48, 6.21 Hz, 1H), 2.00-1.96 (m, 1H), 1.78-1.74 (m, 1H), 1.71-1.67 (m, 1H), 1.60-1.56 (m, 1H), 1.41-1.37 (m, 1H); $^{13}$C NMR ($CDCl_3$, 101 MHz) δ 137.8, 130.2, 128.3, 126.4, 83.2, 76.9, 43.5, 42.8, 42.7, 41.2; IR (neat, ZnSe, cm$^{-1}$) 3428, 3061, 3026, 2958, 1604, 1496, 1453, 1263, 1090, 994, 960, 891, 781, 736, 702; HRMS (EI) m/z: $[M]^+$ calcd for $C_{13}H_{16}NaO_2$ 227.1048; found 227.1049.

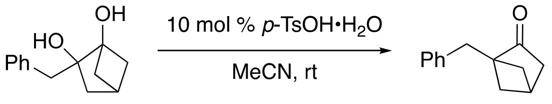


4n 5n

**1-Benzylbicyclo[2.1.1]hexan-2-one (5n).** In a round bottom flask, **4n** (272.1 mg, 1.329 mmol) was dissolved in MeCN (13.0 mL). Next, *p*-TsOH•$H_2O$ (25.3 mg, 0.133 mmol, 10 mol %) was

added and the reaction mixture was stirred for 4 h. Then the reaction was quenched with water and the resulting biphasic mixture was extracted with EtOAc. The combined organic fractions were washed with brine, dried over $MgSO_4$, and then concentrated under reduced pressure. The crude product was purified by column chromatography (silica gel) using EtOAc:hexane (8:92) as the eluent to afford **5n** as a colorless oil (241.9 mg, 98%).

Part V: Synthesis of Saturated Analog of Nitazoxanide

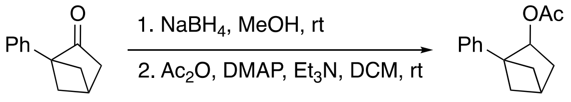


**5a** **6**

**1-Phenylbicyclo[2.1.1]hexan-2-yl acetate (6).** In a round-bottom flask, **5a** (344.4 mg, 2.000 mmol) was dissolved in MeOH (20 mL), followed by the addition of $NaBH_4$ (151.3 mg, 4.000 mmol). After stirring overnight at room temperature, the reaction mixture was quenched with water and diluted with EtOAc. The resulting biphasic mixture was extracted with EtOAc and the combined organic fractions were washed with brine, dried over $MgSO_4$, and then concentrated under reduced pressure. Without further purification, the crude product was placed in the round bottom flask with acetic anhydride (0.95 mL, 10 mmol), triethyl amine (1.4 mL, 10 mmol), DMAP (293.0 mg, 2.398 mmol), and DCM (4.0 mL) and stirred for overnight at room temperature. Then, water was added and the resulting biphasic mixture was extracted with DCM. The combined organic fractions were washed with brine, dried over $MgSO_4$, and then concentrated under reduced pressure. The crude product was purified by column chromatography (silica gel) using EtOAc:hexane (5:95) as the eluent to afford **6** as a white solid (421.2 mg, 93% for two steps). $R_f$ = 2.5/4.0 (25% EtOAc in hexane); $^1H$ NMR (400 MHz, $CDCl_3$) δ 7.32-7.28 (m, 2H), 7.24-7.19 (m, 3H), 5.24-5.21 (m, 1H), 2.52-2.50 (m, 1H), 2.46-2.40 (m, 1H), 2.10-1.97 (m, 1H), 1.96 (s, 3H), 1.93-1.90 (m, 1H), 1.85-1.82 (m, 1H), 1.66-1.57 (m, 2H); $^{13}C$ NMR ($CDCl_3$, 101 MHz) δ 171.1, 141.0. 128.0, 126.4, 126.3, 77.5, 57.3, 43.6, 39.1, 38.7, 34.8, 21.1. The spectroscopic data are consistent with those reported previously.[3]

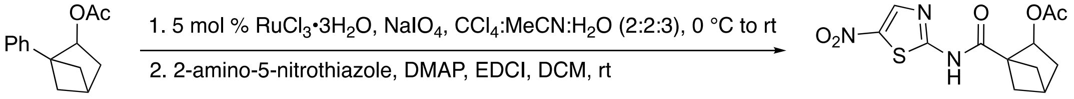


**6** **7**

**1-((5-Nitrothiazol-2-yl)carbamoyl)bicyclo[2.1.1]hexan-2-yl acetate (7).** To a round bottom flask containing **6** (421.7 mg, 1.950 mmol), tetrachloromethane (8.0 mL), acetonitrile (8.0 mL), and water (12.0 mL) was slowly added $NaIO_4$ (5.005 g, 23.40 mmol) at 0 ℃. After vigorously stirring at 0 ℃ for 0.5 h, $RuCl_3$•$3H_2O$ (26.2 mg, 0.100 mmol, 5 mol %) was added, and the reaction mixture was allowed to warm to ambient temperature. After vigorously stirring the mixture overnight, the reaction mixture was filtered through celite, and the filter cake was washed with DCM. The filtrate was then concentrated under reduced pressure to provide the desired carboxylic

acid. Without further purification, the crude acid was dissolved in DCM (10 mL), and then 2-amino-5-nitrothiazole (279.4 mg, 1.925 mmol), 1-ethyl-3-(3-dimethylaminopropyl)carbodiimide hydrochloride (326.0 mg, 2.100 mmol), and DMAP (42.7 mg, 0.350 mmol). After stirring overnight at room temperature, the reaction was quenched with water, extracted with DCM, dried over $Na_2SO_4$, and then concentrated under reduced pressure. The crude product was purified by column chromatography (silica gel) using EtOAc:hexane (20:80) as the eluent to afford **7** as a yellowish solid (223.3 mg, 37% for two steps). $R_f$ = 1.3/4.0 (25% EtOAc in hexane); $^1$H NMR (400 MHz, $CDCl_3$) δ 9.70 (br s, 1H), 8.28 (s, 1H), 5.45 (d, $J$ = 7.55 Hz, 1H), 2.55-2.54 (m, 1H), 2.45-2.40 (m, 1H), 2.16-2.10 (m, 5H), 1.94-1.90 (m, 1H), 1.70-1.66 (m, 1H), 1.59-1.55 (m, 1H); $^{13}$C NMR ($CDCl_3$, 101 MHz) δ 171.2, 169.3, 160.4, 143.7, 140.6, 74.8, 56.7, 42.1, 38.5, 37.8, 34.9, 21.1; IR (neat, ZnSe, $cm^{-1}$) 2992, 1718, 1690, 1535, 1478, 1429, 1354, 1304, 1243, 1210, 1177, 1127, 1102, 1070, 1042, 904, 813, 738; HRMS (EI) m/z: $[M]^+$ calcd for $C_{12}H_{13}N_3O_5S$ 311.0576; found 311.0579.

Part VI: References


1. Hsu, C.-W.; Wu, C.-F.; Lee, Y.-C.; Yoo, W.-J. *Adv. Synth. Catal.* **2024**, early view. doi: 10.1002/adsc.202400891.
2. Herter, L.; Koutsopetras, I.; Turelli, L.; Fessard, T.; Salomé, C. *Org. Biomol. Chem.* **2022**, *20*, 9108–9111.
3. Reinhold, M.; Steinebach, J.; Golz, C.; Walker, J. C. L. *Chem. Sci.* **2023**, *14*, 9885–9891.

Part VII: Copies of NMR Spectra Data

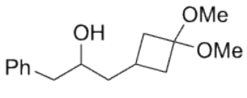

**S1n**

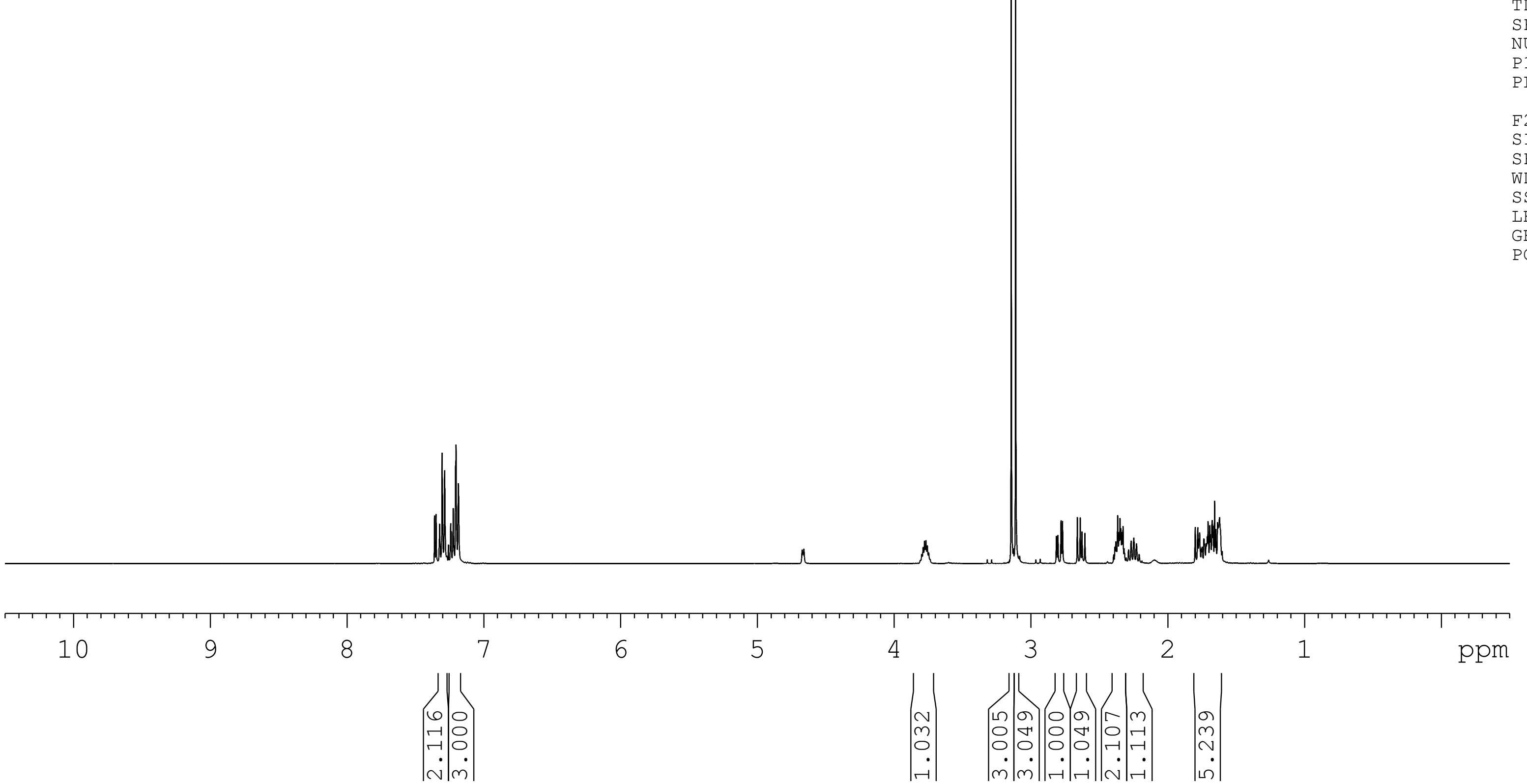


```
Current Data Parameters
NAME             G benzyl
EXPNO                   1
PROCNO                  1

F2 - Acquisition Parameters
Date_            20240618
Time                13.28 h
INSTRUM             spect
PROBHD   Z108618_0411 (
PULPROG              zg30
TD                  32768
SOLVENT             CDCl3
NS                     26
DS                      0
SWH              8802.817 Hz
FIDRES           0.537281 Hz
AQ              1.8612224 sec
RG                  51.61
DW                 56.800 usec
DE                  14.47 usec
TE                  298.0 K
D1             1.00000000 sec
TD0                     1
SFO1        400.1328009 MHz
NUC1                   1H
P1                  15.50 usec
PLW1           9.89999962 W

F2 - Processing parameters
SI                 131072
SF          400.1300095 MHz
WDW                    EM
SSB                     0
LB                      0 Hz
GB                      0
PC                   1.00
```

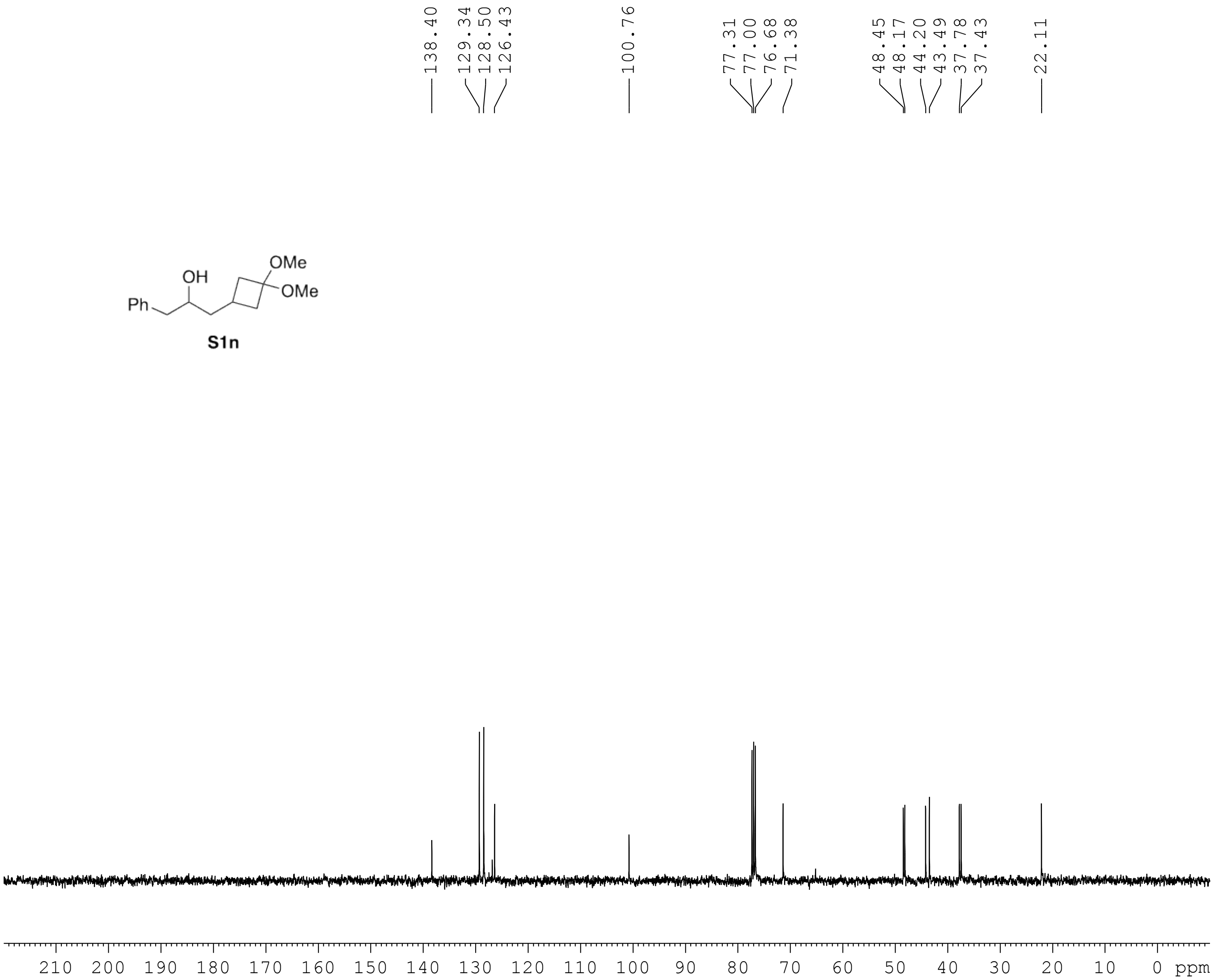


```
Current Data Parameters
NAME            G benzyl
EXPNO                  3
PROCNO                 1

F2 - Acquisition Parameters
Date_           20240618
Time               13.35 h
INSTRUM            spect
PROBHD   Z108618_0411 (
PULPROG          zgpg30
TD                 65536
SOLVENT            CDCl3
NS                    14
DS                     4
SWH            28409.092 Hz
FIDRES          0.866977 Hz
AQ             1.1534336 sec
RG                212.49
DW                17.600 usec
DE                  6.50 usec
TE                 298.0 K
D1            2.00000000 sec
D11           0.03000000 sec
TD0                    1
SFO1         100.6258487 MHz
NUC1                 13C
P1                 10.50 usec
PLW1         42.50000000 W
SFO2         400.1316005 MHz
NUC2                  1H
CPDPRG[2         waltz16
PCPD2              90.00 usec
PLW2          9.89999962 W
PLW12         0.29363999 W
PLW13         0.14747000 W

F2 - Processing parameters
SI                 32768
SF           100.6127754 MHz
WDW                   EM
SSB                    0
LB                  3.00 Hz
GB                     0
PC                  1.40
```

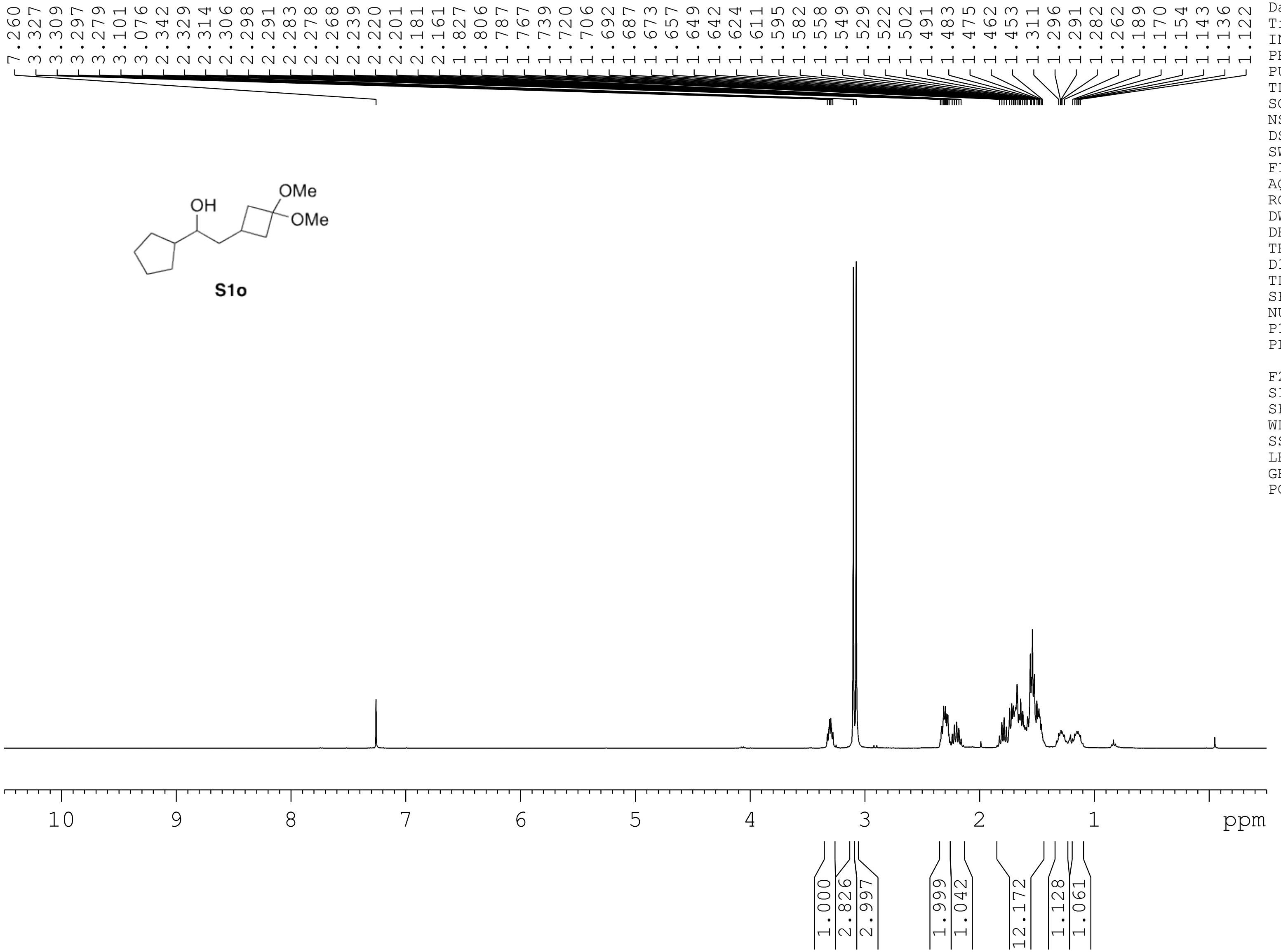


Current Data Parameters
NAME G-cyclopentyl H
EXPNO 1
PROCNO 1

F2 - Acquisition Parameters
Date_ 20231013
Time 17.21 h
INSTRUM spect
PROBHD Z108618_0411 (
PULPROG zg30
TD 32768
SOLVENT CDCl3
NS 19
DS 0
SWH 8802.817 Hz
FIDRES 0.537281 Hz
AQ 1.8612224 sec
RG 22.56
DW 56.800 usec
DE 14.47 usec
TE 298.8 K
D1 1.00000000 sec
TD0 1
SFO1 400.1328009 MHz
NUC1 1H
P1 15.50 usec
PLW1 9.89999962 W

F2 - Processing parameters
SI 131072
SF 400.1300094 MHz
WDW EM
SSB 0
LB 0 Hz
GB 0
PC 1.00

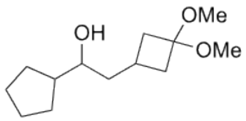

**S1o**

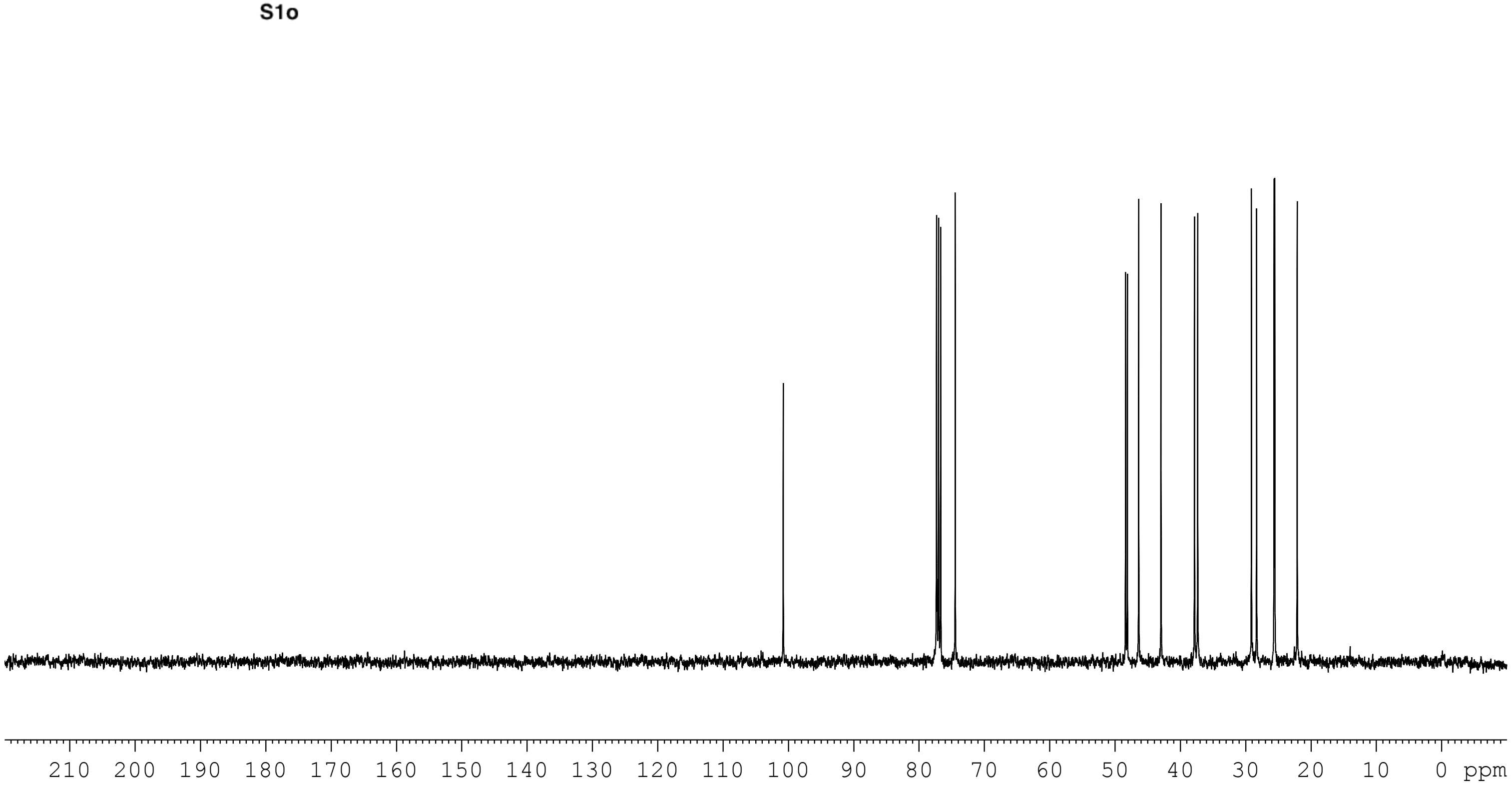


```
Current Data Parameters
NAME     G-cylopentyl C
EXPNO                 1
PROCNO                1

F2 - Acquisition Parameters
Date_          20231013
Time              17.26 h
INSTRUM           spect
PROBHD   Z108618_0411 (
PULPROG         zgpg30
TD               65536
SOLVENT          CDCl3
NS                  51
DS                   0
SWH          28409.092 Hz
FIDRES        0.866977 Hz
AQ           1.1534336 sec
RG              212.49
DW              17.600 usec
DE                6.50 usec
TE               298.7 K
D1          2.00000000 sec
D11         0.03000000 sec
TD0                  1
SFO1       100.6258487 MHz
NUC1               13C
P1               10.50 usec
PLW1       42.50000000 W
SFO2       400.1316005 MHz
NUC2                1H
CPDPRG[2       waltz16
PCPD2            90.00 usec
PLW2        9.89999962 W
PLW12       0.29363999 W
PLW13       0.14747000 W

F2 - Processing parameters
SI               32768
SF         100.6127749 MHz
WDW                 EM
SSB                  0
LB                3.00 Hz
GB                   0
PC                1.40
```

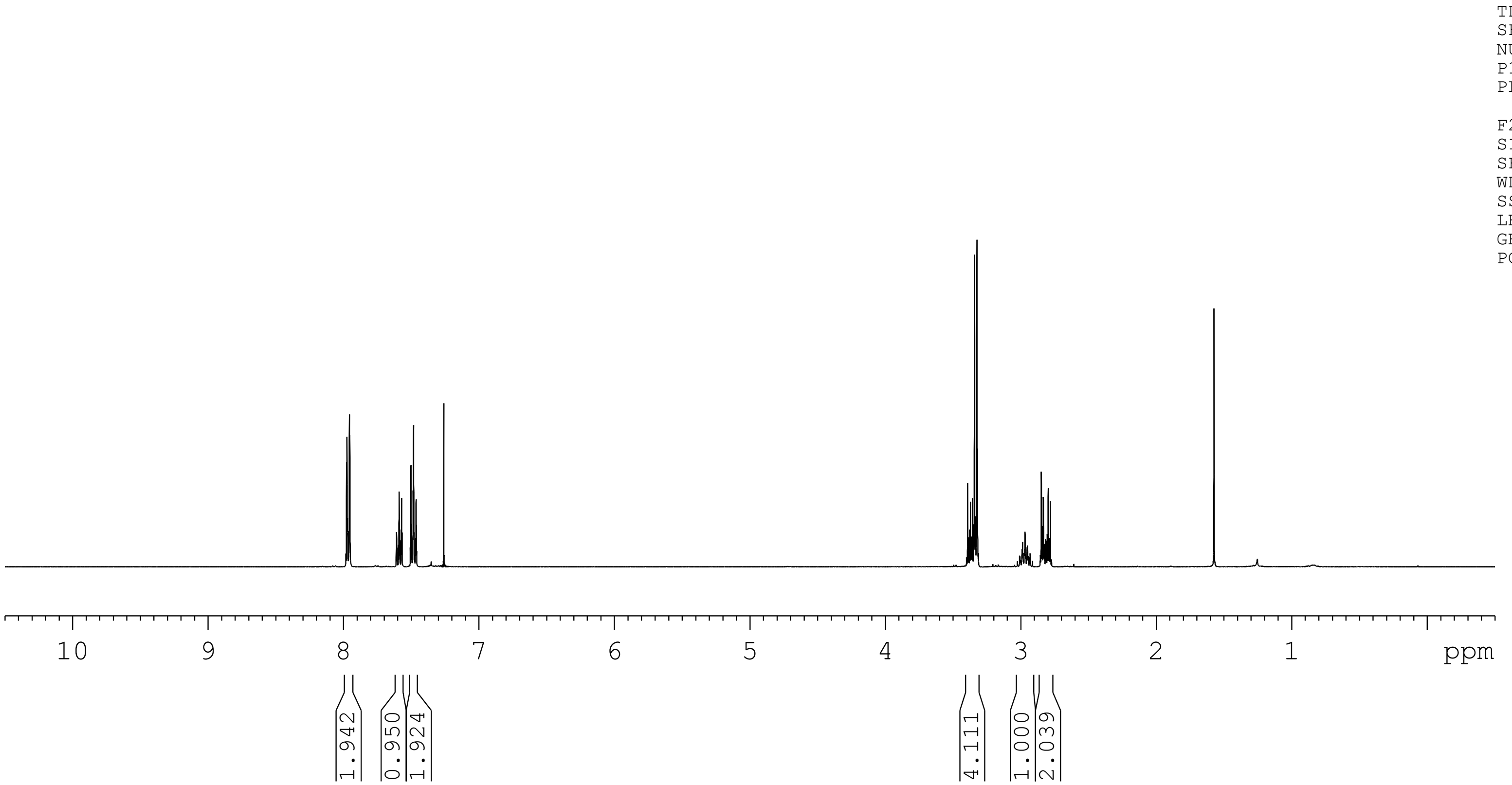


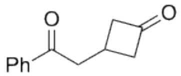


**3a**

Current Data Parameters
NAME K Ph H
EXPNO 1
PROCNO 1

F2 - Acquisition Parameters
Date_ 20230923
Time 15.21 h
INSTRUM spect
PROBHD Z108618_0411 (
PULPROG zg30
TD 32768
SOLVENT CDCl3
NS 27
DS 0
SWH 8802.817 Hz
FIDRES 0.537281 Hz
AQ 1.8612224 sec
RG 212.49
DW 56.800 usec
DE 14.47 usec
TE 298.0 K
D1 1.00000000 sec
TD0 1
SFO1 400.1328009 MHz
NUC1 1H
P1 15.50 usec
PLW1 9.89999962 W

F2 - Processing parameters
SI 131072
SF 400.1300096 MHz
WDW EM
SSB 0
LB 0 Hz
GB 0
PC 1.00

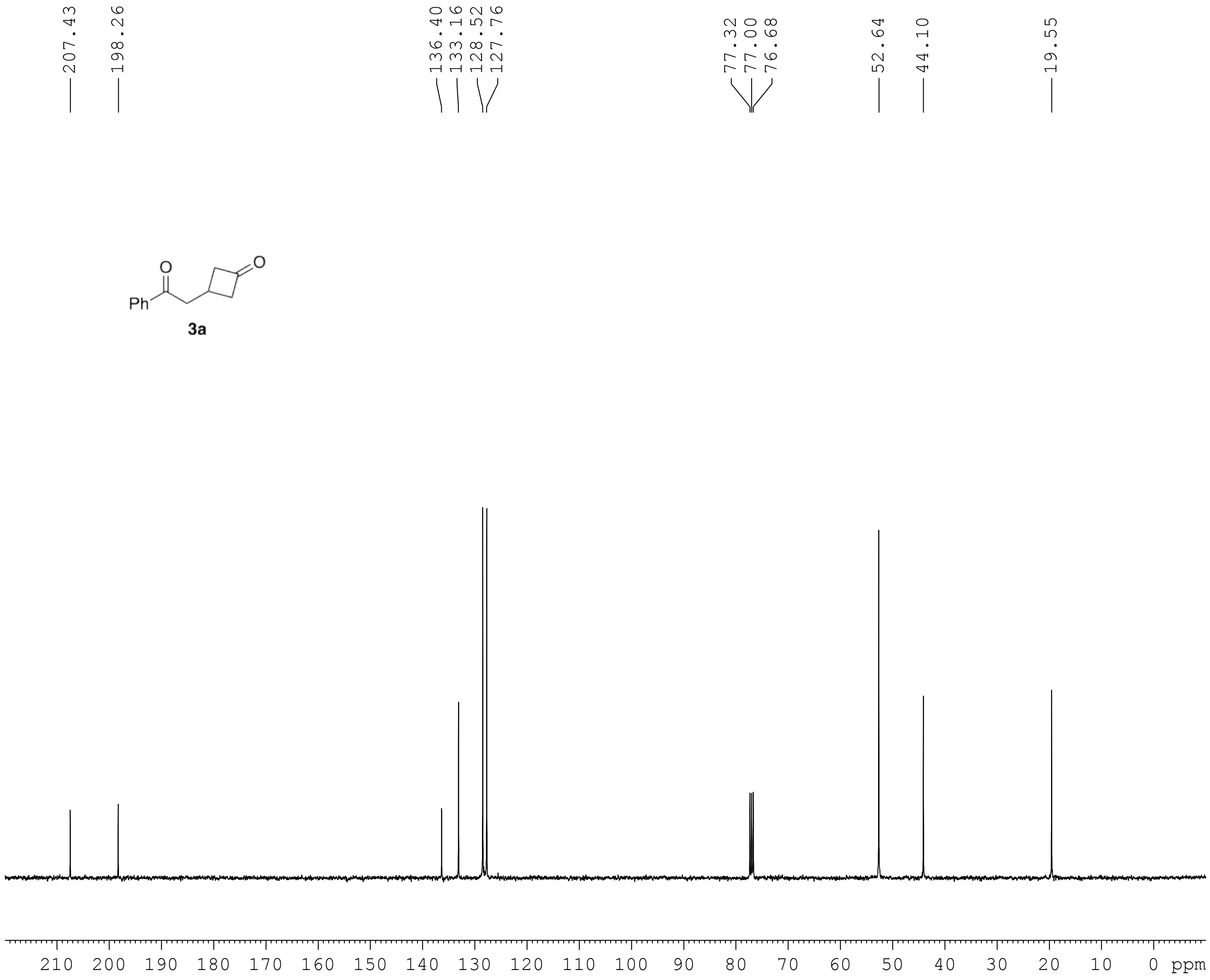


```
Current Data Parameters
NAME              K Ph C
EXPNO                  4
PROCNO                 1

F2 - Acquisition Parameters
Date_           20230922
Time               13.38 h
INSTRUM            spect
PROBHD   Z108618_0411 (
PULPROG          zgpg30
TD                 65536
SOLVENT            CDCl3
NS                    38
DS                     4
SWH           28409.092 Hz
FIDRES         0.866977 Hz
AQ            1.1534336 sec
RG                212.49
DW                17.600 usec
DE                  6.50 usec
TE                 298.1 K
D1            2.00000000 sec
D11           0.03000000 sec
TD0                    1
SFO1         100.6258487 MHz
NUC1                 13C
P1                 10.50 usec
PLW1         42.50000000 W
SFO2         400.1316005 MHz
NUC2                  1H
CPDPRG[2         waltz16
PCPD2              90.00 usec
PLW2          9.89999962 W
PLW12         0.29363999 W
PLW13         0.14747000 W

F2 - Processing parameters
SI                 32768
SF           100.6127877 MHz
WDW                   EM
SSB                    0
LB                  3.00 Hz
GB                     0
PC                  1.40
```

```
Current Data Parameters
NAME     20231012_4-Cl_oxidation
EXPNO                  3
PROCNO                 1

F2 - Acquisition Parameters
Date_          20231012
Time              20.28 h
INSTRUM           spect
PROBHD   Z108618_0411 (
PULPROG            zg30
TD                32768
SOLVENT           CDCl3
NS                   27
DS                    0
SWH            8802.817 Hz
FIDRES         0.537281 Hz
AQ            1.8612224 sec
RG               212.49
DW               56.800 usec
DE                14.47 usec
TE                298.7 K
D1           1.00000000 sec
TD0                   1
SFO1        400.1328009 MHz
NUC1                 1H
P1                15.50 usec
PLW1         9.89999962 W

F2 - Processing parameters
SI               131072
SF          400.1300098 MHz
WDW                  EM
SSB                   0
LB                    0 Hz
GB                    0
PC                 1.00
```

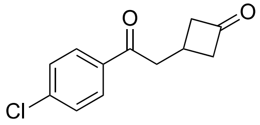


3b

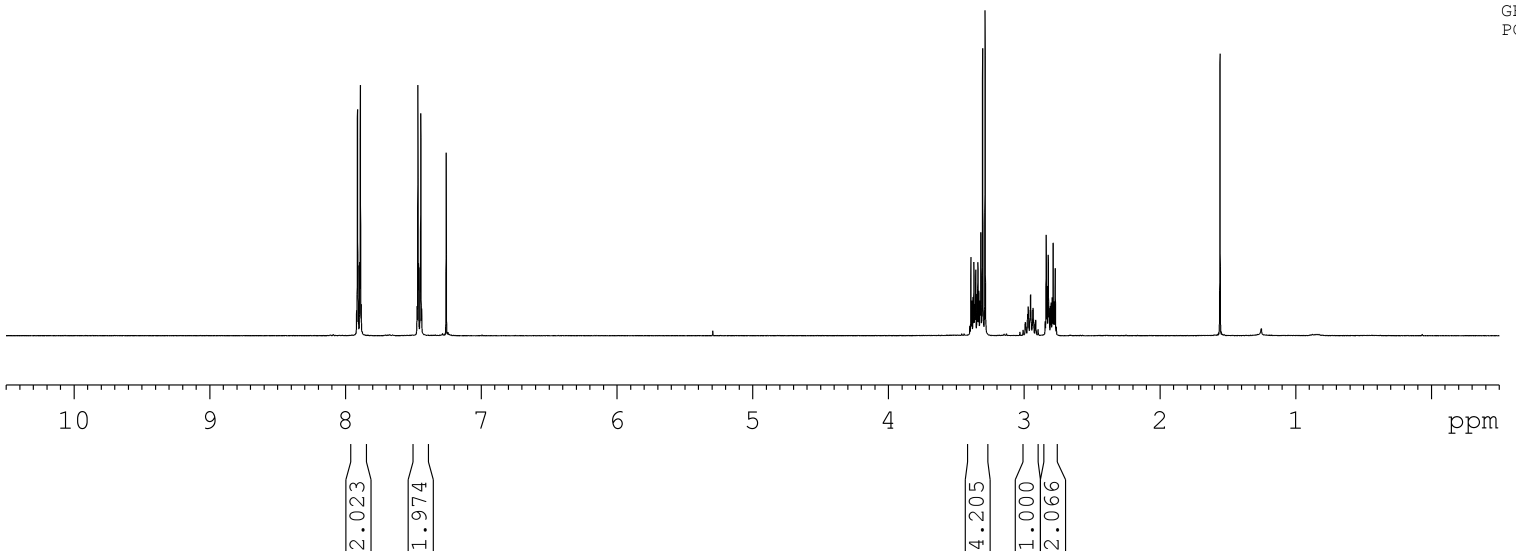

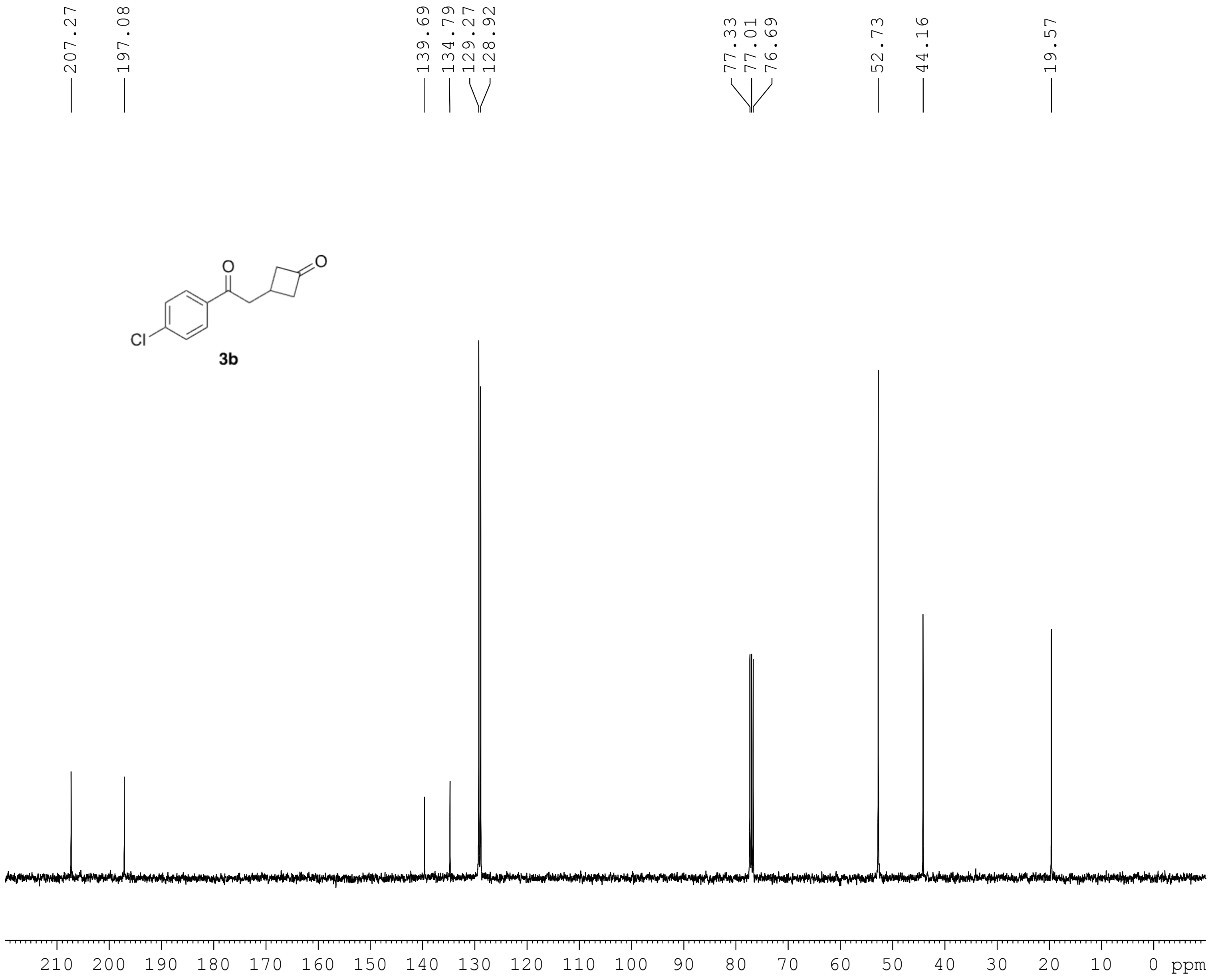


```
Current Data Parameters
NAME              K 4-Cl
EXPNO                  2
PROCNO                 1

F2 - Acquisition Parameters
Date_           20231012
Time               17.53 h
INSTRUM            spect
PROBHD   Z108618_0411 (
PULPROG          zgpg30
TD                 65536
SOLVENT            CDCl3
NS                    41
DS                     4
SWH            28409.092 Hz
FIDRES          0.866977 Hz
AQ             1.1534336 sec
RG                212.49
DW                17.600 usec
DE                  6.50 usec
TE                 298.9 K
D1            2.00000000 sec
D11           0.03000000 sec
TD0                    1
SFO1         100.6258487 MHz
NUC1                 13C
P1                 10.50 usec
PLW1         42.50000000 W
SFO2         400.1316005 MHz
NUC2                  1H
CPDPRG[2         waltz16
PCPD2              90.00 usec
PLW2          9.89999962 W
PLW12         0.29363999 W
PLW13         0.14747000 W

F2 - Processing parameters
SI                 32768
SF           100.6127792 MHz
WDW                   EM
SSB                    0
LB                  3.00 Hz
GB                     0
PC                  1.40
```

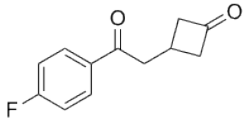

**3c**

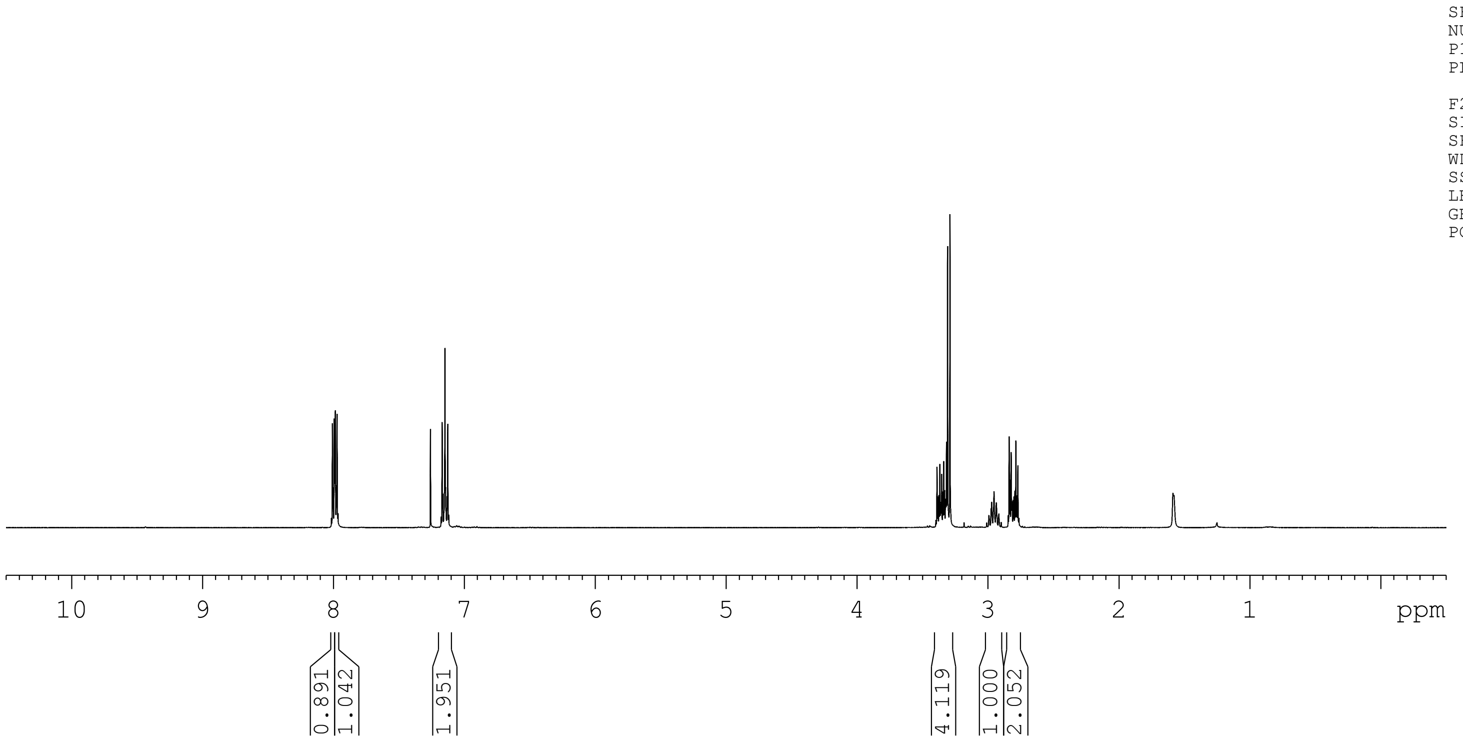


```
Current Data Parameters
NAME     20231123_oxidation_4F_specyra
EXPNO                    1
PROCNO                   1

F2 - Acquisition Parameters
Date_             20231123
Time                 15.35 h
INSTRUM              spect
PROBHD   Z108618_0411 (
PULPROG               zg30
TD                   32768
SOLVENT              CDCl3
NS                      21
DS                       0
SWH               8802.817 Hz
FIDRES            0.537281 Hz
AQ               1.8612224 sec
RG                  212.49
DW                  56.800 usec
DE                   14.47 usec
TE                   298.7 K
D1              1.00000000 sec
TD0                      1
SFO1           400.1328009 MHz
NUC1                    1H
P1                   15.50 usec
PLW1            9.89999962 W

F2 - Processing parameters
SI                  131072
SF             400.1300097 MHz
WDW                     EM
SSB                      0
LB                       0 Hz
GB                       0
PC                    1.00
```

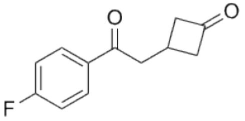

**3c**

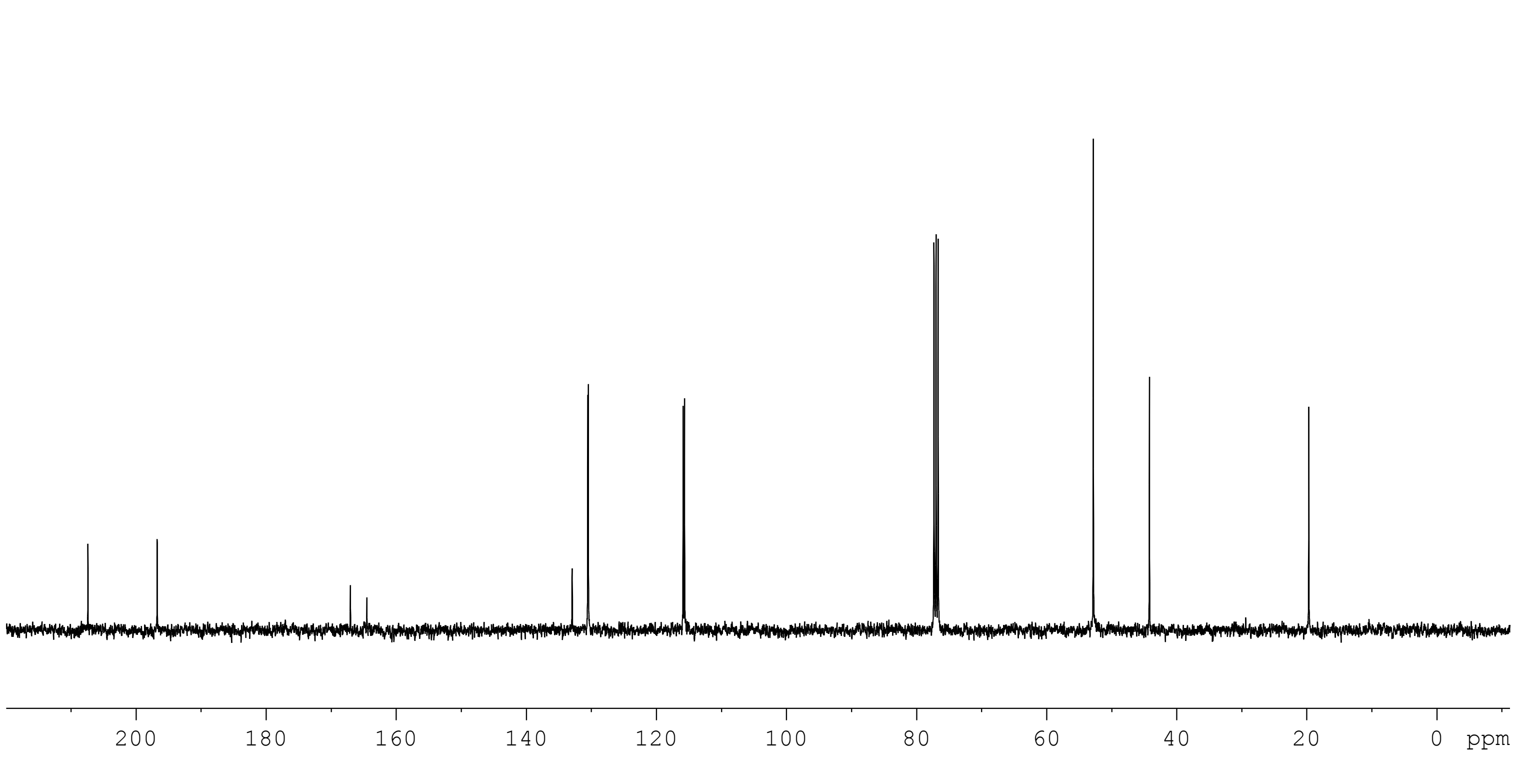


Current Data Parameters

| | |
|---|---|
| NAME | K 4F |
| EXPNO | 5 |
| PROCNO | 1 |

F2 - Acquisition Parameters

| | |
|---|---|
| Date_ | 20231124 |
| Time | 20.30 h |
| INSTRUM | spect |
| PROBHD | Z108618_0411 ( |
| PULPROG | zgpg30 |
| TD | 65536 |
| SOLVENT | CDCl3 |
| NS | 1 |
| DS | 4 |
| SWH | 28409.092 Hz |
| FIDRES | 0.866977 Hz |
| AQ | 1.1534336 sec |
| RG | 212.49 |
| DW | 17.600 usec |
| DE | 6.50 usec |
| TE | 298.7 K |
| D1 | 2.00000000 sec |
| D11 | 0.03000000 sec |
| TD0 | 1 |
| SFO1 | 100.6258487 MHz |
| NUC1 | 13C |
| P1 | 10.50 usec |
| PLW1 | 42.50000000 W |
| SFO2 | 400.1316005 MHz |
| NUC2 | 1H |
| CPDPRG[2 | waltz16 |
| PCPD2 | 90.00 usec |
| PLW2 | 9.89999962 W |
| PLW12 | 0.29363999 W |
| PLW13 | 0.14747000 W |

F2 - Processing parameters

| | |
|---|---|
| SI | 32768 |
| SF | 100.6127747 MHz |
| WDW | EM |
| SSB | 0 |
| LB | 3.00 Hz |
| GB | 0 |
| PC | 1.40 |

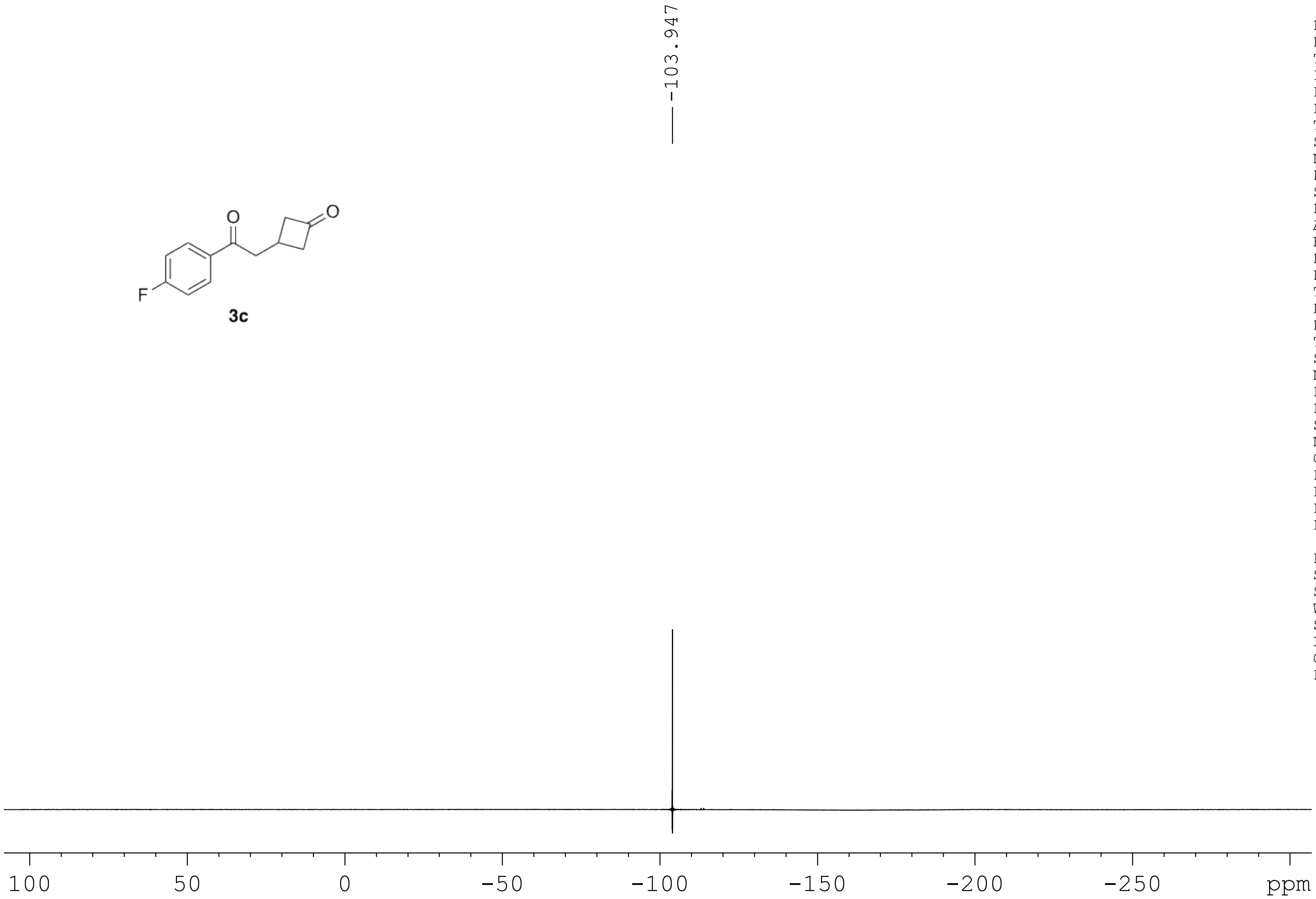


```
Current Data Parameters
NAME      20240808_K_4F
EXPNO                 1
PROCNO                1

F2 - Acquisition Parameters
Date_          20240808
Time              17.03 h
INSTRUM           spect
PROBHD   Z108618_0411 (
PULPROG         zgpg30
TD                65536
SOLVENT           CDCl3
NS                    6
DS                    2
SWH          156250.000 Hz
FIDRES         4.768372 Hz
AQ            0.2097152 sec
RG               212.49
DW                3.200 usec
DE                 6.92 usec
TE                298.1 K
D1           1.50000000 sec
D11          0.03000000 sec
TD0                   1
SFO1        376.4607162 MHz
NUC1                19F
P1                15.50 usec
PLW1        11.89999962 W
SFO2        400.1316005 MHz
NUC2                 1H
CPDPRG[2          garp4
PCPD2             90.00 usec
PLW2         9.89999962 W
PLW12        0.29363999 W
PLW13        0.14747000 W

F2 - Processing parameters
SI               131072
SF          376.4981150 MHz
WDW                  EM
SSB                   0
LB                    0 Hz
GB                    0
PC                 1.00
```

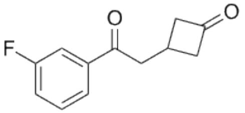

**3d**

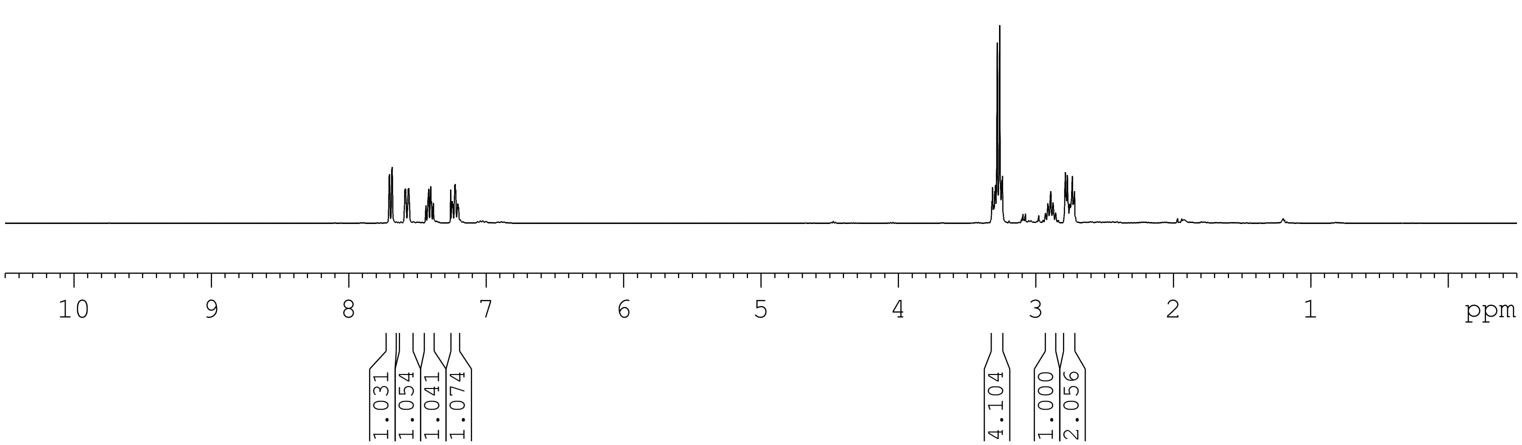


```
Current Data Parameters
NAME     20231127_oxidation_3F
EXPNO                    1
PROCNO                   1

F2 - Acquisition Parameters
Date_           20231127
Time               21.29 h
INSTRUM            spect
PROBHD   Z108618_0411 (
PULPROG             zg30
TD                 32768
SOLVENT            CDCl3
NS                    37
DS                     0
SWH             8802.817 Hz
FIDRES          0.537281 Hz
AQ             1.8612224 sec
RG                 31.38
DW                56.800 usec
DE                 14.47 usec
TE                 298.7 K
D1            1.00000000 sec
TD0                    1
SFO1         400.1328009 MHz
NUC1                  1H
P1                 15.50 usec
PLW1          9.89999962 W

F2 - Processing parameters
SI                131072
SF           400.1300092 MHz
WDW                   EM
SSB                    0
LB                     0 Hz
GB                     0
PC                  1.00
```

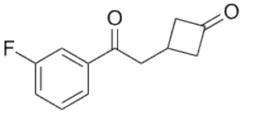


**3d**

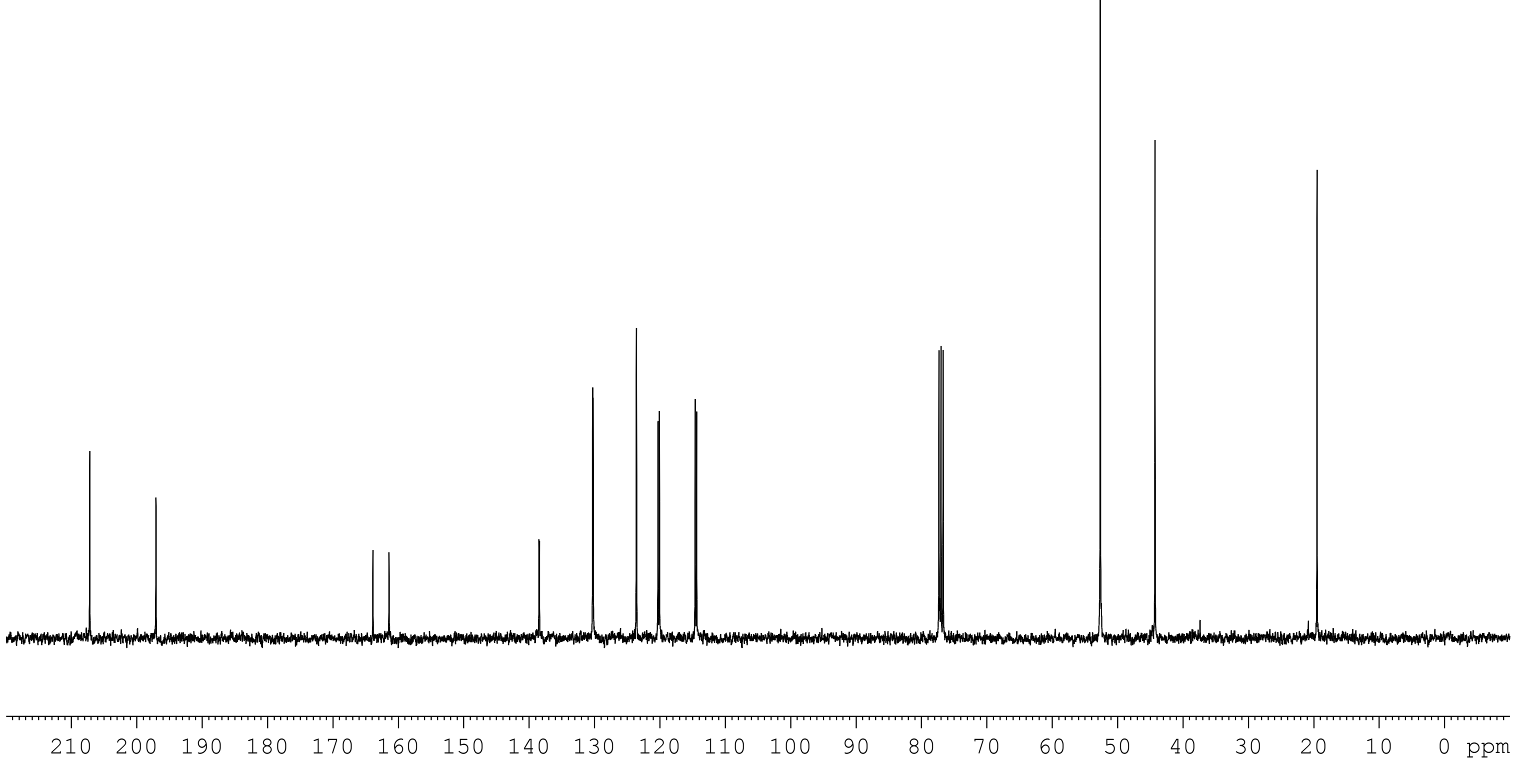


Current Data Parameters
NAME K 3F
EXPNO 2
PROCNO 1

F2 - Acquisition Parameters
Date_ 20231127
Time_ 21.32 h
INSTRUM spect
PROBHD Z108618_0411 (
PULPROG zgpg30
TD 65536
SOLVENT CDCl3
NS 39
DS 4
SWH 28409.092 Hz
FIDRES 0.866977 Hz
AQ 1.1534336 sec
RG 212.49
DW 17.600 usec
DE 6.50 usec
TE 298.8 K
D1 2.00000000 sec
D11 0.03000000 sec
TD0 1
SFO1 100.6258487 MHz
NUC1 13C
P1 10.50 usec
PLW1 42.50000000 W
SFO2 400.1316005 MHz
NUC2 1H
CPDPRG[2 waltz16
PCPD2 90.00 usec
PLW2 9.89999962 W
PLW12 0.29363999 W
PLW13 0.14747000 W

F2 - Processing parameters
SI 32768
SF 100.6127835 MHz
WDW EM
SSB 0
LB 3.00 Hz
GB 0
PC 1.40

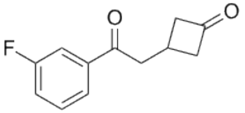

3d

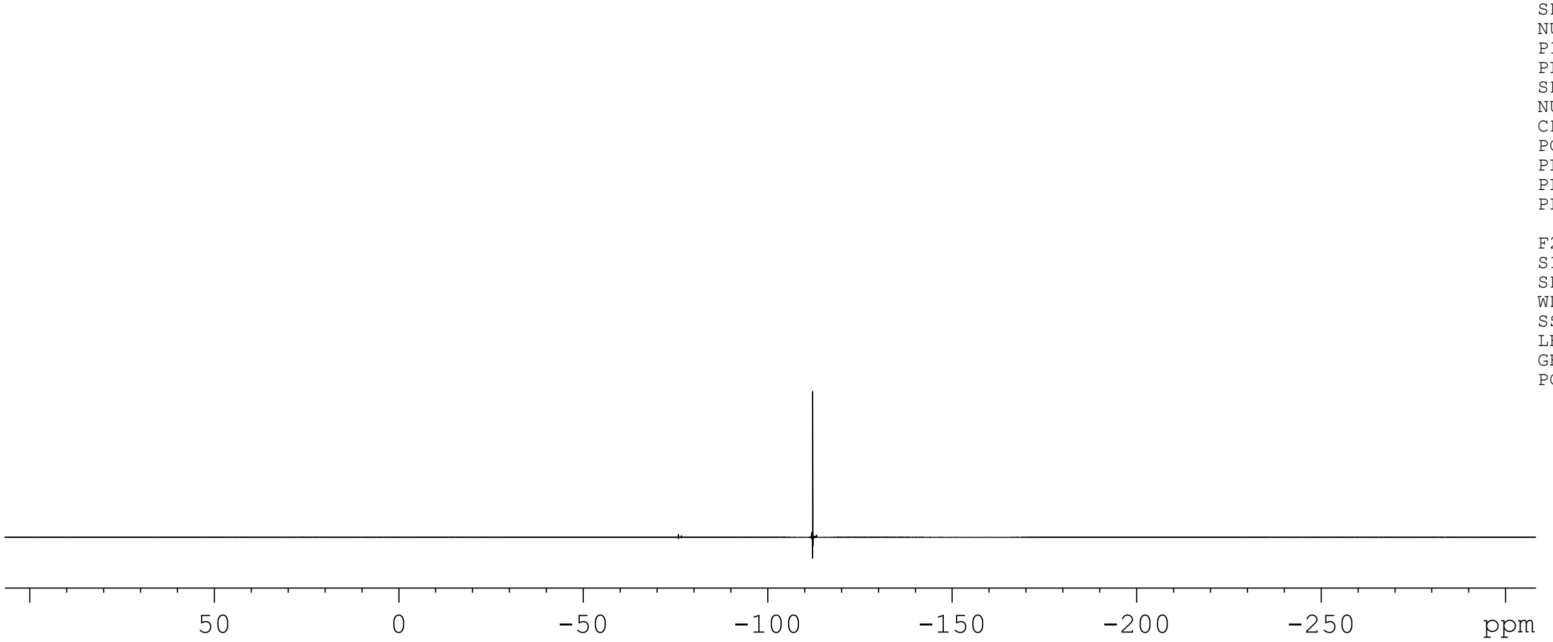


Current Data Parameters
NAME 20240808_K_3F
EXPNO 1
PROCNO 1

F2 - Acquisition Parameters
Date_ 20240808
Time 16.59 h
INSTRUM spect
PROBHD Z108618_0411 (
PULPROG zgpg30
TD 65536
SOLVENT CDCl3
NS 12
DS 2
SWH 156250.000 Hz
FIDRES 4.768372 Hz
AQ 0.2097152 sec
RG 212.49
DW 3.200 usec
DE 6.92 usec
TE 298.1 K
D1 1.50000000 sec
D11 0.03000000 sec
TD0 1
SFO1 376.4607162 MHz
NUC1 19F
P1 15.50 usec
PLW1 11.89999962 W
SFO2 400.1316005 MHz
NUC2 1H
CPDPRG[2 garp4
PCPD2 90.00 usec
PLW2 9.89999962 W
PLW12 0.29363999 W
PLW13 0.14747000 W

F2 - Processing parameters
SI 131072
SF 376.4986279 MHz
WDW EM
SSB 0
LB 0 Hz
GB 0
PC 1.00

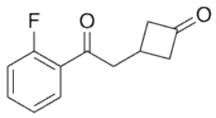

**3e**

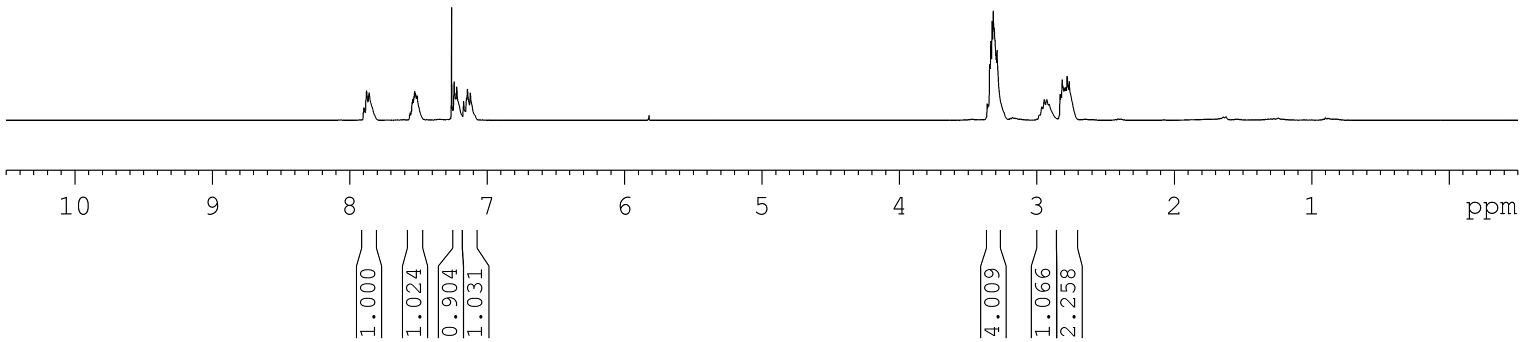


Current Data Parameters
NAME 20240229_oxidation_2F
EXPNO 1
PROCNO 1

F2 - Acquisition Parameters
Date_ 20240229
Time 15.27 h
INSTRUM spect
PROBHD Z108618_0411 (
PULPROG zg30
TD 32768
SOLVENT CDCl3
NS 17
DS 0
SWH 8802.817 Hz
FIDRES 0.537281 Hz
AQ 1.8612224 sec
RG 94.01
DW 56.800 usec
DE 14.47 usec
TE 298.0 K
D1 1.00000000 sec
TD0 1
SFO1 400.1328009 MHz
NUC1 1H
P1 15.50 usec
PLW1 9.89999962 W

F2 - Processing parameters
SI 131072
SF 400.1300096 MHz
WDW EM
SSB 0
LB 0 Hz
GB 0
PC 1.00

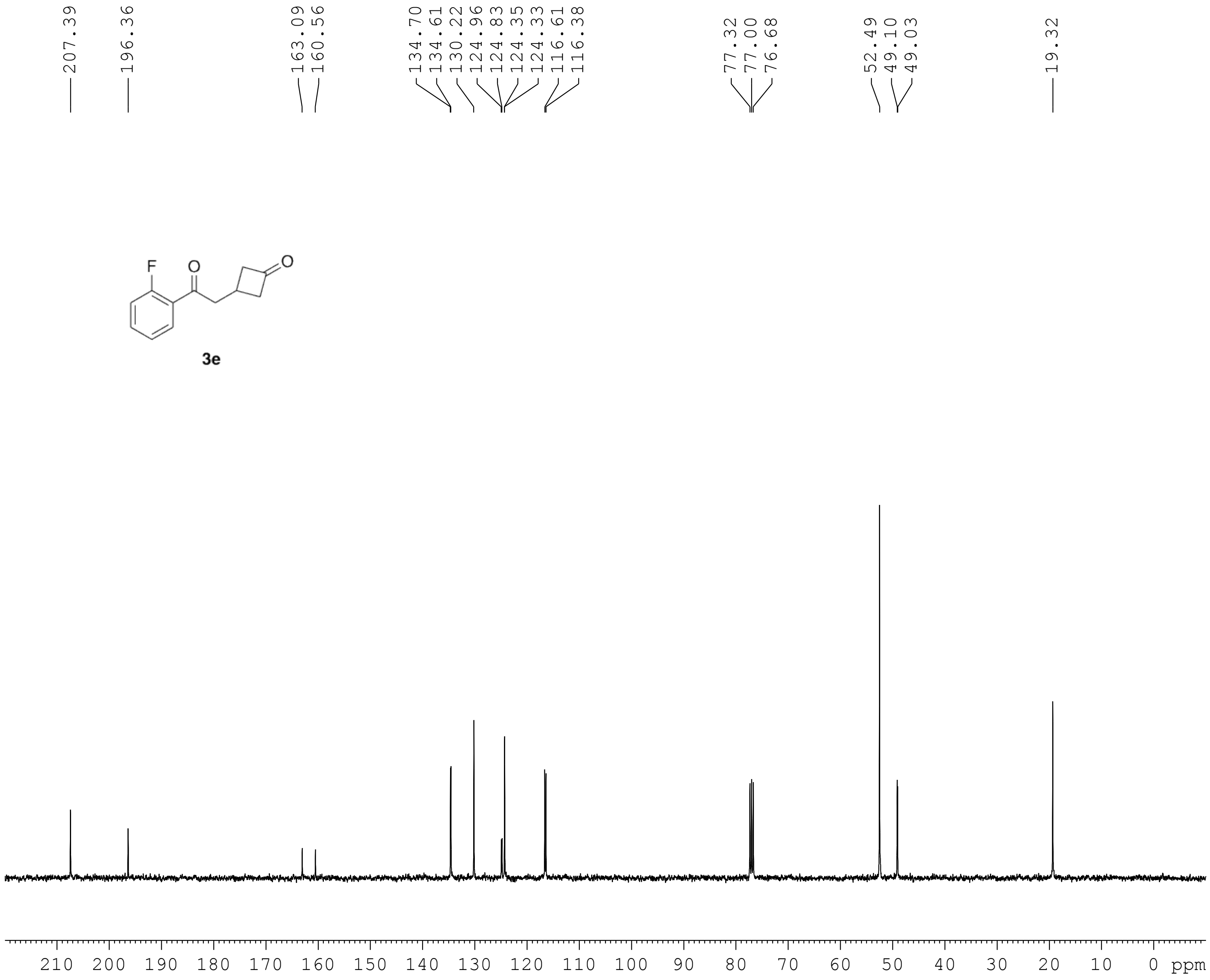


**3e**

```
Current Data Parameters
NAME                K 2F C
EXPNO                    5
PROCNO                   1

F2 - Acquisition Parameters
Date_             20240302
Time                 10.47 h
INSTRUM              spect
PROBHD   Z108618_0411 (
PULPROG            zgpg30
TD                   65536
SOLVENT              CDCl3
NS                      31
DS                       0
SWH              28409.092 Hz
FIDRES            0.866977 Hz
AQ               1.1534336 sec
RG                  212.49
DW                  17.600 usec
DE                    6.50 usec
TE                   298.1 K
D1              2.00000000 sec
D11             0.03000000 sec
TD0                      1
SFO1          100.6258487 MHz
NUC1                   13C
P1                   10.50 usec
PLW1           42.50000000 W
SFO2          400.1316005 MHz
NUC2                    1H
CPDPRG[2           waltz16
PCPD2                90.00 usec
PLW2            9.89999962 W
PLW12           0.29363999 W
PLW13           0.14747000 W

F2 - Processing parameters
SI                   32768
SF            100.6127904 MHz
WDW                     EM
SSB                      0
LB                    3.00 Hz
GB                       0
PC                    1.40
```

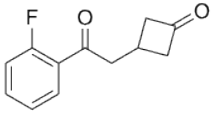


**3e**

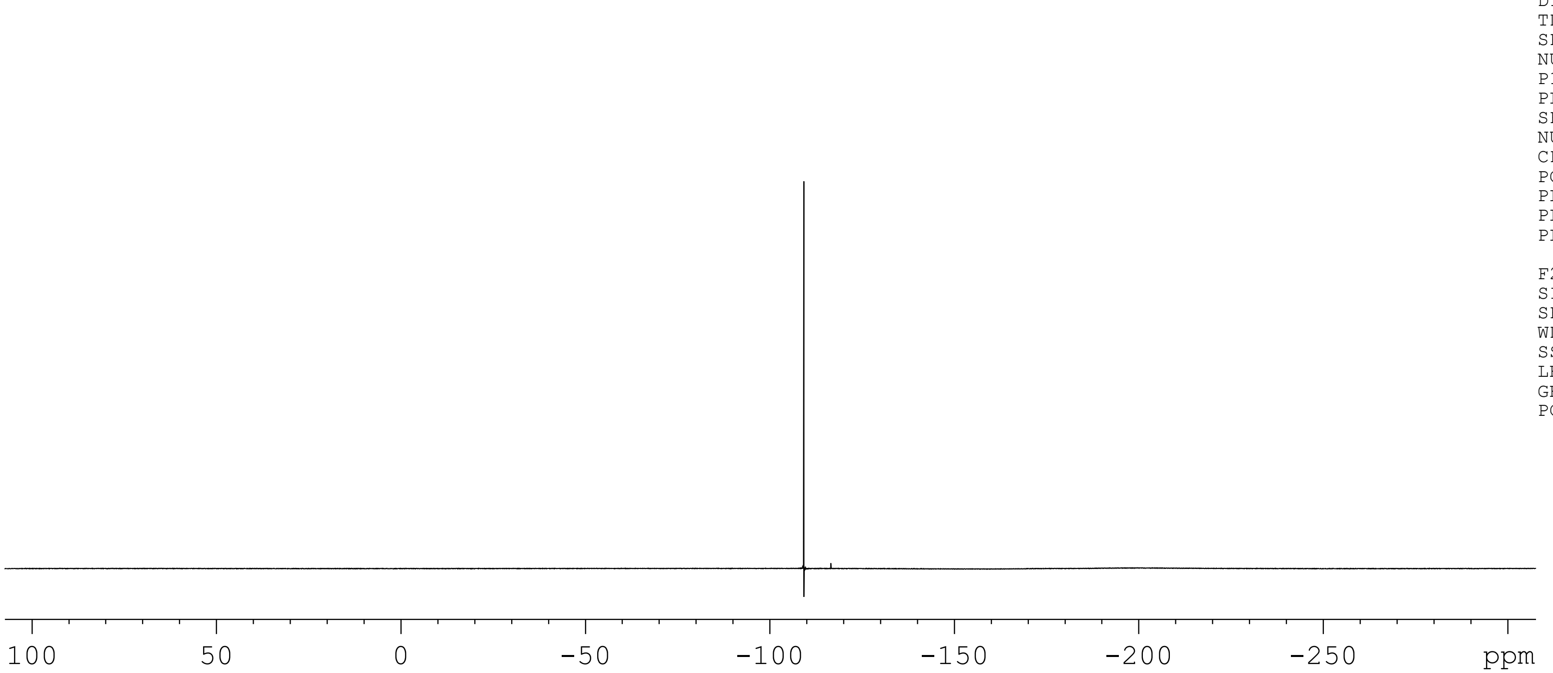


Current Data Parameters
NAME 20240808_K_2F
EXPNO 1
PROCNO 1

F2 - Acquisition Parameters
Date_ 20240808
Time 16.56 h
INSTRUM spect
PROBHD Z108618_0411 (
PULPROG zgpg30
TD 65536
SOLVENT CDCl3
NS 11
DS 2
SWH 156250.000 Hz
FIDRES 4.768372 Hz
AQ 0.2097152 sec
RG 212.49
DW 3.200 usec
DE 6.92 usec
TE 298.1 K
D1 1.50000000 sec
D11 0.03000000 sec
TD0 1
SFO1 376.4607162 MHz
NUC1 19F
P1 15.50 usec
PLW1 11.89999962 W
SFO2 400.1316005 MHz
NUC2 1H
CPDPRG[2 garp4
PCPD2 90.00 usec
PLW2 9.89999962 W
PLW12 0.29363999 W
PLW13 0.14747000 W

F2 - Processing parameters
SI 131072
SF 376.4984080 MHz
WDW EM
SSB 0
LB 0 Hz
GB 0
PC 1.00

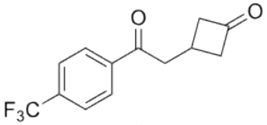

3f

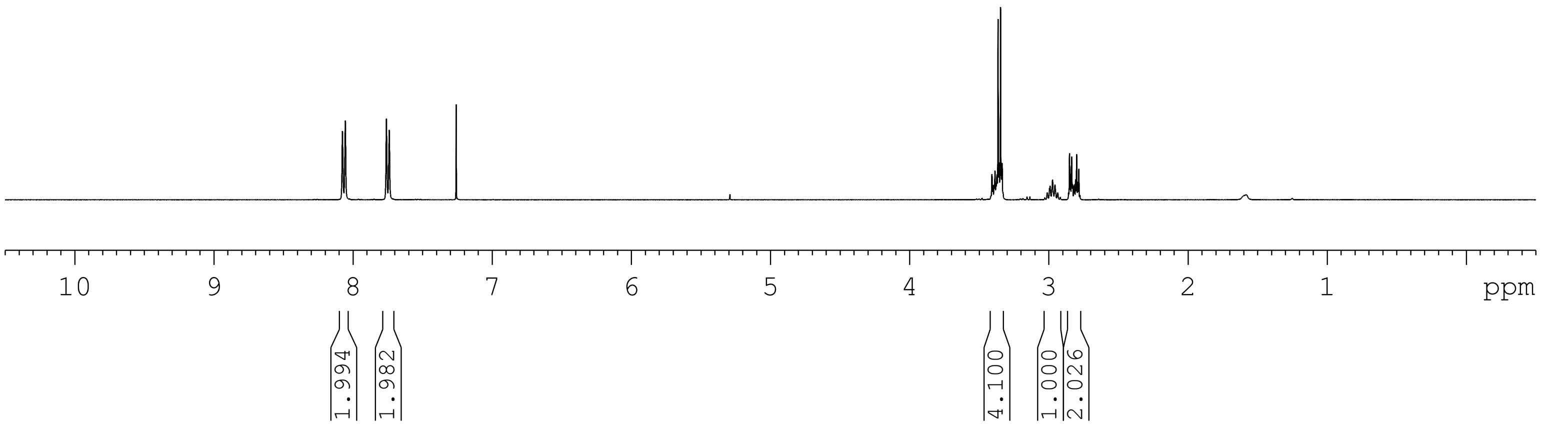


Current Data Parameters
NAME K 4CF3
EXPNO 2
PROCNO 1

F2 - Acquisition Parameters
Date_ 20240129
Time 17.53 h
INSTRUM spect
PROBHD Z108618_0411 (
PULPROG zg30
TD 32768
SOLVENT CDCl3
NS 13
DS 0
SWH 8802.817 Hz
FIDRES 0.537281 Hz
AQ 1.8612224 sec
RG 188.2
DW 56.800 usec
DE 14.47 usec
TE 298.0 K
D1 1.00000000 sec
TD0 1
SFO1 400.1328009 MHz
NUC1 1H
P1 15.50 usec
PLW1 9.89999962 W

F2 - Processing parameters
SI 131072
SF 400.1300096 MHz
WDW EM
SSB 0
LB 0 Hz
GB 0
PC 1.00

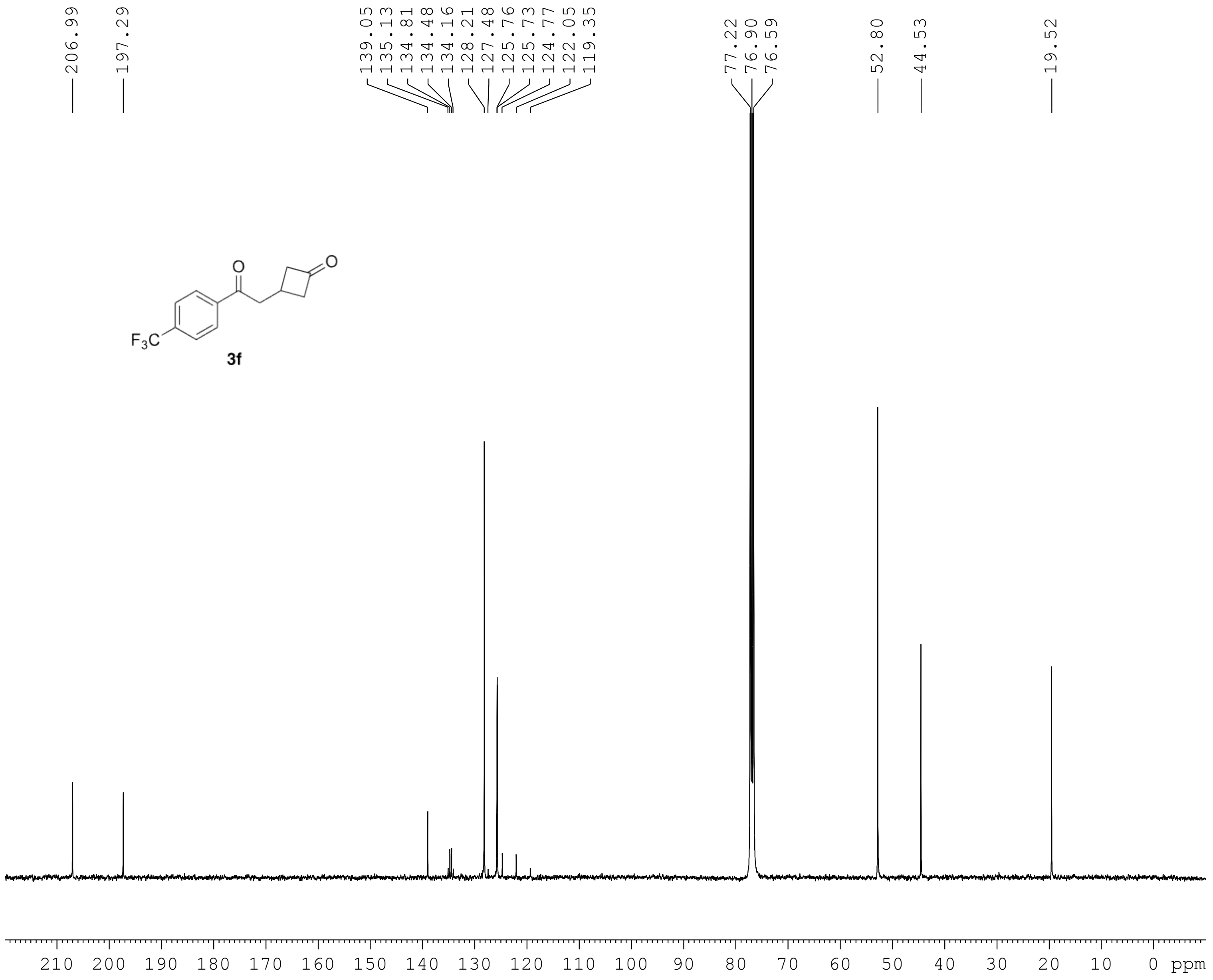


```
Current Data Parameters
NAME        20240806_K_CF3
EXPNO                    1
PROCNO                   1

F2 - Acquisition Parameters
Date_             20240807
Time                  8.52 h
INSTRUM              spect
PROBHD   Z108618_0411 (
PULPROG            zgpg30
TD                   65536
SOLVENT              CDCl3
NS                   12030
DS                       4
SWH              28409.092 Hz
FIDRES            0.866977 Hz
AQ               1.1534336 sec
RG                  212.49
DW                  17.600 usec
DE                    6.50 usec
TE                   298.1 K
D1              2.00000000 sec
D11             0.03000000 sec
TD0                      1
SFO1           100.6258487 MHz
NUC1                   13C
P1                   10.50 usec
PLW1           42.50000000 W
SFO2           400.1316005 MHz
NUC2                    1H
CPDPRG[2           waltz16
PCPD2                90.00 usec
PLW2            9.89999962 W
PLW12           0.29363999 W
PLW13           0.14747000 W

F2 - Processing parameters
SI                   32768
SF             100.6127792 MHz
WDW                     EM
SSB                      0
LB                    3.00 Hz
GB                       0
PC                    1.40
```

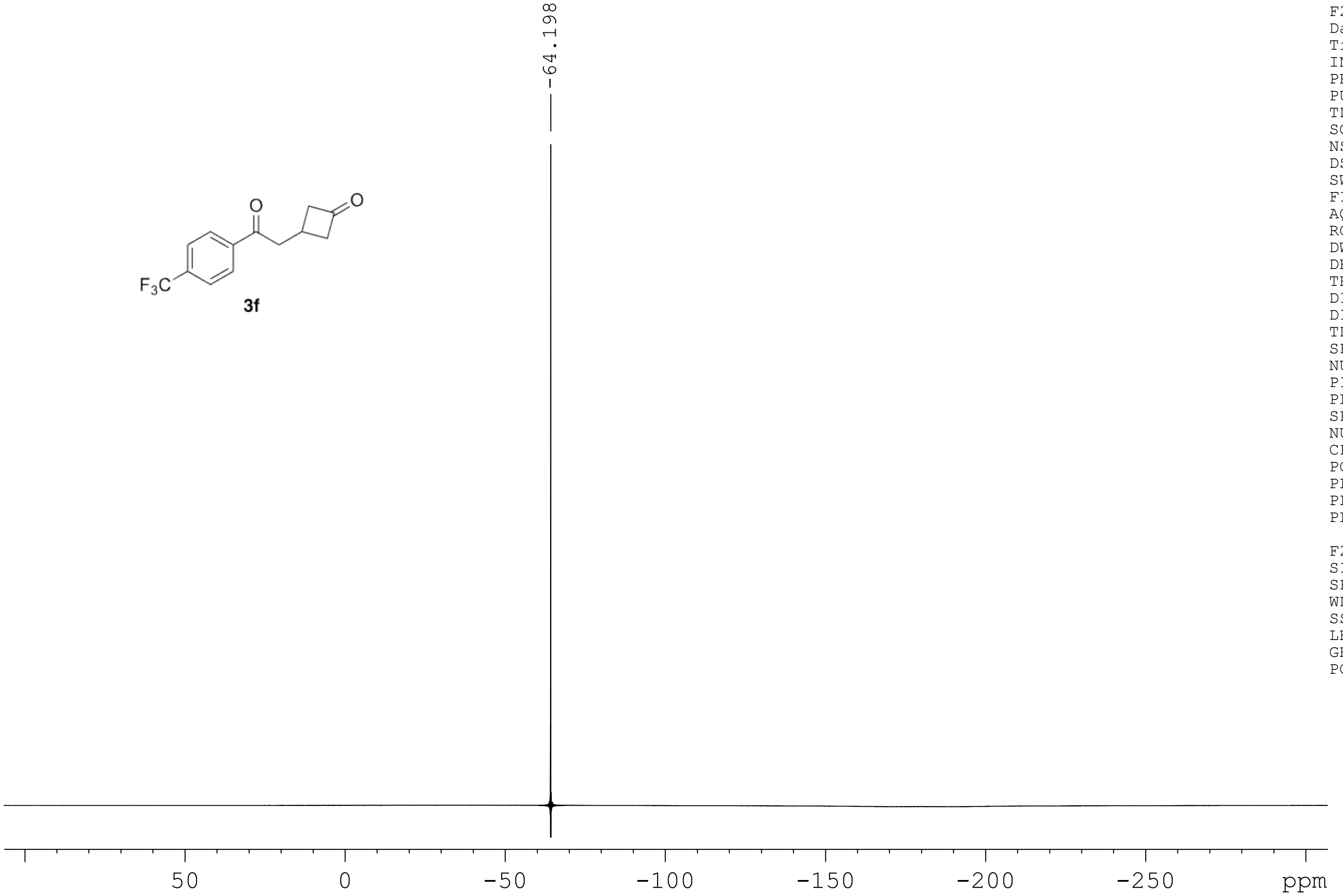


```
Current Data Parameters
NAME      20240808_K_CF3
EXPNO                  1
PROCNO                 1

F2 - Acquisition Parameters
Date_           20240809
Time               10.58 h
INSTRUM            spect
PROBHD    Z108618_0411 (
PULPROG          zgpg30
TD                 65536
SOLVENT            CDCl3
NS                    24
DS                     2
SWH           156250.000 Hz
FIDRES          4.768372 Hz
AQ             0.2097152 sec
RG                212.49
DW                 3.200 usec
DE                  6.92 usec
TE                 298.0 K
D1            1.50000000 sec
D11           0.03000000 sec
TD0                    1
SFO1         376.4607162 MHz
NUC1                 19F
P1                 15.50 usec
PLW1         11.89999962 W
SFO2         400.1316005 MHz
NUC2                  1H
CPDPRG[2           garp4
PCPD2              90.00 usec
PLW2          9.89999962 W
PLW12         0.29363999 W
PLW13         0.14747000 W

F2 - Processing parameters
SI                131072
SF           376.4987474 MHz
WDW                   EM
SSB                    0
LB                     0 Hz
GB                     0
PC                  1.00
```

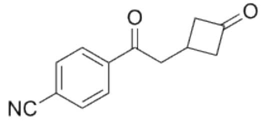


3g

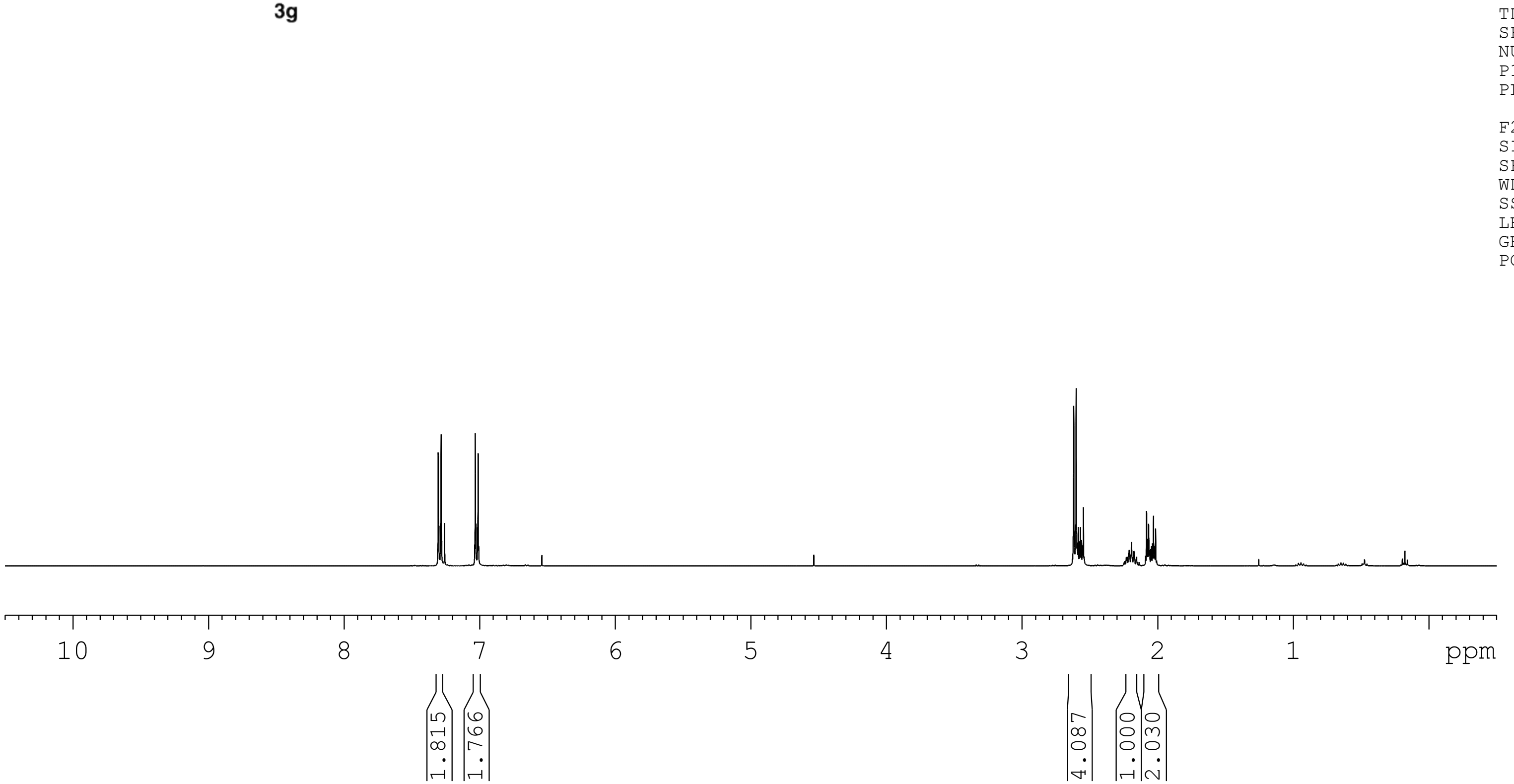


```
Current Data Parameters
NAME     20240320_oxidation_4CN
EXPNO                  3
PROCNO                 1

F2 - Acquisition Parameters
Date_           20240321
Time               17.35 h
INSTRUM            spect
PROBHD   Z108618_0411 (
PULPROG             zg30
TD                 32768
SOLVENT            CDCl3
NS                    28
DS                     0
SWH             8802.817 Hz
FIDRES          0.537281 Hz
AQ             1.8612224 sec
RG                 31.38
DW                56.800 usec
DE                 14.47 usec
TE                 298.0 K
D1            1.00000000 sec
TD0                    1
SFO1         400.1328009 MHz
NUC1                  1H
P1                 15.50 usec
PLW1          9.89999962 W

F2 - Processing parameters
SI                131072
SF           400.1302963 MHz
WDW                   EM
SSB                    0
LB                     0 Hz
GB                     0
PC                  1.00
```

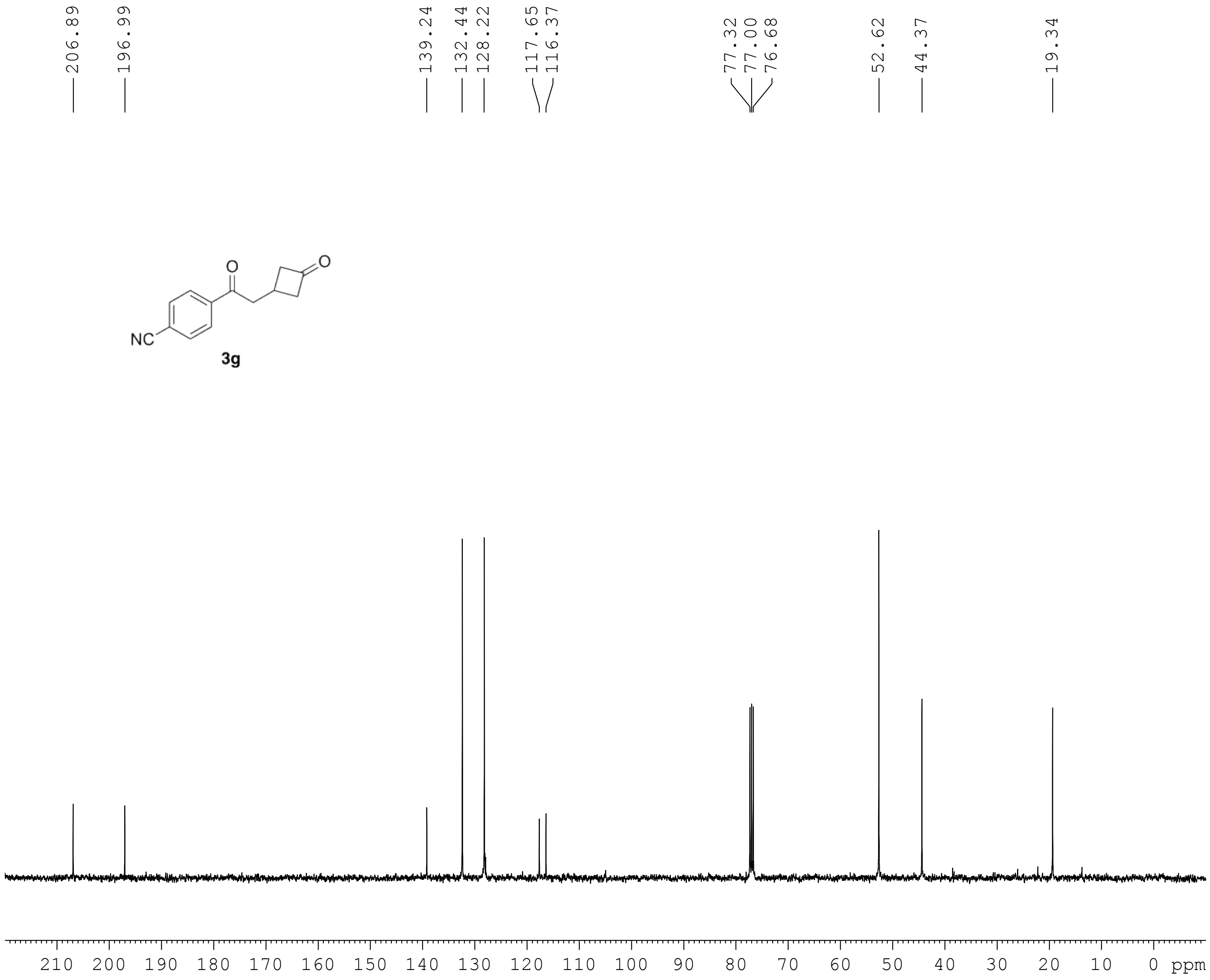


**3g**

```
Current Data Parameters
NAME                K 4CN
EXPNO                   4
PROCNO                  1

F2 - Acquisition Parameters
Date_            20240321
Time                17.39 h
INSTRUM             spect
PROBHD   Z108618_0411 (
PULPROG           zgpg30
TD                 65536
SOLVENT            CDCl3
NS                    23
DS                     4
SWH            28409.092 Hz
FIDRES          0.866977 Hz
AQ             1.1534336 sec
RG                212.49
DW                17.600 usec
DE                  6.50 usec
TE                 298.1 K
D1            2.00000000 sec
D11           0.03000000 sec
TD0                    1
SFO1         100.6258487 MHz
NUC1                 13C
P1                 10.50 usec
PLW1         42.50000000 W
SFO2         400.1316005 MHz
NUC2                  1H
CPDPRG[2         waltz16
PCPD2              90.00 usec
PLW2          9.89999962 W
PLW12         0.29363999 W
PLW13         0.14747000 W

F2 - Processing parameters
SI                 32768
SF           100.6127869 MHz
WDW                   EM
SSB                    0
LB                  3.00 Hz
GB                     0
PC                  1.40
```

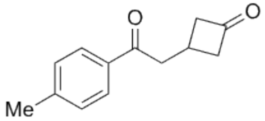


**3h**

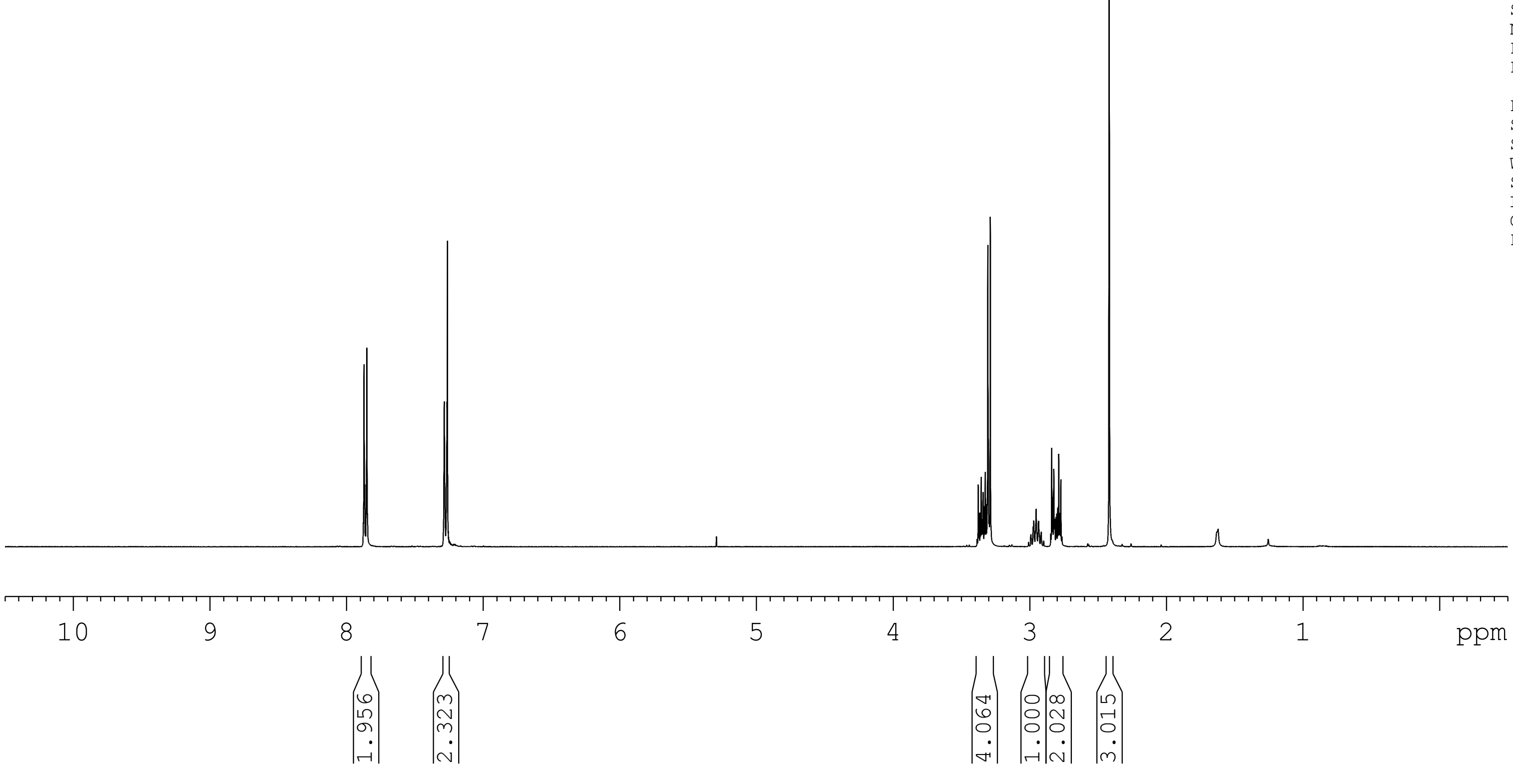


```
Current Data Parameters
NAME     20240219_oxidation_4Me
EXPNO                    1
PROCNO                   1

F2 - Acquisition Parameters
Date_            20240219
Time                17.34 h
INSTRUM             spect
PROBHD   Z108618_0411 (
PULPROG              zg30
TD                  32768
SOLVENT             CDCl3
NS                     20
DS                      0
SWH              8802.817 Hz
FIDRES           0.537281 Hz
AQ              1.8612224 sec
RG                 212.49
DW                 56.800 usec
DE                  14.47 usec
TE                  298.0 K
D1             1.00000000 sec
TD0                     1
SFO1          400.1328009 MHz
NUC1                   1H
P1                  15.50 usec
PLW1           9.89999962 W

F2 - Processing parameters
SI                 131072
SF            400.1300094 MHz
WDW                    EM
SSB                     0
LB                      0 Hz
GB                      0
PC                   1.00
```

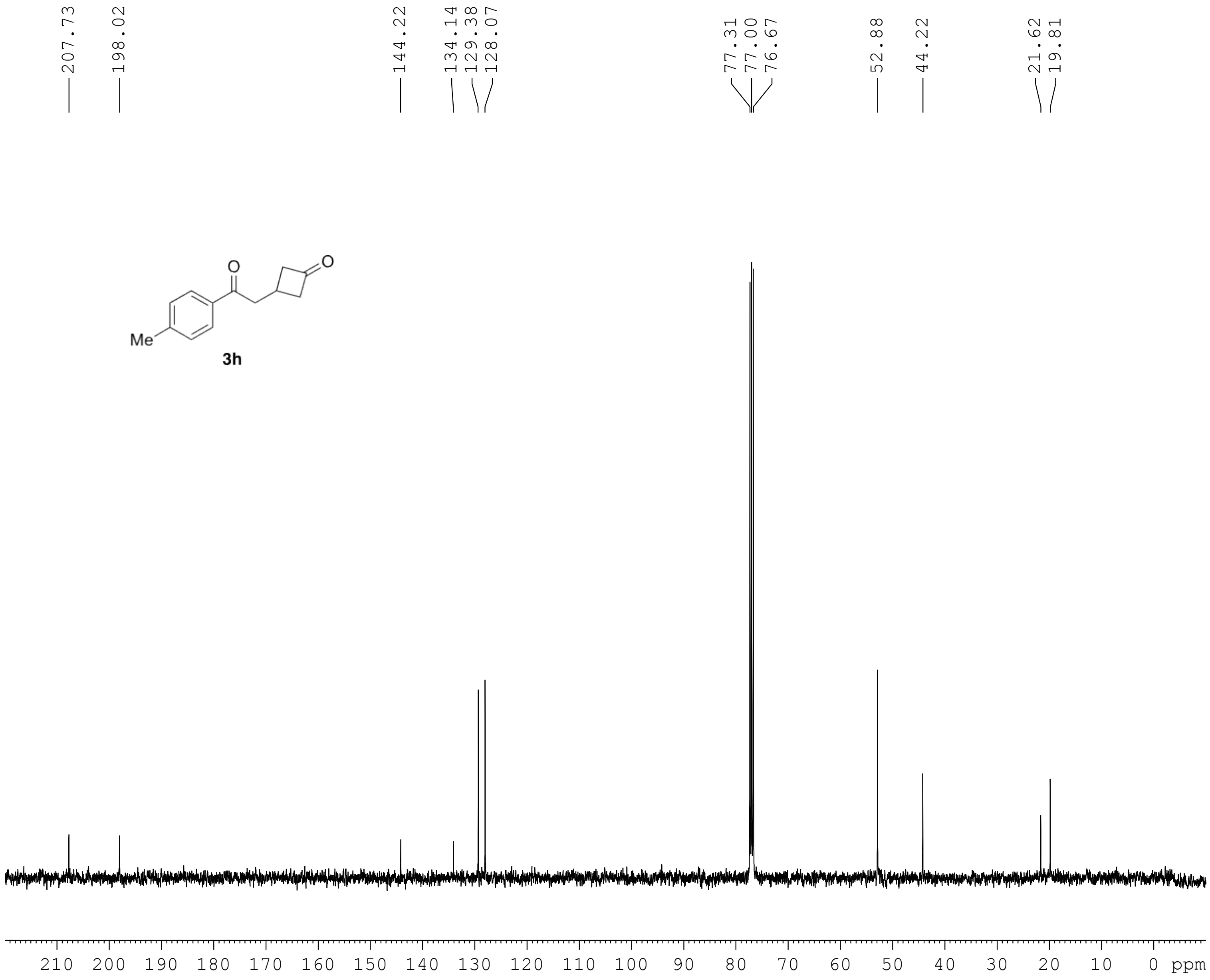


```
Current Data Parameters
NAME              K 4Me
EXPNO                 2
PROCNO                1

F2 - Acquisition Parameters
Date_          20240219
Time              17.42 h
INSTRUM           spect
PROBHD   Z108618_0411 (
PULPROG         zgpg30
TD               65536
SOLVENT          CDCl3
NS                 102
DS                   4
SWH          28409.092 Hz
FIDRES        0.866977 Hz
AQ           1.1534336 sec
RG              212.49
DW              17.600 usec
DE                6.50 usec
TE               298.1 K
D1          2.00000000 sec
D11         0.03000000 sec
TD0                  1
SFO1       100.6258487 MHz
NUC1               13C
P1               10.50 usec
PLW1       42.50000000 W
SFO2       400.1316005 MHz
NUC2                1H
CPDPRG[2       waltz16
PCPD2            90.00 usec
PLW2        9.89999962 W
PLW12       0.29363999 W
PLW13       0.14747000 W

F2 - Processing parameters
SI               32768
SF         100.6127718 MHz
WDW                 EM
SSB                  0
LB                3.00 Hz
GB                   0
PC                1.40
```

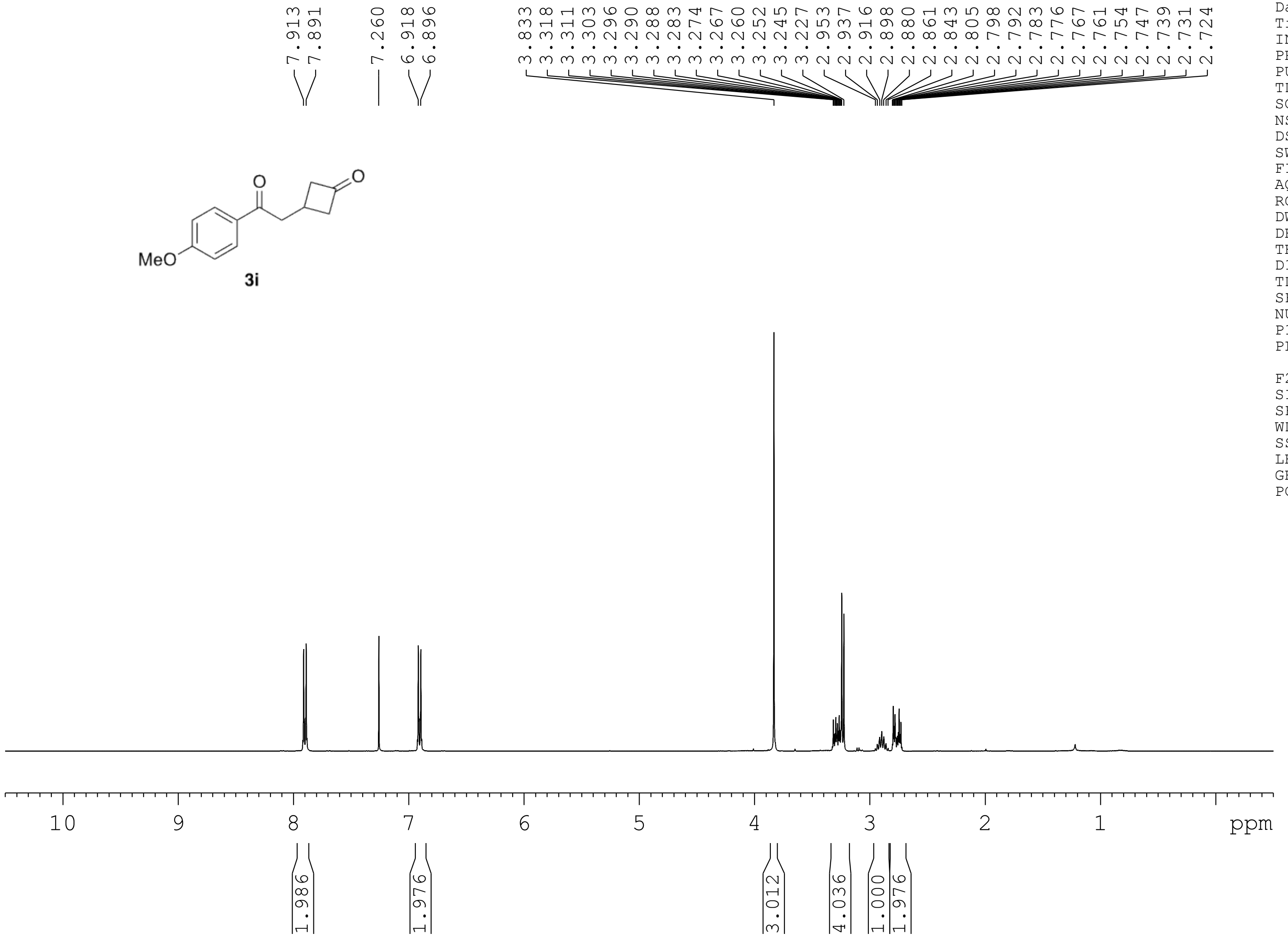


```
Current Data Parameters
NAME     20231225_oxidation_4OMe
EXPNO                     1
PROCNO                    1

F2 - Acquisition Parameters
Date_            20231225
Time                13.17 h
INSTRUM             spect
PROBHD   Z108618_0411 (
PULPROG              zg30
TD                  32768
SOLVENT             CDCl3
NS                     15
DS                      0
SWH              8802.817 Hz
FIDRES           0.537281 Hz
AQ              1.8612224 sec
RG                  51.61
DW                 56.800 usec
DE                  14.47 usec
TE                  298.0 K
D1             1.00000000 sec
TD0                     1
SFO1          400.1328009 MHz
NUC1                   1H
P1                  15.50 usec
PLW1           9.89999962 W

F2 - Processing parameters
SI                 131072
SF            400.1300092 MHz
WDW                    EM
SSB                     0
LB                      0 Hz
GB                      0
PC                   1.00
```

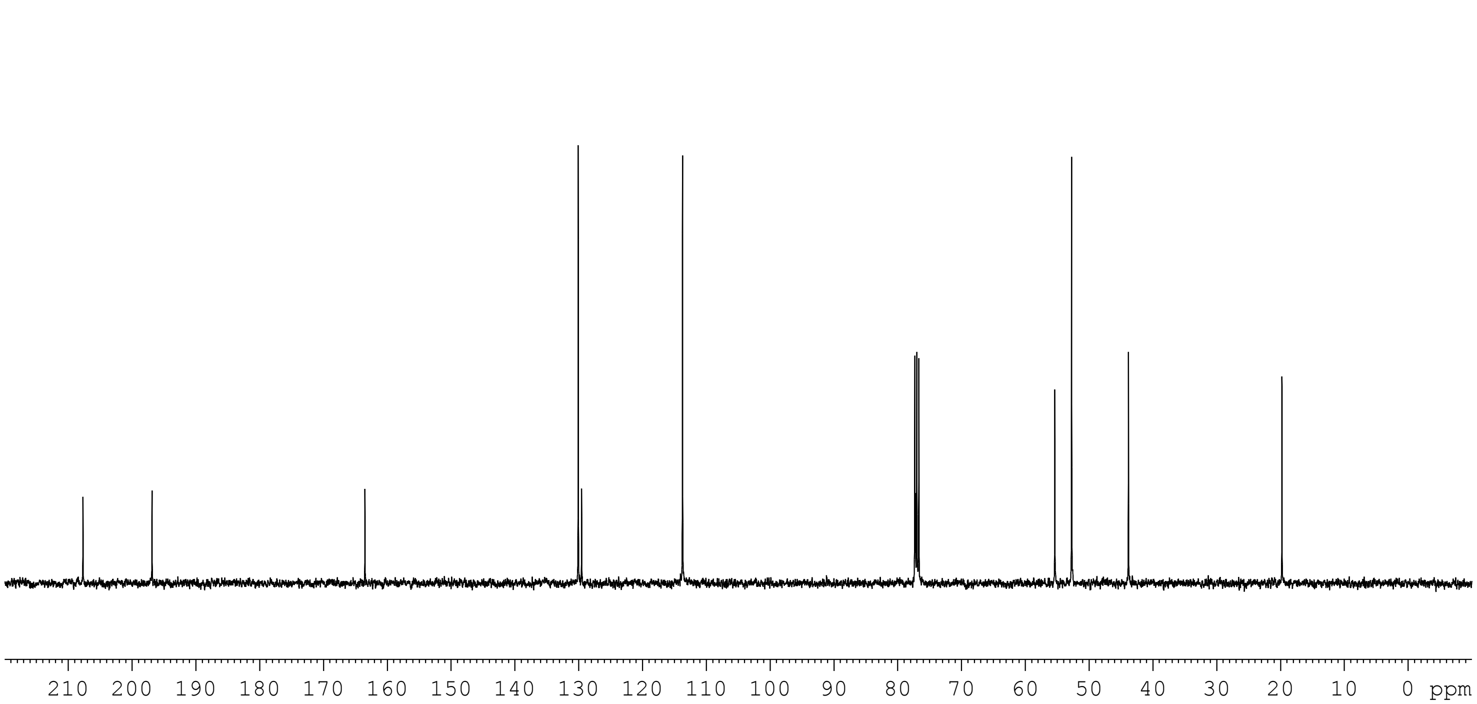


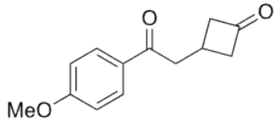

**3i**

Current Data Parameters
NAME 20231225_oxidation_4OMe
EXPNO 2
PROCNO 1

F2 - Acquisition Parameters
Date_ 20231225
Time 13.20 h
INSTRUM spect
PROBHD Z108618_0411 (
PULPROG zgpg30
TD 65536
SOLVENT CDCl3
NS 27
DS 4
SWH 28409.092 Hz
FIDRES 0.866977 Hz
AQ 1.1534336 sec
RG 212.49
DW 17.600 usec
DE 6.50 usec
TE 298.1 K
D1 2.00000000 sec
D11 0.03000000 sec
TD0 1
SFO1 100.6258487 MHz
NUC1 13C
P1 10.50 usec
PLW1 42.50000000 W
SFO2 400.1316005 MHz
NUC2 1H
CPDPRG[2 waltz16
PCPD2 90.00 usec
PLW2 9.89999962 W
PLW12 0.29363999 W
PLW13 0.14747000 W

F2 - Processing parameters
SI 32768
SF 100.6127817 MHz
WDW EM
SSB 0
LB 3.00 Hz
GB 0
PC 1.40

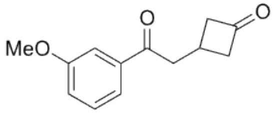


**3j**

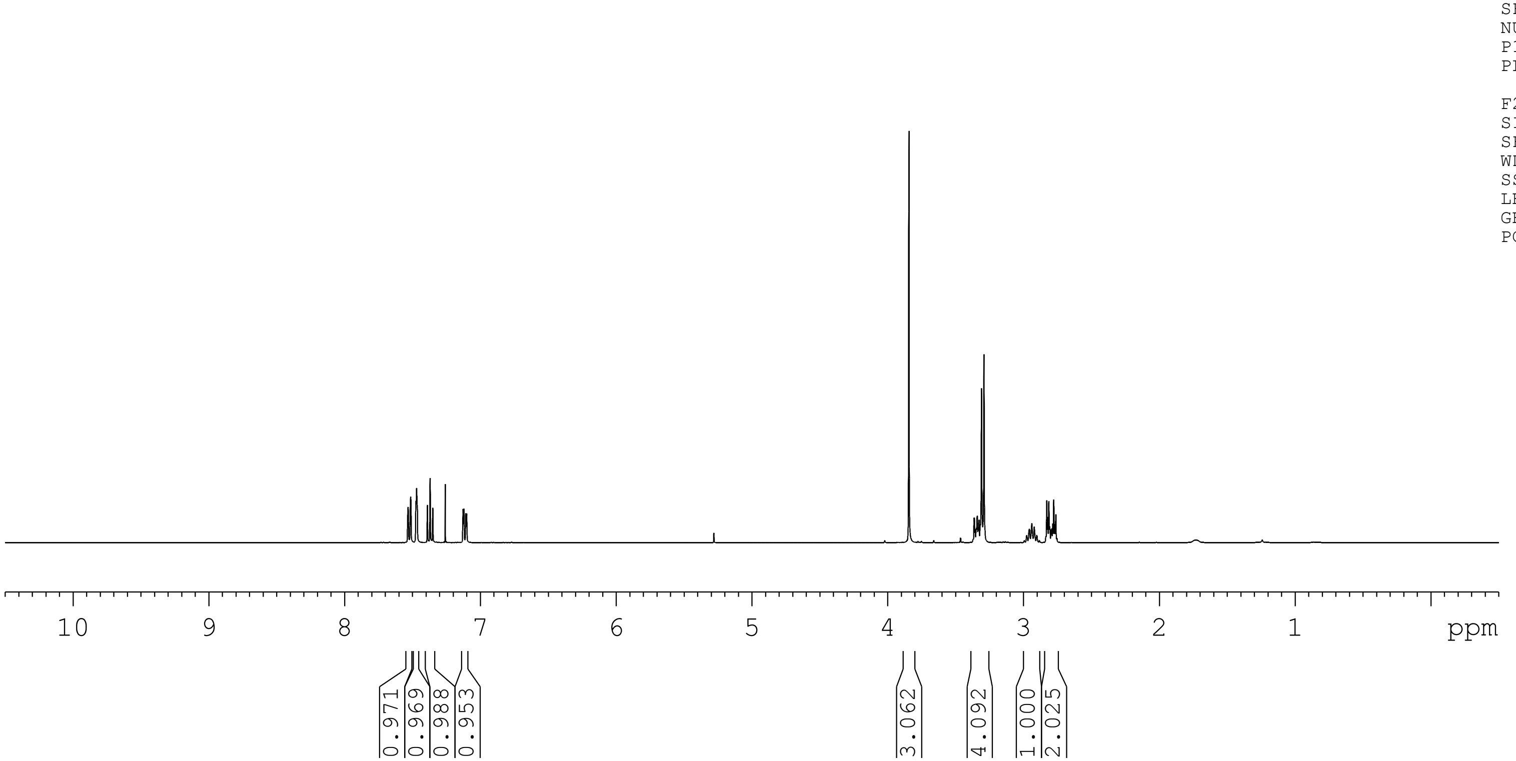


```
Current Data Parameters
NAME     20240129_oxidation_3OMe
EXPNO                    4
PROCNO                   1

F2 - Acquisition Parameters
Date_             20240131
Time                 11.12 h
INSTRUM              spect
PROBHD    Z108618_0411 (
PULPROG               zg30
TD                   32768
SOLVENT              CDCl3
NS                      27
DS                       0
SWH               8802.817 Hz
FIDRES            0.537281 Hz
AQ               1.8612224 sec
RG                  103.36
DW                  56.800 usec
DE                   14.47 usec
TE                   298.0 K
D1              1.00000000 sec
TD0                      1
SFO1           400.1328009 MHz
NUC1                    1H
P1                   15.50 usec
PLW1            9.89999962 W

F2 - Processing parameters
SI                  131072
SF             400.1300096 MHz
WDW                     EM
SSB                      0
LB                       0 Hz
GB                       0
PC                    1.00
```

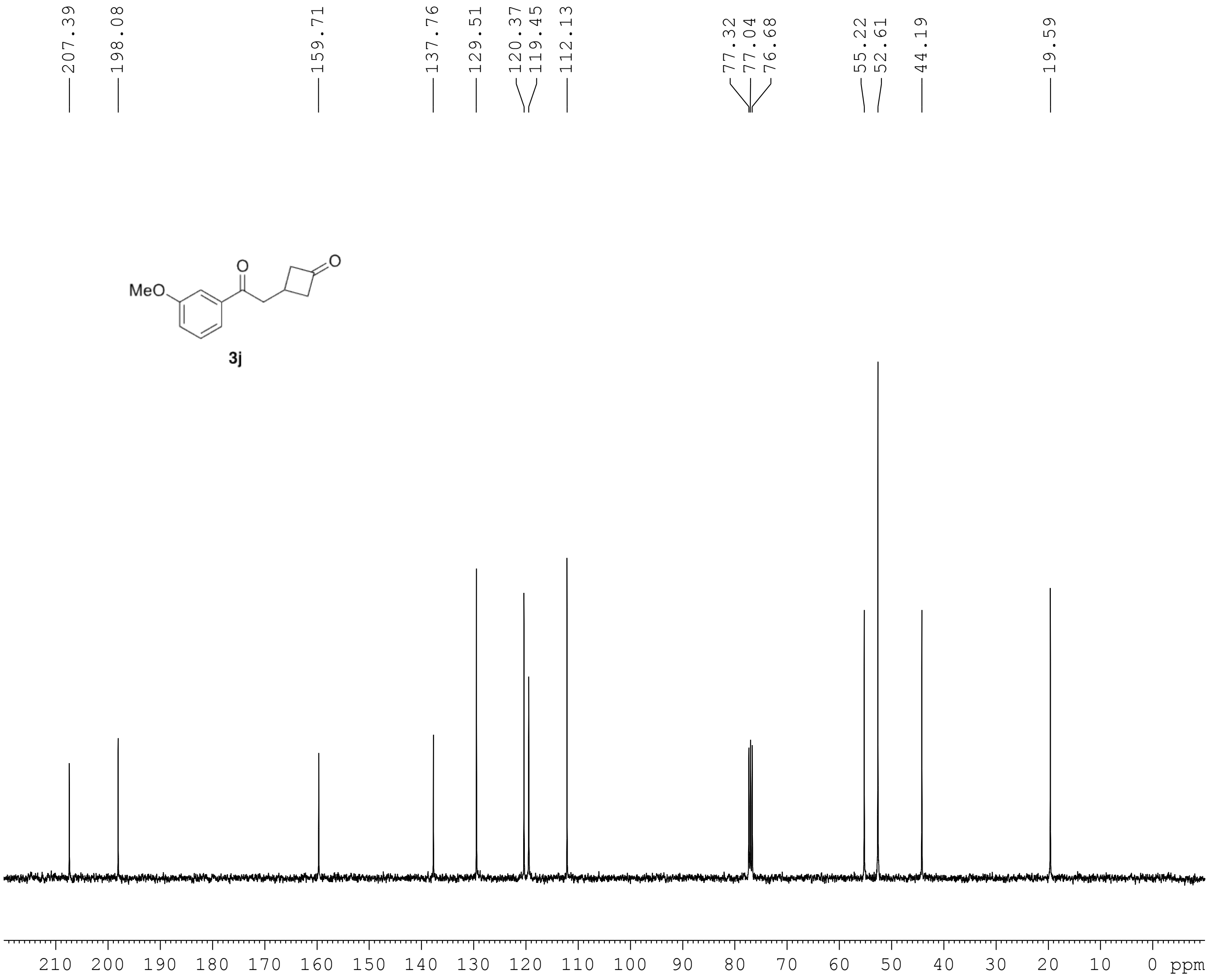


```
Current Data Parameters
NAME              K 3OMe
EXPNO                  3
PROCNO                 1

F2 - Acquisition Parameters
Date_           20240129
Time               17.50 h
INSTRUM            spect
PROBHD   Z108618_0411 (
PULPROG          zgpg30
TD                 65536
SOLVENT            CDCl3
NS                    33
DS                     4
SWH            28409.092 Hz
FIDRES          0.866977 Hz
AQ             1.1534336 sec
RG                212.49
DW                17.600 usec
DE                  6.50 usec
TE                 298.1 K
D1            2.00000000 sec
D11           0.03000000 sec
TD0                    1
SFO1         100.6258487 MHz
NUC1                 13C
P1                 10.50 usec
PLW1         42.50000000 W
SFO2         400.1316005 MHz
NUC2                  1H
CPDPRG[2         waltz16
PCPD2              90.00 usec
PLW2          9.89999962 W
PLW12         0.29363999 W
PLW13         0.14747000 W

F2 - Processing parameters
SI                 32768
SF           100.6127874 MHz
WDW                   EM
SSB                    0
LB                  3.00 Hz
GB                     0
PC                  1.40
```

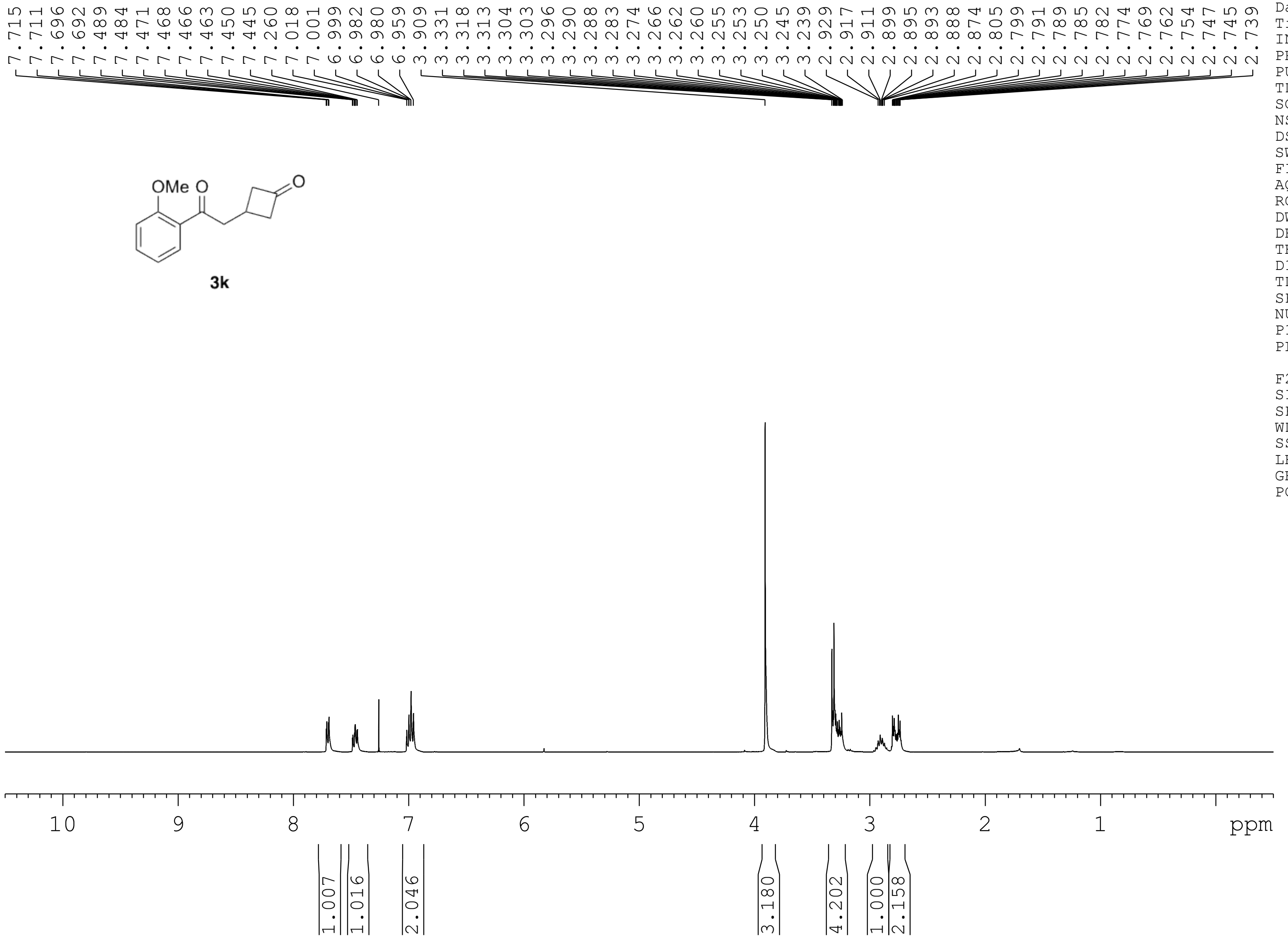


Current Data Parameters
NAME K 2OMe
EXPNO 1
PROCNO 1

F2 - Acquisition Parameters
Date_ 20240229
Time 15.34 h
INSTRUM spect
PROBHD Z108618_0411 (
PULPROG zg30
TD 32768
SOLVENT CDCl3
NS 12
DS 0
SWH 8802.817 Hz
FIDRES 0.537281 Hz
AQ 1.8612224 sec
RG 71.05
DW 56.800 usec
DE 14.47 usec
TE 298.0 K
D1 1.00000000 sec
TD0 1
SFO1 400.1328009 MHz
NUC1 1H
P1 15.50 usec
PLW1 9.89999962 W

F2 - Processing parameters
SI 131072
SF 400.1300094 MHz
WDW EM
SSB 0
LB 0 Hz
GB 0
PC 1.00

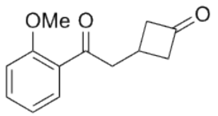


**3k**

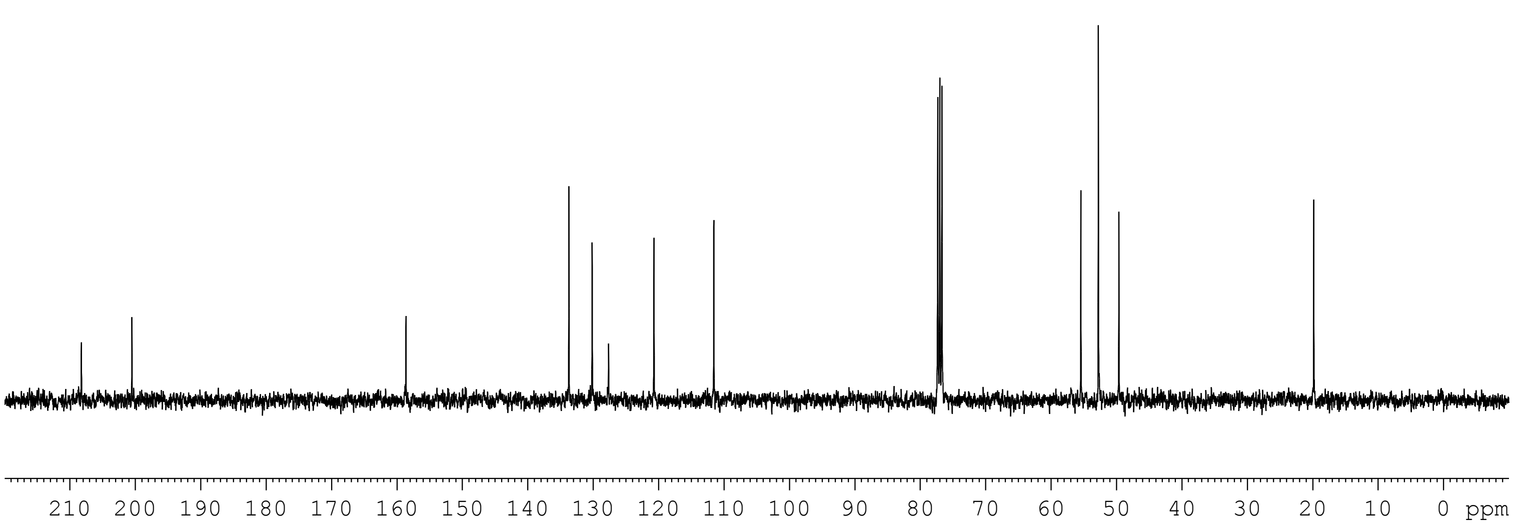


```
Current Data Parameters
NAME              K 2OMe
EXPNO                  2
PROCNO                 1

F2 - Acquisition Parameters
Date_           20240229
Time               15.36 h
INSTRUM            spect
PROBHD   Z108618_0411 (
PULPROG          zgpg30
TD                 65536
SOLVENT            CDCl3
NS                    24
DS                     4
SWH            28409.092 Hz
FIDRES          0.866977 Hz
AQ             1.1534336 sec
RG                212.49
DW                17.600 usec
DE                  6.50 usec
TE                 298.1 K
D1            2.00000000 sec
D11           0.03000000 sec
TD0                    1
SFO1         100.6258487 MHz
NUC1                 13C
P1                 10.50 usec
PLW1         42.50000000 W
SFO2         400.1316005 MHz
NUC2                  1H
CPDPRG[2         waltz16
PCPD2              90.00 usec
PLW2          9.89999962 W
PLW12         0.29363999 W
PLW13         0.14747000 W

F2 - Processing parameters
SI                 32768
SF           100.6127756 MHz
WDW                   EM
SSB                    0
LB                  3.00 Hz
GB                     0
PC                  1.40
```

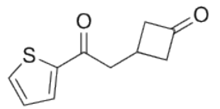

3l

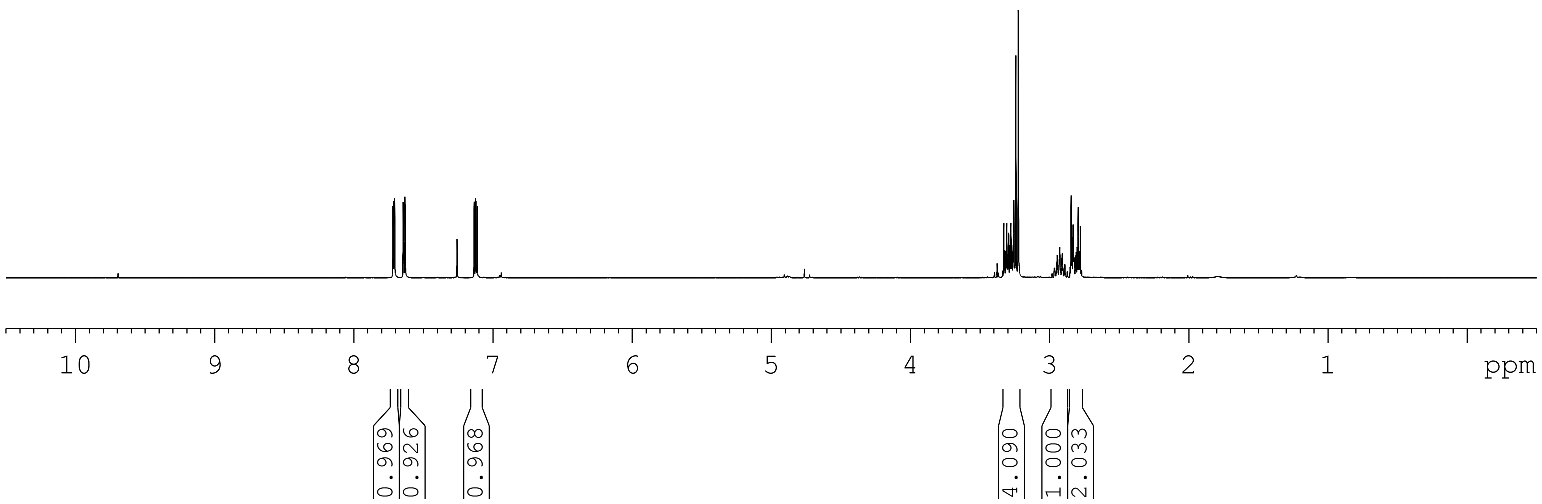


Current Data Parameters
NAME K 2-thienyl
EXPNO 2
PROCNO 1

F2 - Acquisition Parameters
Date_ 20231227
Time 16.33 h
INSTRUM spect
PROBHD Z108618_0411 (
PULPROG zg30
TD 32768
SOLVENT CDCl3
NS 33
DS 0
SWH 8802.817 Hz
FIDRES 0.537281 Hz
AQ 1.8612224 sec
RG 71.05
DW 56.800 usec
DE 14.47 usec
TE 298.0 K
D1 1.00000000 sec
TD0 1
SFO1 400.1328009 MHz
NUC1 1H
P1 15.50 usec
PLW1 9.89999962 W

F2 - Processing parameters
SI 131072
SF 400.1300097 MHz
WDW EM
SSB 0
LB 0 Hz
GB 0
PC 1.00

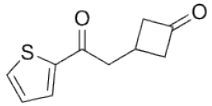

3l

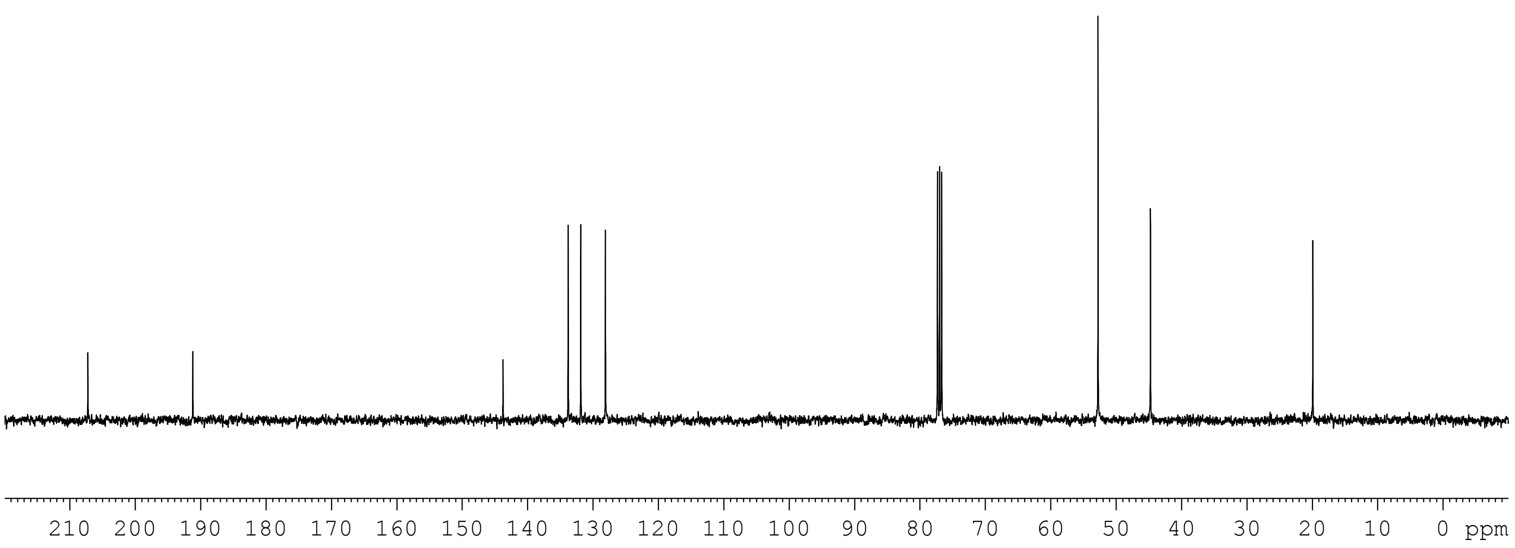


```
Current Data Parameters
NAME        K 2-thienyl
EXPNO                 3
PROCNO                1

F2 - Acquisition Parameters
Date_          20231227
Time              16.36 h
INSTRUM           spect
PROBHD   Z108618_0411 (
PULPROG          zgpg30
TD                65536
SOLVENT           CDCl3
NS                   31
DS                    4
SWH           28409.092 Hz
FIDRES         0.866977 Hz
AQ            1.1534336 sec
RG               212.49
DW               17.600 usec
DE                 6.50 usec
TE                298.1 K
D1           2.00000000 sec
D11          0.03000000 sec
TD0                   1
SFO1       100.6258487 MHz
NUC1                13C
P1                10.50 usec
PLW1        42.50000000 W
SFO2       400.1316005 MHz
NUC2                 1H
CPDPRG[2        waltz16
PCPD2             90.00 usec
PLW2         9.89999962 W
PLW12        0.29363999 W
PLW13        0.14747000 W

F2 - Processing parameters
SI                32768
SF          100.6127798 MHz
WDW                  EM
SSB                   0
LB                 3.00 Hz
GB                    0
PC                 1.40
```

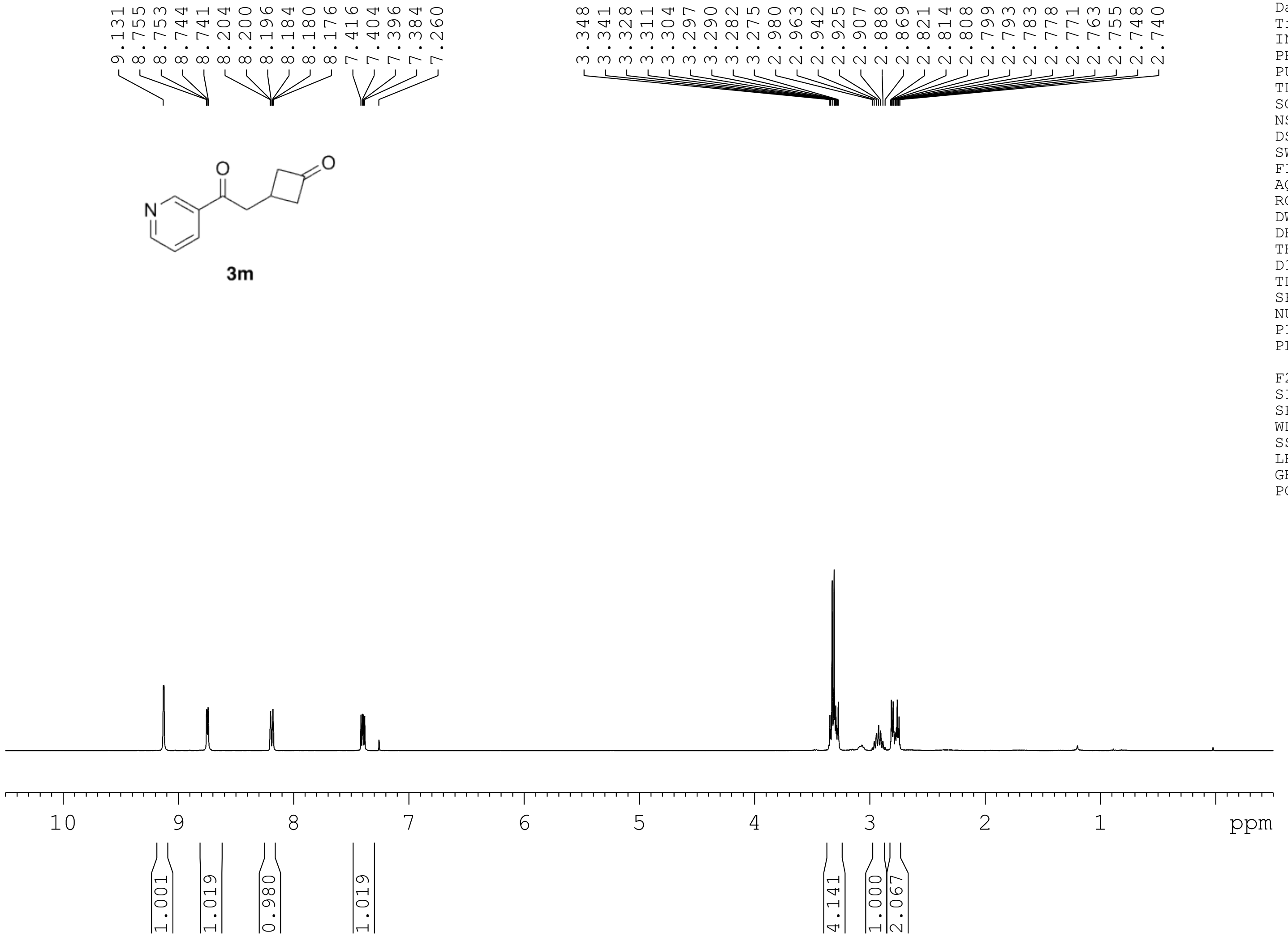


Current Data Parameters
NAME 20240412-pyridine oxidation hnmr
EXPNO 4
PROCNO 1

F2 - Acquisition Parameters
Date_ 20240412
Time_ 11.19 h
INSTRUM spect
PROBHD Z108618_0411 (
PULPROG zg30
TD 32768
SOLVENT CDCl3
NS 6
DS 0
SWH 8802.817 Hz
FIDRES 0.537281 Hz
AQ 1.8612224 sec
RG 71.05
DW 56.800 usec
DE 14.47 usec
TE 298.0 K
D1 1.00000000 sec
TD0 1
SFO1 400.1328009 MHz
NUC1 1H
P1 15.50 usec
PLW1 9.89999962 W

F2 - Processing parameters
SI 131072
SF 400.1300094 MHz
WDW EM
SSB 0
LB 0 Hz
GB 0
PC 1.00

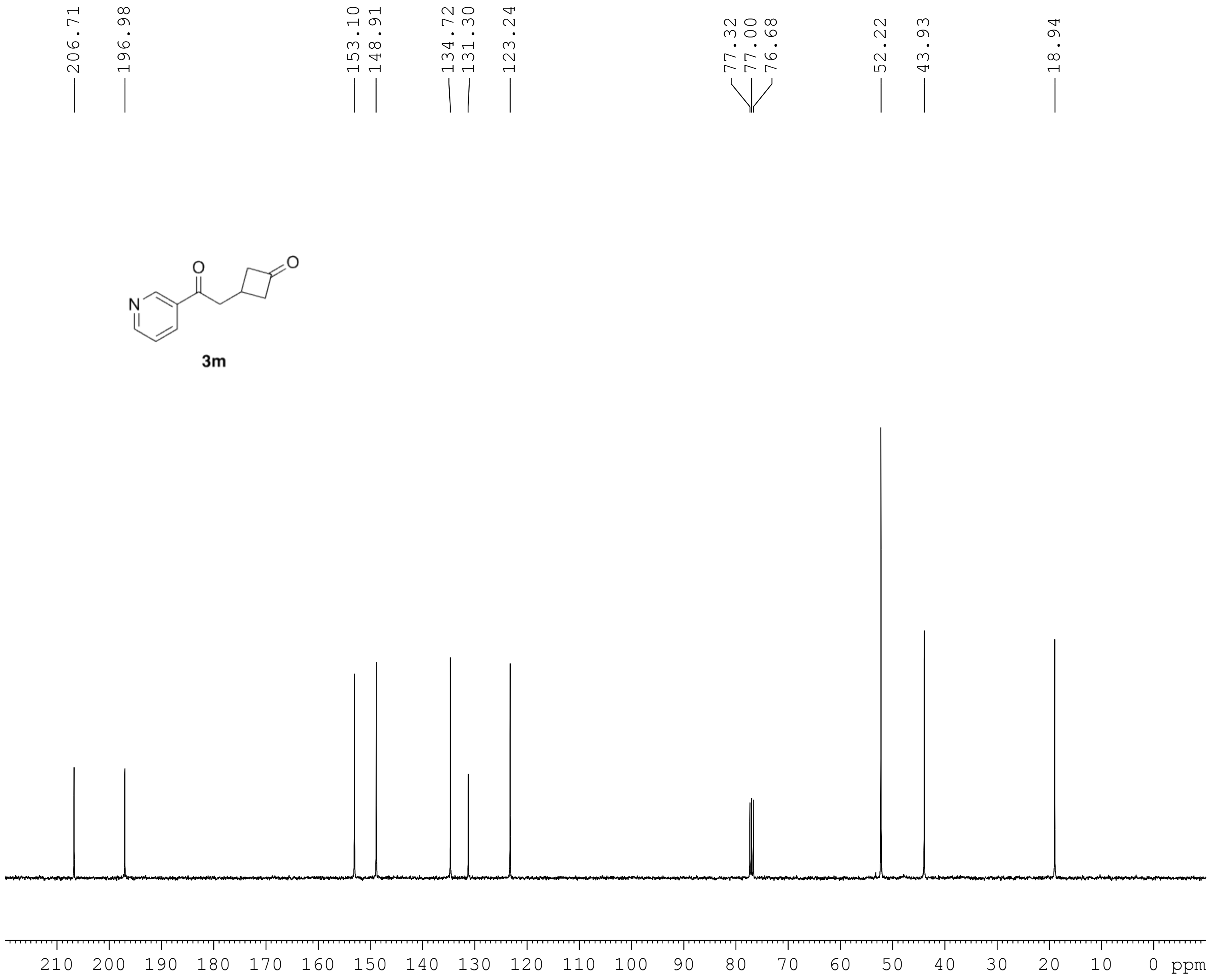

3m



Current Data Parameters
NAME K py C
EXPNO 2
PROCNO 1

F2 - Acquisition Parameters
Date_ 20240411
Time 18.04 h
INSTRUM spect
PROBHD Z108618_0411 (
PULPROG zgpg30
TD 65536
SOLVENT CDCl3
NS 24
DS 4
SWH 28409.092 Hz
FIDRES 0.866977 Hz
AQ 1.1534336 sec
RG 212.49
DW 17.600 usec
DE 6.50 usec
TE 298.1 K
D1 2.00000000 sec
D11 0.03000000 sec
TD0 1
SFO1 100.6258487 MHz
NUC1 13C
P1 10.50 usec
PLW1 42.50000000 W
SFO2 400.1316005 MHz
NUC2 1H
CPDPRG[2 waltz16
PCPD2 90.00 usec
PLW2 9.89999962 W
PLW12 0.29363999 W
PLW13 0.14747000 W

F2 - Processing parameters
SI 32768
SF 100.6128079 MHz
WDW EM
SSB 0
LB 3.00 Hz
GB 0
PC 1.40

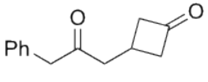

**3n**

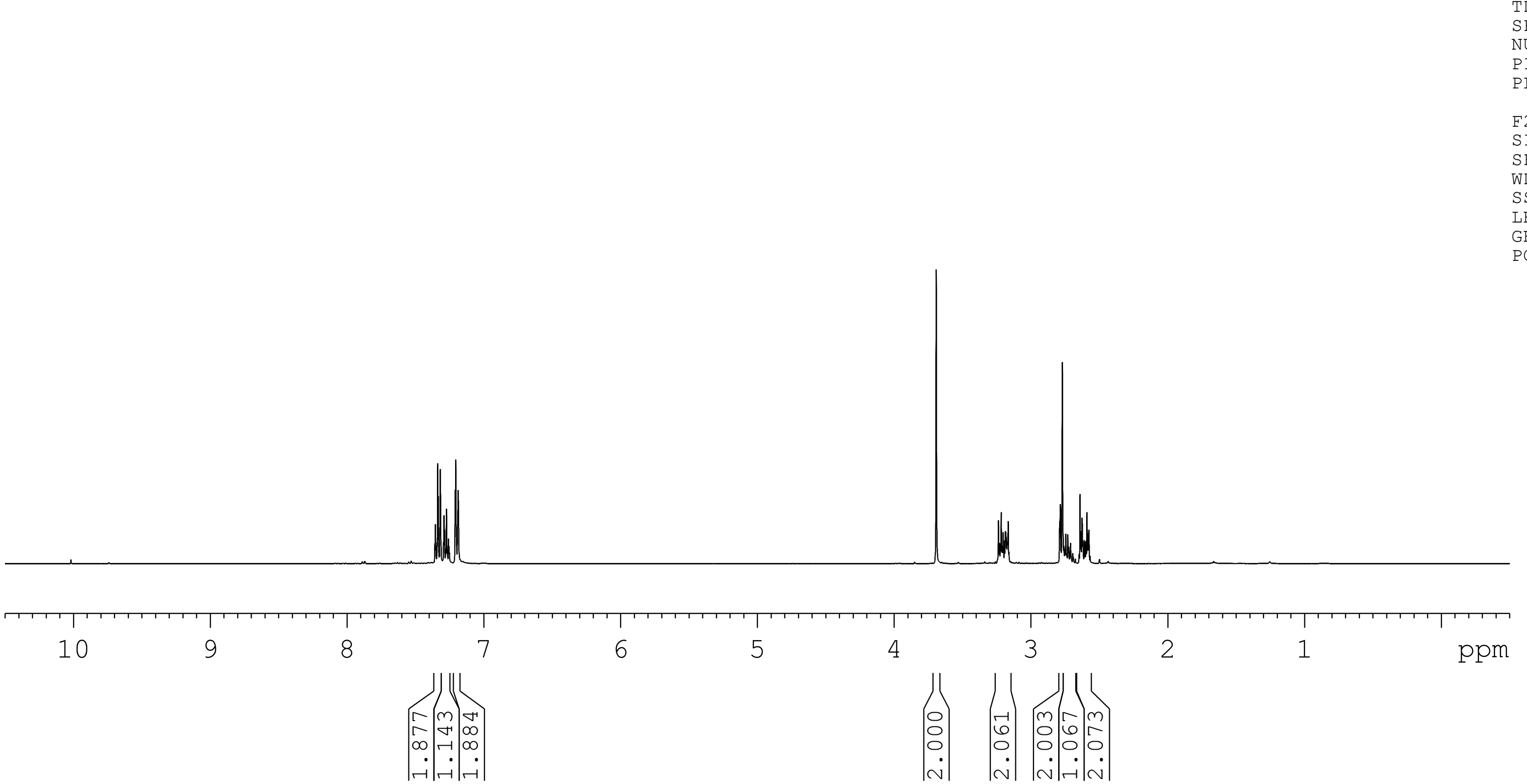


```
Current Data Parameters
NAME              K benzyl
EXPNO                    1
PROCNO                   1

F2 - Acquisition Parameters
Date_             20240619
Time                 13.12 h
INSTRUM              spect
PROBHD    Z108618_0411 (
PULPROG               zg30
TD                   32768
SOLVENT              CDCl3
NS                      20
DS                       0
SWH               8802.817 Hz
FIDRES            0.537281 Hz
AQ               1.8612224 sec
RG                   94.01
DW                  56.800 usec
DE                   14.47 usec
TE                   298.0 K
D1              1.00000000 sec
TD0                      1
SFO1        400.1328009 MHz
NUC1                    1H
P1                   15.50 usec
PLW1            9.89999962 W

F2 - Processing parameters
SI                  131072
SF          400.1300095 MHz
WDW                     EM
SSB                      0
LB                       0 Hz
GB                       0
PC                    1.00
```

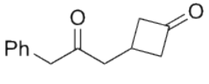


**3n**

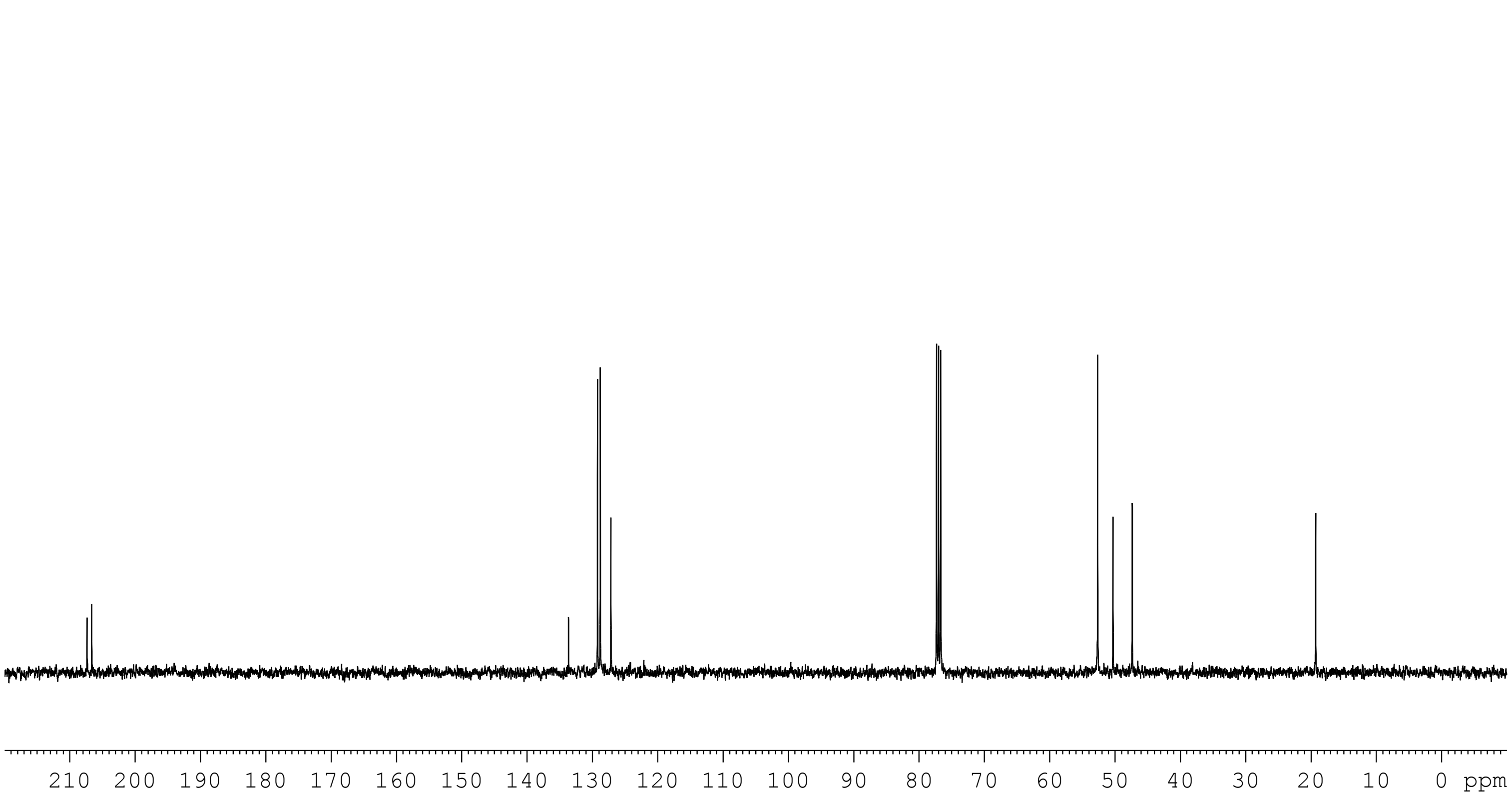


Current Data Parameters
NAME K benzyl
EXPNO 2
PROCNO 1

F2 - Acquisition Parameters
Date_ 20240619
Time_ 13.16 h
INSTRUM spect
PROBHD Z108618_0411 (
PULPROG zgpg30
TD 65536
SOLVENT CDCl3
NS 46
DS 4
SWH 28409.092 Hz
FIDRES 0.866977 Hz
AQ 1.1534336 sec
RG 212.49
DW 17.600 usec
DE 6.50 usec
TE 298.0 K
D1 2.00000000 sec
D11 0.03000000 sec
TD0 1
SFO1 100.6258487 MHz
NUC1 13C
P1 10.50 usec
PLW1 42.50000000 W
SFO2 400.1316005 MHz
NUC2 1H
CPDPRG[2 waltz16
PCPD2 90.00 usec
PLW2 9.89999962 W
PLW12 0.29363999 W
PLW13 0.14747000 W

F2 - Processing parameters
SI 32768
SF 100.6127759 MHz
WDW EM
SSB 0
LB 3.00 Hz
GB 0
PC 1.40

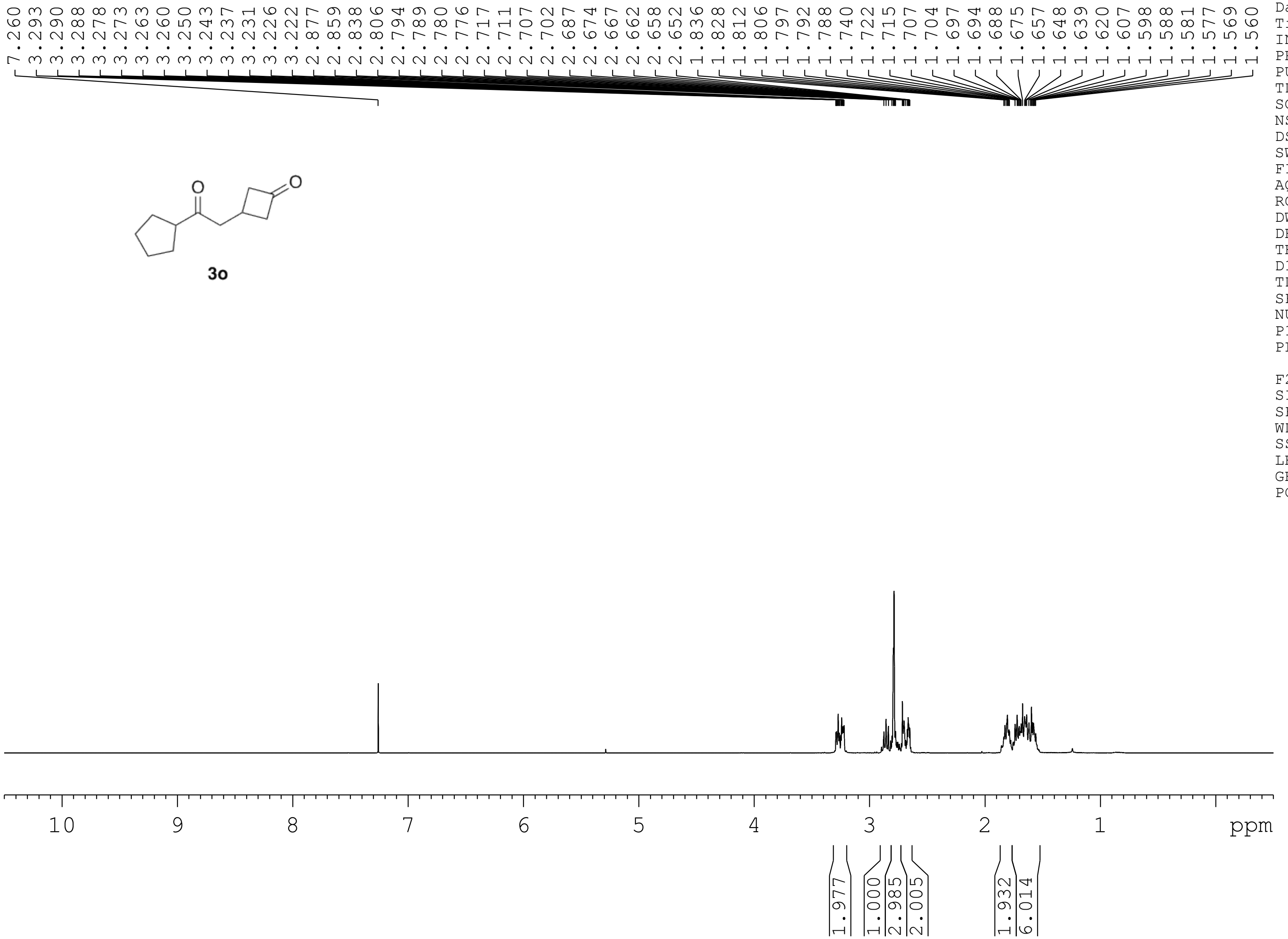


Current Data Parameters
NAME K cyclopentane
EXPNO 1
PROCNO 1

F2 - Acquisition Parameters
Date_ 20240112
Time 16.14 h
INSTRUM spect
PROBHD Z8400_0196 (PH
PULPROG zg30
TD 32768
SOLVENT CDCl3
NS 24
DS 0
SWH 8802.817 Hz
FIDRES 0.537281 Hz
AQ 1.8612224 sec
RG 133.5
DW 56.800 usec
DE 14.57 usec
TE 298.0 K
D1 1.00000000 sec
TD0 1
SFO1 400.1328009 MHz
NUC1 1H
P1 15.00 usec
PLW1 9.89999962 W

F2 - Processing parameters
SI 131072
SF 400.1300096 MHz
WDW EM
SSB 0
LB 0 Hz
GB 0
PC 1.00

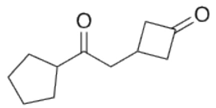

**3o**

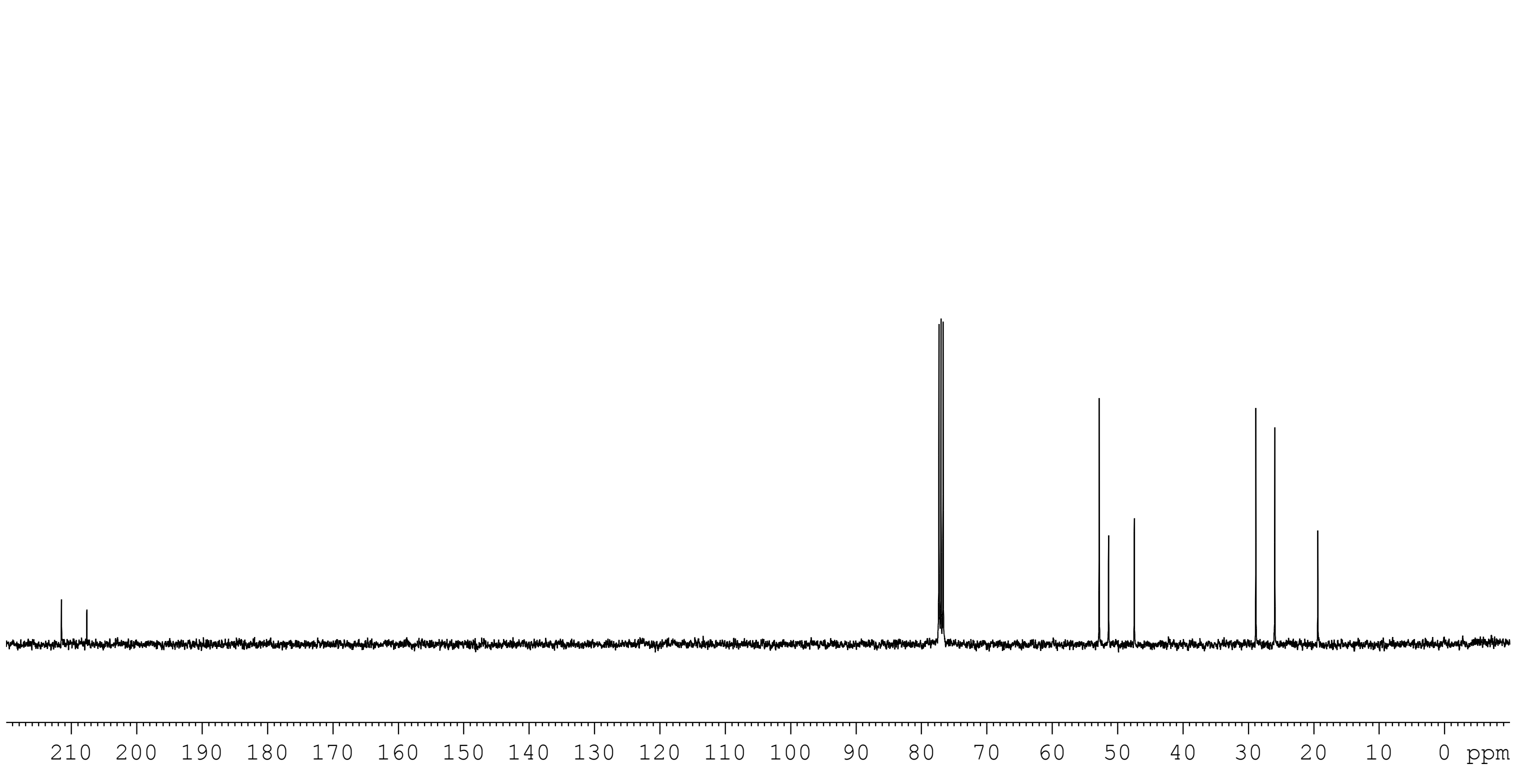


```
Current Data Parameters
NAME      K cyclopentane
EXPNO                  3
PROCNO                 1

F2 - Acquisition Parameters
Date_           20240112
Time               16.23 h
INSTRUM            spect
PROBHD    Z8400_0196 (PH
PULPROG          zgpg30
TD                 65536
SOLVENT            CDCl3
NS                   108
DS                     4
SWH            28409.092 Hz
FIDRES          0.866977 Hz
AQ             1.1534336 sec
RG                212.49
DW                17.600 usec
DE                  6.50 usec
TE                 298.0 K
D1            2.00000000 sec
D11           0.03000000 sec
TD0                    1
SFO1         100.6258487 MHz
NUC1                 13C
P1                 10.00 usec
PLW1         47.00000000 W
SFO2         400.1316005 MHz
NUC2                  1H
CPDPRG[2         waltz16
PCPD2              90.00 usec
PLW2          9.89999962 W
PLW12         0.27500001 W
PLW13         0.13832000 W

F2 - Processing parameters
SI                 32768
SF           100.6127721 MHz
WDW                   EM
SSB                    0
LB                  3.00 Hz
GB                     0
PC                  1.40
```

Current Data Parameters
NAME R Ph
EXPNO 1
PROCNO 1

F2 - Acquisition Parameters
Date_ 20240312
Time 17.39 h
INSTRUM spect
PROBHD Z108618_0411 (
PULPROG zg30
TD 32768
SOLVENT CDCl3
NS 17
DS 0
SWH 8802.817 Hz
FIDRES 0.537281 Hz
AQ 1.8612224 sec
RG 71.05
DW 56.800 usec
DE 14.47 usec
TE 298.0 K
D1 1.00000000 sec
TD0 1
SFO1 400.1328009 MHz
NUC1 1H
P1 15.50 usec
PLW1 9.89999962 W

F2 - Processing parameters
SI 131072
SF 400.1300096 MHz
WDW EM
SSB 0
LB 0 Hz
GB 0
PC 1.00

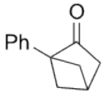


**5a**

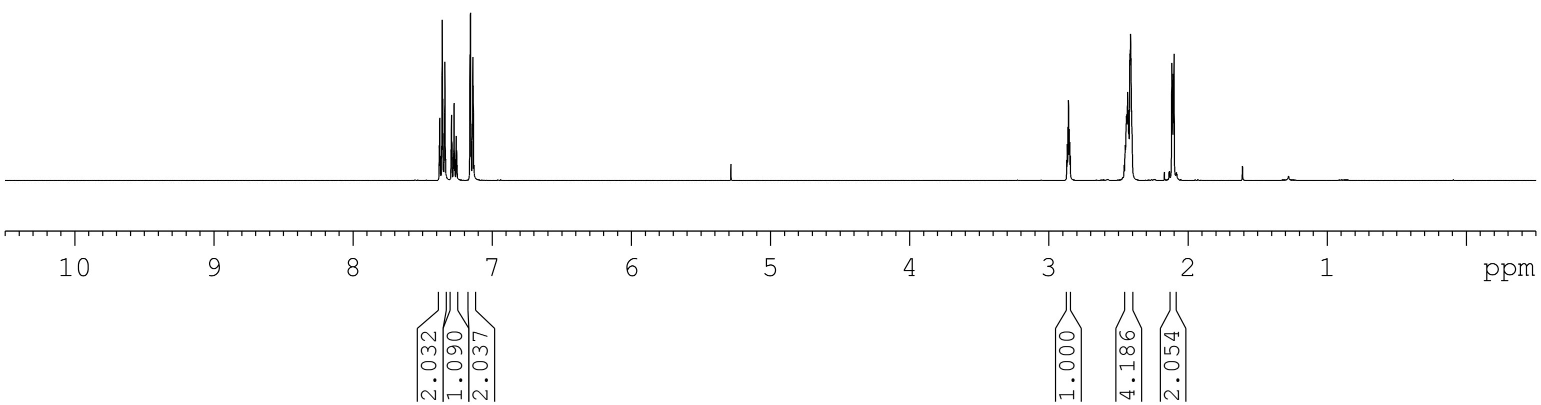

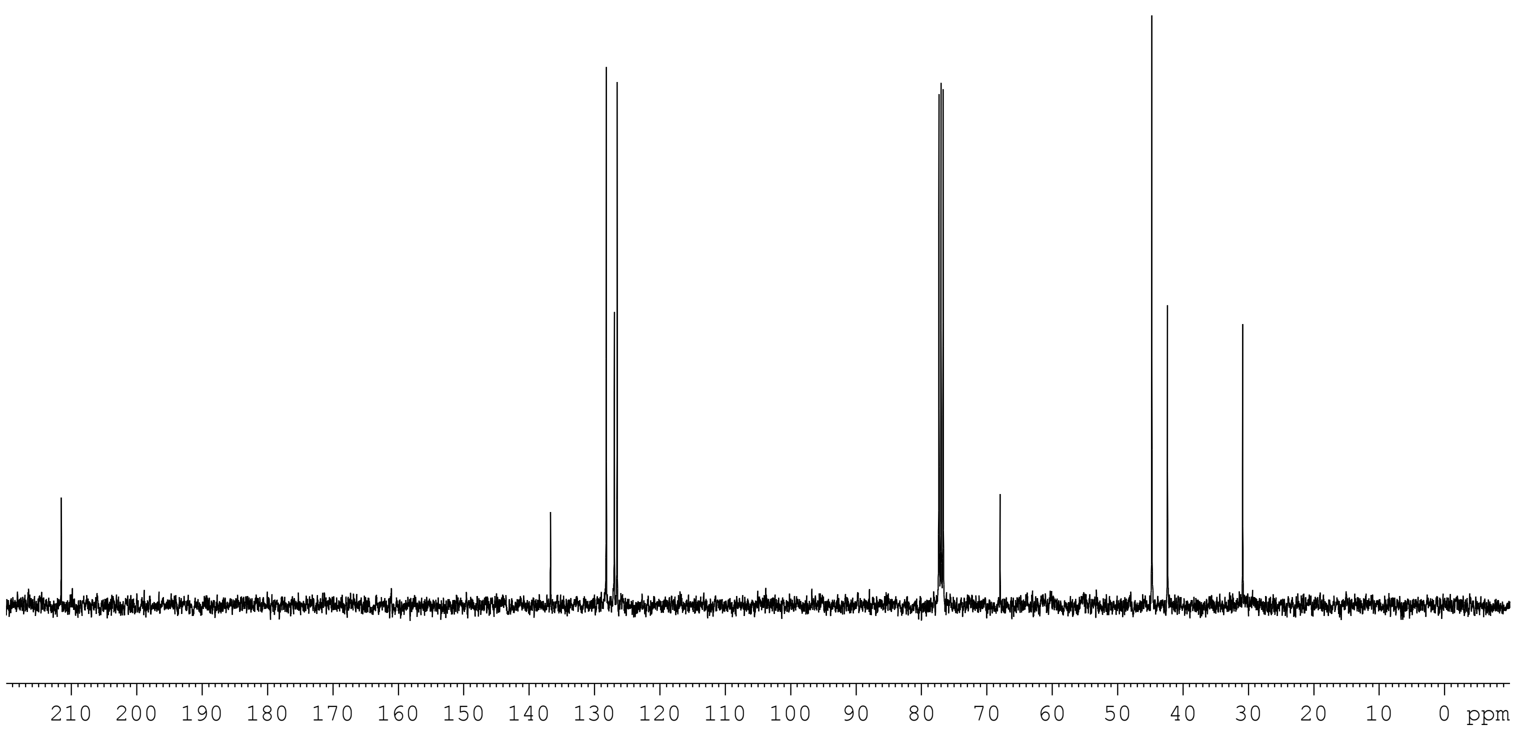


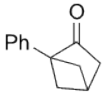


**5a**

```
Current Data Parameters
NAME                 R Ph
EXPNO                   2
PROCNO                  1

F2 - Acquisition Parameters
Date_            20240312
Time                17.44 h
INSTRUM             spect
PROBHD   Z108618_0411 (
PULPROG           zgpg30
TD                 65536
SOLVENT            CDCl3
NS                     1
DS                     4
SWH            28409.092 Hz
FIDRES          0.866977 Hz
AQ             1.1534336 sec
RG                212.49
DW                17.600 usec
DE                  6.50 usec
TE                 298.0 K
D1            2.00000000 sec
D11           0.03000000 sec
TD0                    1
SFO1         100.6258487 MHz
NUC1                 13C
P1                 10.50 usec
PLW1         42.50000000 W
SFO2         400.1316005 MHz
NUC2                  1H
CPDPRG[2         waltz16
PCPD2              90.00 usec
PLW2          9.89999962 W
PLW12         0.29363999 W
PLW13         0.14747000 W

F2 - Processing parameters
SI                 32768
SF           100.6127777 MHz
WDW                   EM
SSB                    0
LB                  3.00 Hz
GB                     0
PC                  1.40
```

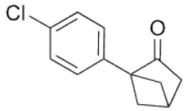

**5b**

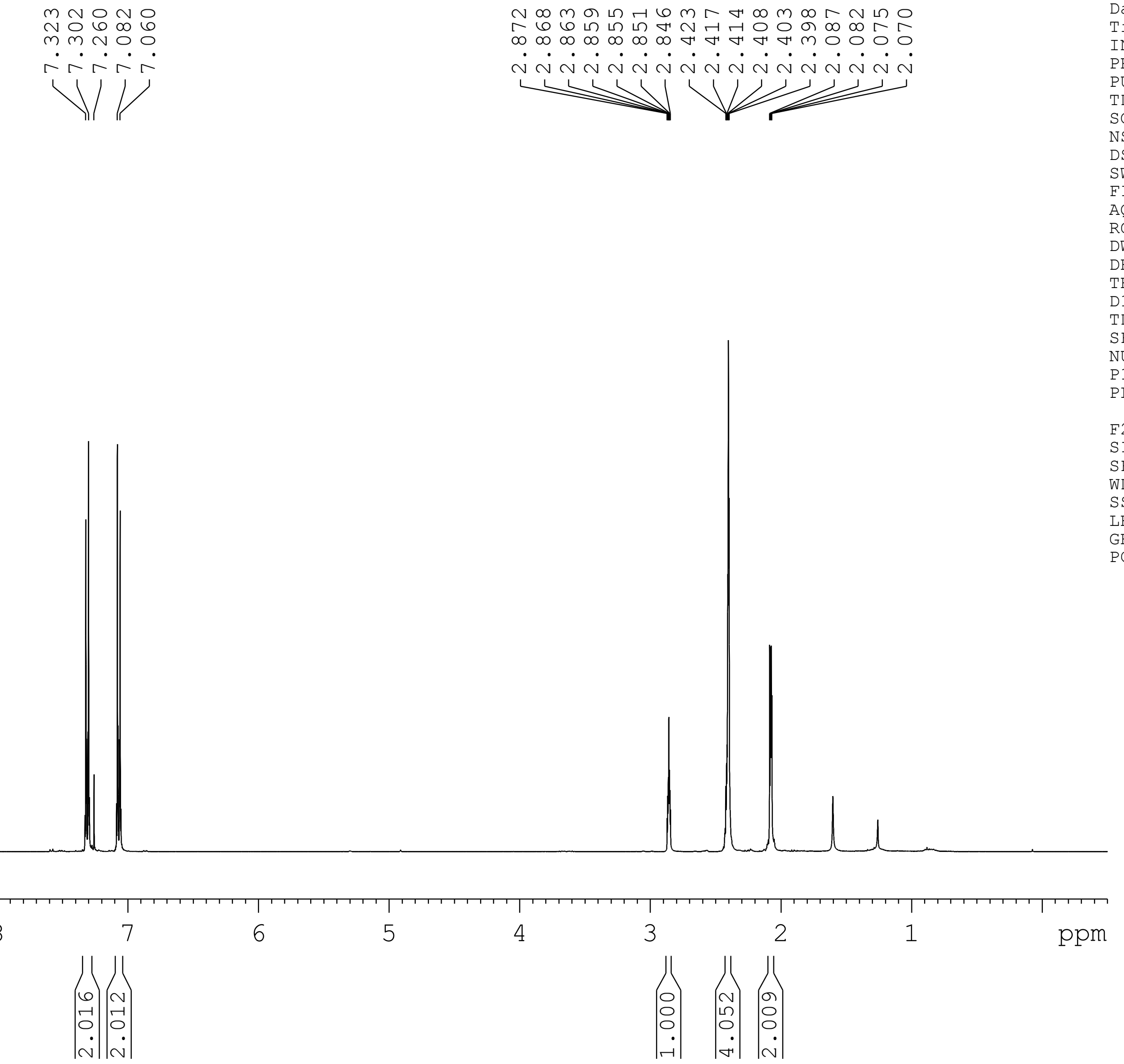


```
Current Data Parameters
NAME      20231102_4-Cl
EXPNO                 4
PROCNO                1

F2 - Acquisition Parameters
Date_          20231102
Time              20.43 h
INSTRUM           spect
PROBHD   Z108618_0411 (
PULPROG            zg30
TD                32768
SOLVENT           CDCl3
NS                   17
DS                    0
SWH            8802.817 Hz
FIDRES         0.537281 Hz
AQ            1.8612224 sec
RG               143.54
DW               56.800 usec
DE                14.47 usec
TE                298.0 K
D1           1.00000000 sec
TD0                   1
SFO1        400.1328009 MHz
NUC1                 1H
P1                15.50 usec
PLW1         9.89999962 W

F2 - Processing parameters
SI               131072
SF          400.1300096 MHz
WDW                  EM
SSB                   0
LB                    0 Hz
GB                    0
PC                 1.00
```

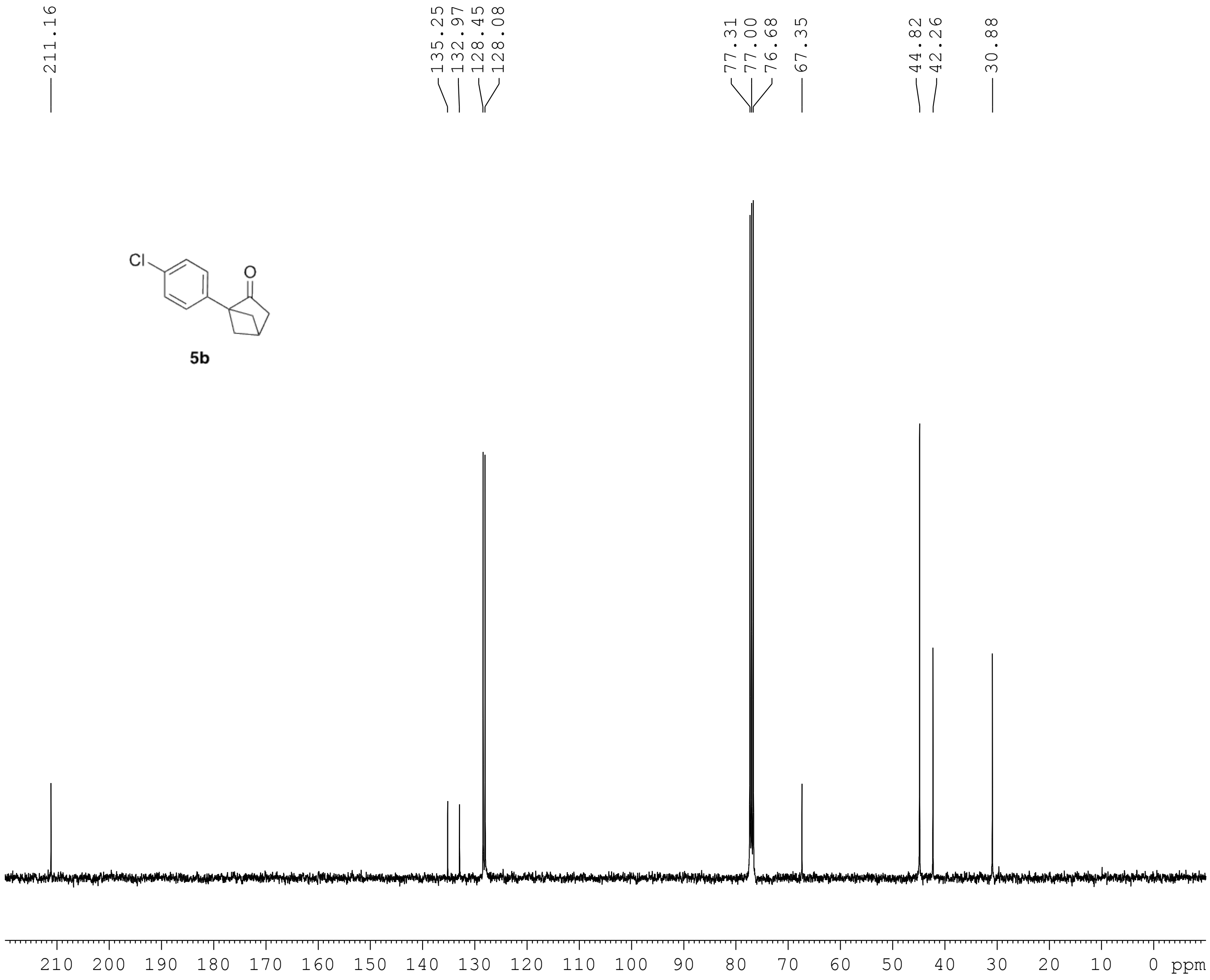


```
Current Data Parameters
NAME              R 4-Cl
EXPNO                  5
PROCNO                 1

F2 - Acquisition Parameters
Date_           20231102
Time               20.55 h
INSTRUM            spect
PROBHD   Z108618_0411 (
PULPROG          zgpg30
TD                 65536
SOLVENT            CDCl3
NS                   187
DS                     4
SWH            28409.092 Hz
FIDRES          0.866977 Hz
AQ             1.1534336 sec
RG                212.49
DW                17.600 usec
DE                  6.50 usec
TE                 298.1 K
D1            2.00000000 sec
D11           0.03000000 sec
TD0                    1
SFO1         100.6258487 MHz
NUC1                 13C
P1                 10.50 usec
PLW1         42.50000000 W
SFO2         400.1316005 MHz
NUC2                  1H
CPDPRG[2         waltz16
PCPD2              90.00 usec
PLW2          9.89999962 W
PLW12         0.29363999 W
PLW13         0.14747000 W

F2 - Processing parameters
SI                 32768
SF           100.6127738 MHz
WDW                   EM
SSB                    0
LB                  3.00 Hz
GB                     0
PC                  1.40
```

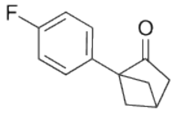

**5c**

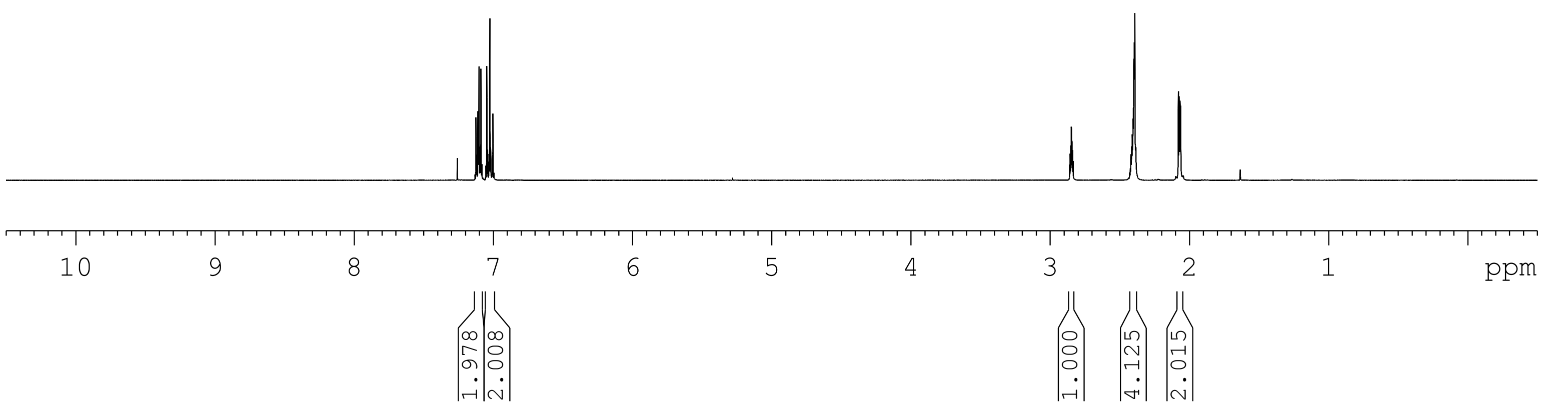


```
Current Data Parameters
NAME              R 4F
EXPNO                1
PROCNO               1

F2 - Acquisition Parameters
Date_          20240312
Time              17.46 h
INSTRUM           spect
PROBHD   Z108618_0411 (
PULPROG            zg30
TD                32768
SOLVENT           CDCl3
NS                   14
DS                    0
SWH            8802.817 Hz
FIDRES         0.537281 Hz
AQ            1.8612224 sec
RG                71.05
DW               56.800 usec
DE                14.47 usec
TE                298.0 K
D1           1.00000000 sec
TD0                   1
SFO1        400.1328009 MHz
NUC1                 1H
P1                15.50 usec
PLW1         9.89999962 W

F2 - Processing parameters
SI               131072
SF          400.1300095 MHz
WDW                  EM
SSB                   0
LB                    0 Hz
GB                    0
PC                 1.00
```

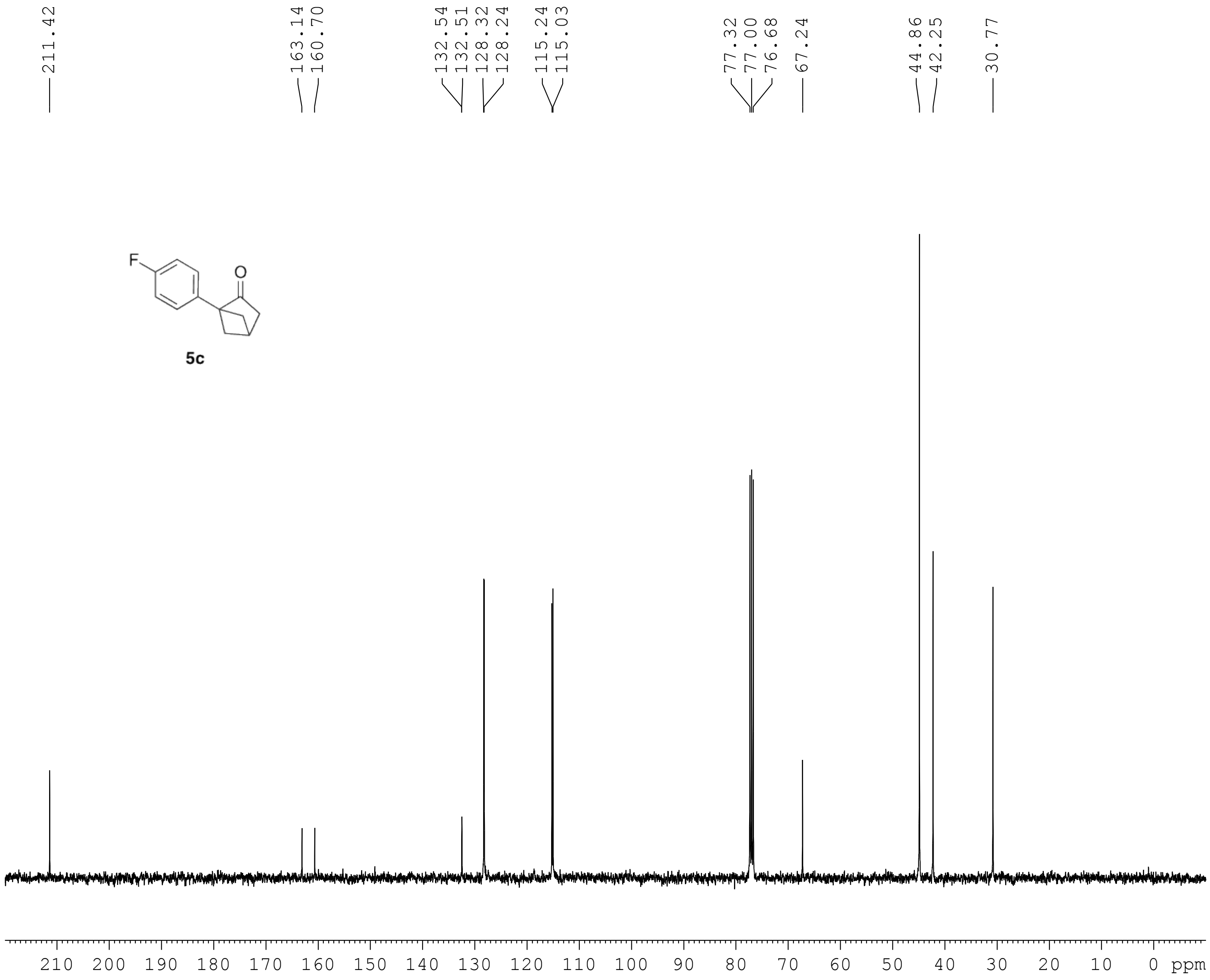


```
Current Data Parameters
NAME                R 4F
EXPNO                  2
PROCNO                 1

F2 - Acquisition Parameters
Date_           20240312
Time               17.51 h
INSTRUM            spect
PROBHD   Z108618_0411 (
PULPROG          zgpg30
TD                 65536
SOLVENT            CDCl3
NS                    59
DS                     4
SWH            28409.092 Hz
FIDRES          0.866977 Hz
AQ             1.1534336 sec
RG                212.49
DW                17.600 usec
DE                  6.50 usec
TE                 298.1 K
D1            2.00000000 sec
D11           0.03000000 sec
TD0                    1
SFO1        100.6258487 MHz
NUC1                 13C
P1                 10.50 usec
PLW1         42.50000000 W
SFO2        400.1316005 MHz
NUC2                  1H
CPDPRG[2         waltz16
PCPD2              90.00 usec
PLW2          9.89999962 W
PLW12         0.29363999 W
PLW13         0.14747000 W

F2 - Processing parameters
SI                 32768
SF           100.6127764 MHz
WDW                   EM
SSB                    0
LB                  3.00 Hz
GB                     0
PC                  1.40
```

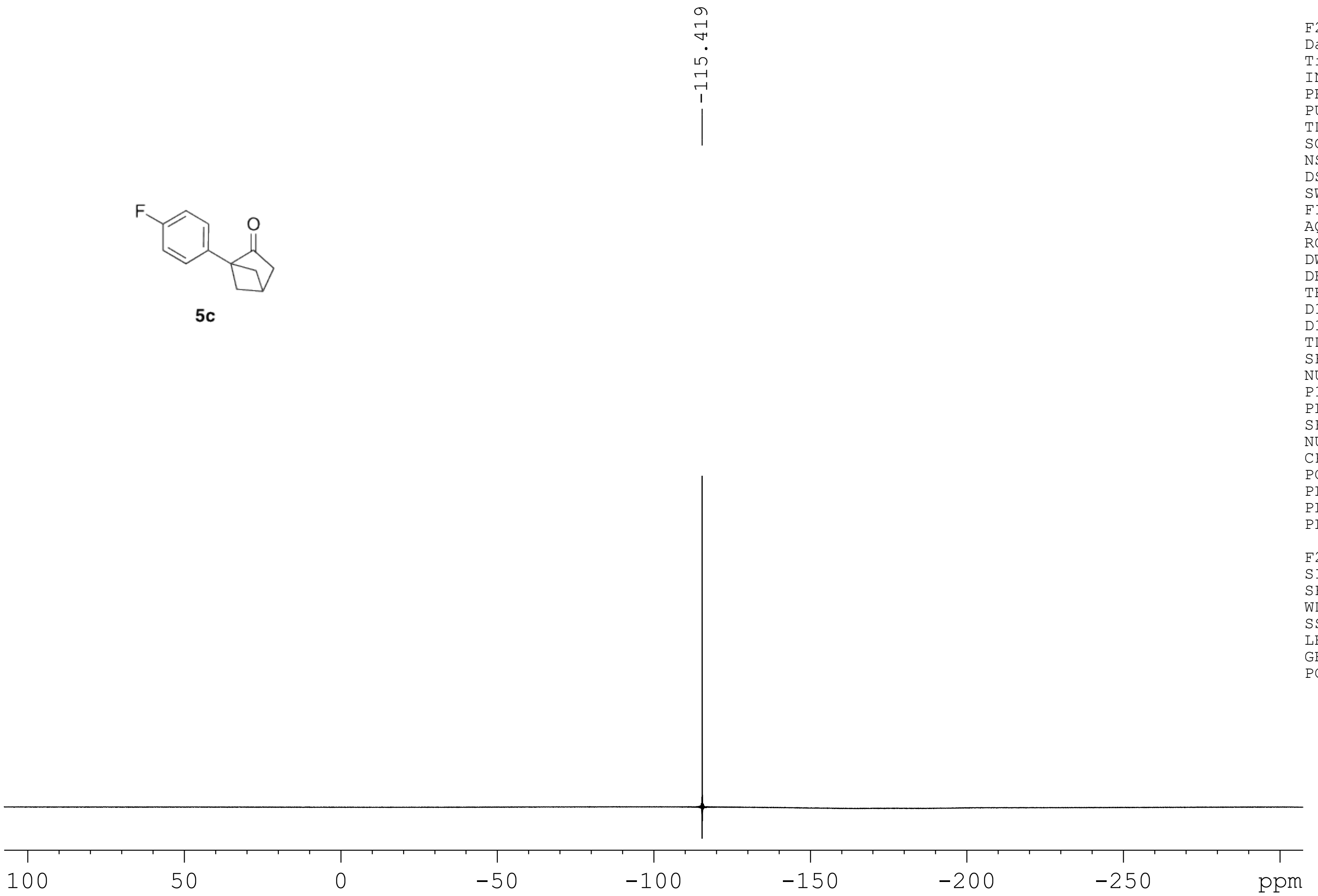


```
Current Data Parameters
NAME      20240808_R_4F
EXPNO                 1
PROCNO                1

F2 - Acquisition Parameters
Date_          20240808
Time              16.42 h
INSTRUM           spect
PROBHD   Z108618_0411 (
PULPROG         zgpg30
TD                65536
SOLVENT           CDCl3
NS                   15
DS                    2
SWH          156250.000 Hz
FIDRES         4.768372 Hz
AQ            0.2097152 sec
RG               212.49
DW                3.200 usec
DE                 6.92 usec
TE                298.0 K
D1           1.50000000 sec
D11          0.03000000 sec
TD0                   1
SFO1        376.4607162 MHz
NUC1                19F
P1                15.50 usec
PLW1        11.89999962 W
SFO2        400.1316005 MHz
NUC2                 1H
CPDPRG[2          garp4
PCPD2             90.00 usec
PLW2         9.89999962 W
PLW12        0.29363999 W
PLW13        0.14747000 W

F2 - Processing parameters
SI               131072
SF          376.4983224 MHz
WDW                  EM
SSB                   0
LB                    0 Hz
GB                    0
PC                 1.00
```

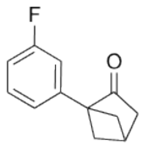


**5d**

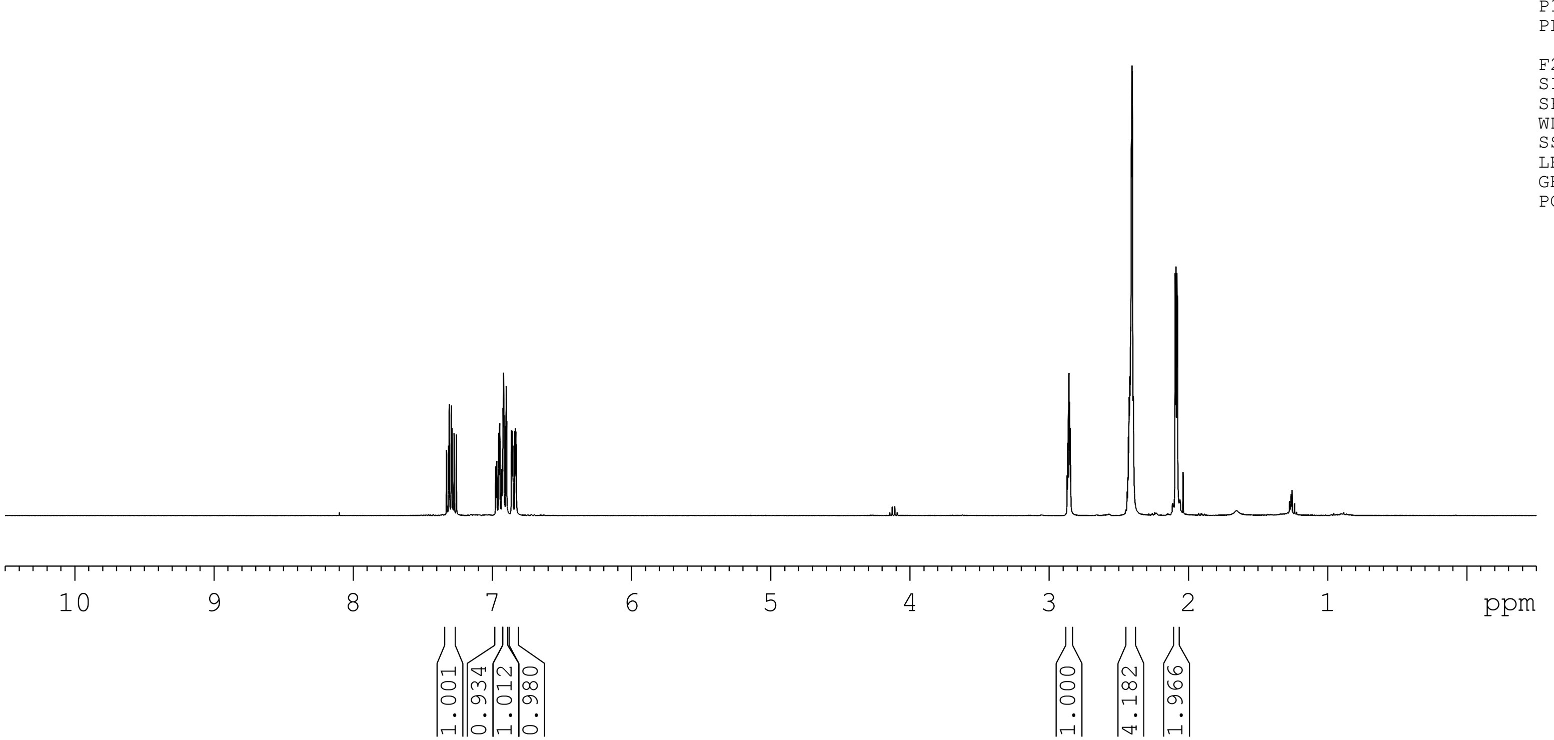


```
Current Data Parameters
NAME                R 3F
EXPNO                  1
PROCNO                 1

F2 - Acquisition Parameters
Date_           20231220
Time               17.17 h
INSTRUM            spect
PROBHD   Z108618_0411 (
PULPROG             zg30
TD                 32768
SOLVENT            CDCl3
NS                    24
DS                     0
SWH             8802.817 Hz
FIDRES          0.537281 Hz
AQ             1.8612224 sec
RG                 71.05
DW                56.800 usec
DE                 14.47 usec
TE                 298.0 K
D1            1.00000000 sec
TD0                    1
SFO1         400.1328009 MHz
NUC1                  1H
P1                 15.50 usec
PLW1          9.89999962 W

F2 - Processing parameters
SI                131072
SF           400.1300096 MHz
WDW                   EM
SSB                    0
LB                     0 Hz
GB                     0
PC                  1.00
```

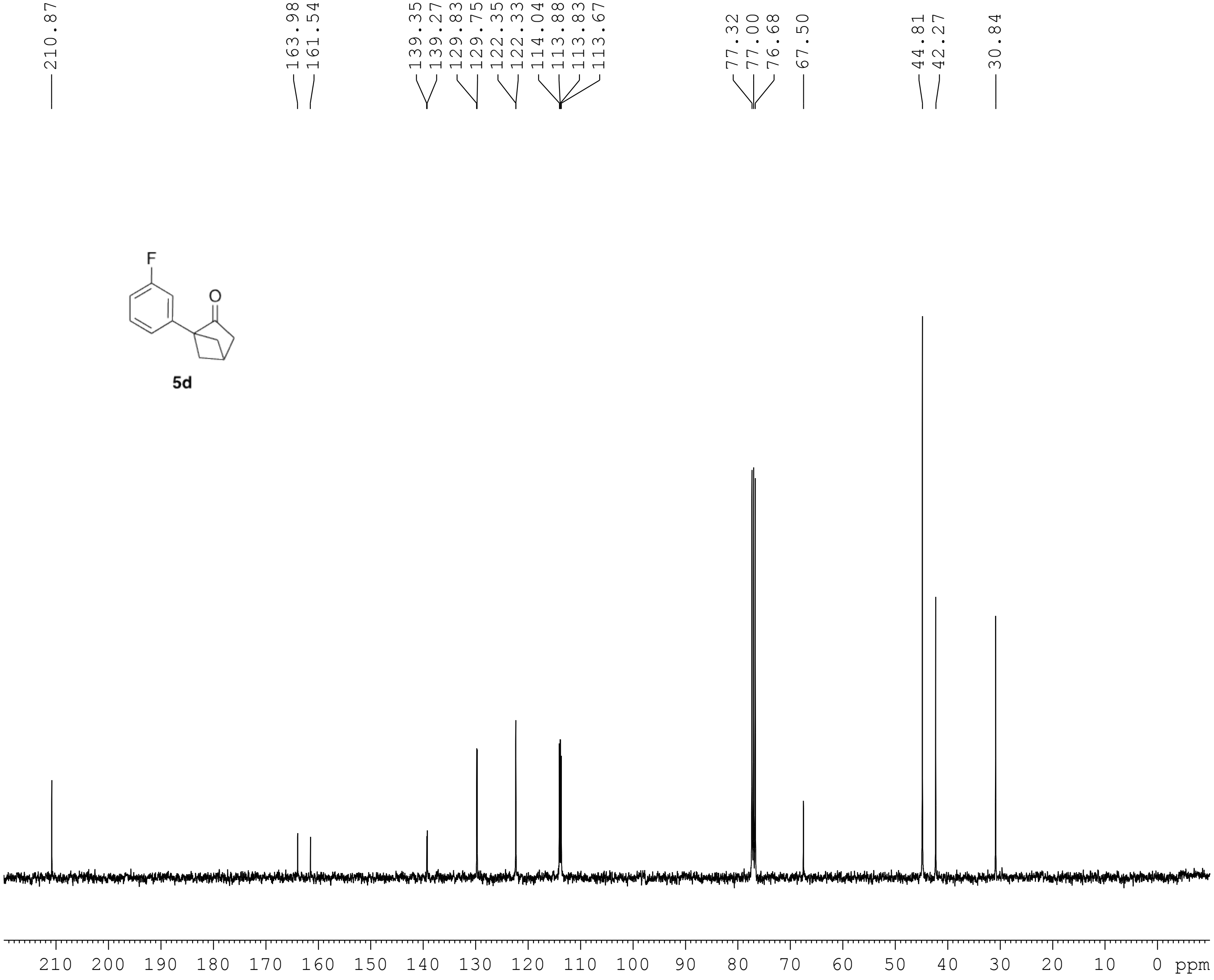


```
Current Data Parameters
NAME                R 3F
EXPNO                  2
PROCNO                 1

F2 - Acquisition Parameters
Date_           20231220
Time               17.22 h
INSTRUM            spect
PROBHD   Z108618_0411 (
PULPROG          zgpg30
TD                 65536
SOLVENT            CDCl3
NS                    67
DS                     4
SWH            28409.092 Hz
FIDRES          0.866977 Hz
AQ             1.1534336 sec
RG                212.49
DW                17.600 usec
DE                  6.50 usec
TE                 298.1 K
D1            2.00000000 sec
D11           0.03000000 sec
TD0                    1
SFO1         100.6258487 MHz
NUC1                 13C
P1                 10.50 usec
PLW1         42.50000000 W
SFO2         400.1316005 MHz
NUC2                  1H
CPDPRG[2         waltz16
PCPD2              90.00 usec
PLW2          9.89999962 W
PLW12         0.29363999 W
PLW13         0.14747000 W

F2 - Processing parameters
SI                 32768
SF           100.6127754 MHz
WDW                   EM
SSB                    0
LB                  3.00 Hz
GB                     0
PC                  1.40
```

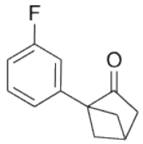

**5d**

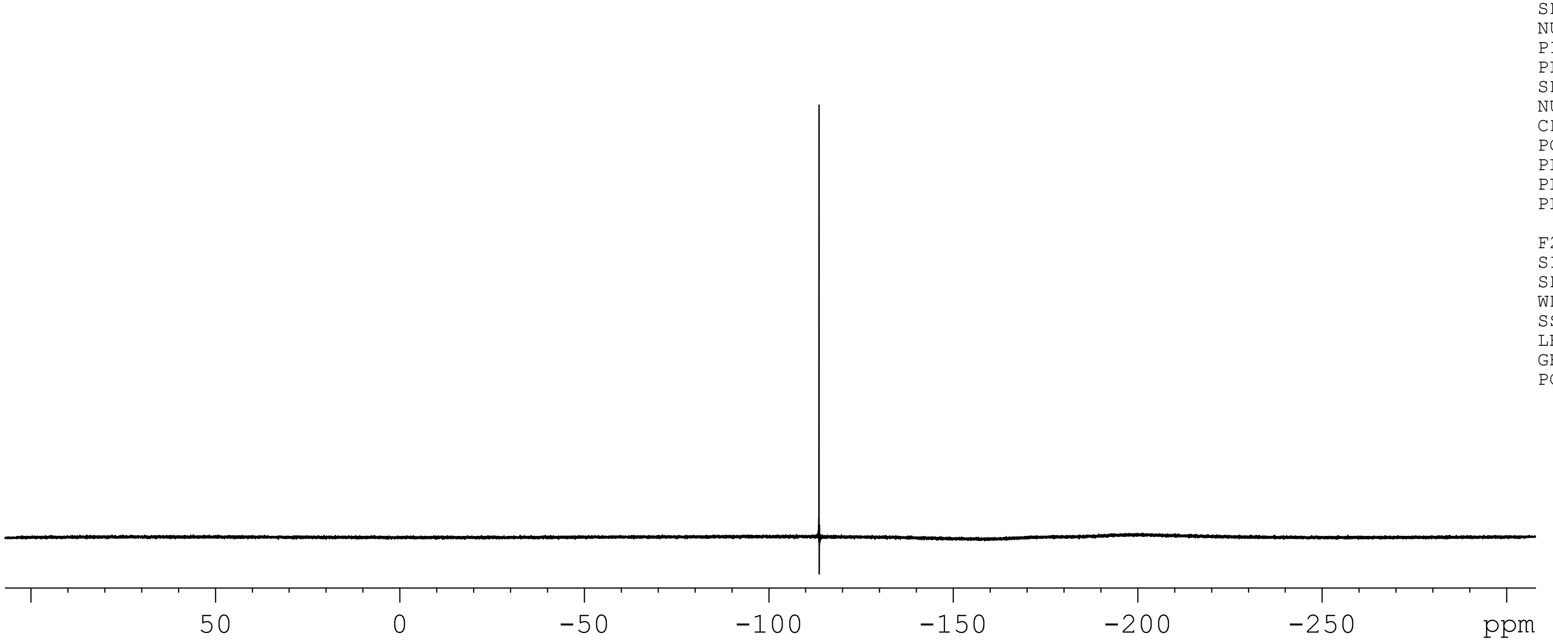


```
Current Data Parameters
NAME      20240808_R_3F
EXPNO                 1
PROCNO                1

F2 - Acquisition Parameters
Date_          20240808
Time              16.50 h
INSTRUM           spect
PROBHD   Z108618_0411 (
PULPROG         zgpg30
TD                65536
SOLVENT           CDCl3
NS                   11
DS                    2
SWH          156250.000 Hz
FIDRES         4.768372 Hz
AQ            0.2097152 sec
RG               212.49
DW                3.200 usec
DE                 6.92 usec
TE                298.0 K
D1           1.50000000 sec
D11          0.03000000 sec
TD0                   1
SFO1        376.4607162 MHz
NUC1                19F
P1                15.50 usec
PLW1        11.89999962 W
SFO2        400.1316005 MHz
NUC2                 1H
CPDPRG[2          garp4
PCPD2             90.00 usec
PLW2         9.89999962 W
PLW12        0.29363999 W
PLW13        0.14747000 W

F2 - Processing parameters
SI               131072
SF          376.4985168 MHz
WDW                  EM
SSB                   0
LB                    0 Hz
GB                    0
PC                 1.00
```

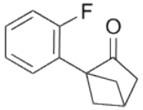


**5e**

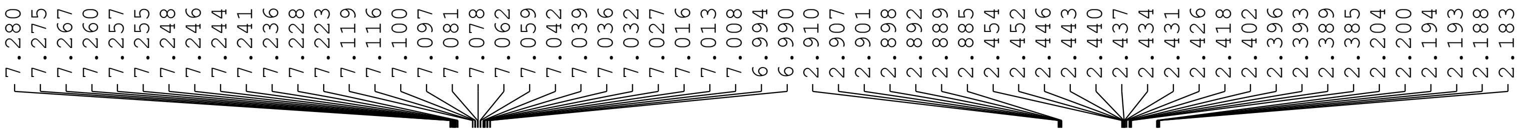


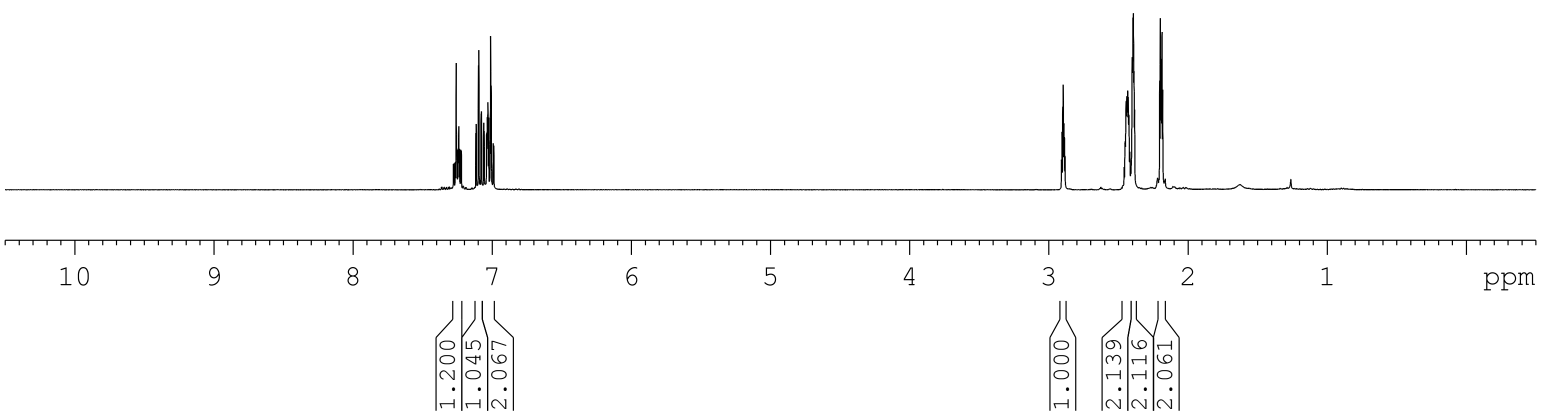


```
Current Data Parameters
NAME               R 2F H
EXPNO                   6
PROCNO                  1

F2 - Acquisition Parameters
Date_            20240302
Time                10.50 h
INSTRUM             spect
PROBHD   Z108618_0411 (
PULPROG              zg30
TD                  32768
SOLVENT             CDCl3
NS                      7
DS                      0
SWH              8802.817 Hz
FIDRES           0.537281 Hz
AQ              1.8612224 sec
RG                 103.36
DW                 56.800 usec
DE                  14.47 usec
TE                  298.0 K
D1             1.00000000 sec
TD0                     1
SFO1          400.1328009 MHz
NUC1                   1H
P1                  15.50 usec
PLW1           9.89999962 W

F2 - Processing parameters
SI                 131072
SF            400.1300097 MHz
WDW                    EM
SSB                     0
LB                      0 Hz
GB                      0
PC                   1.00
```

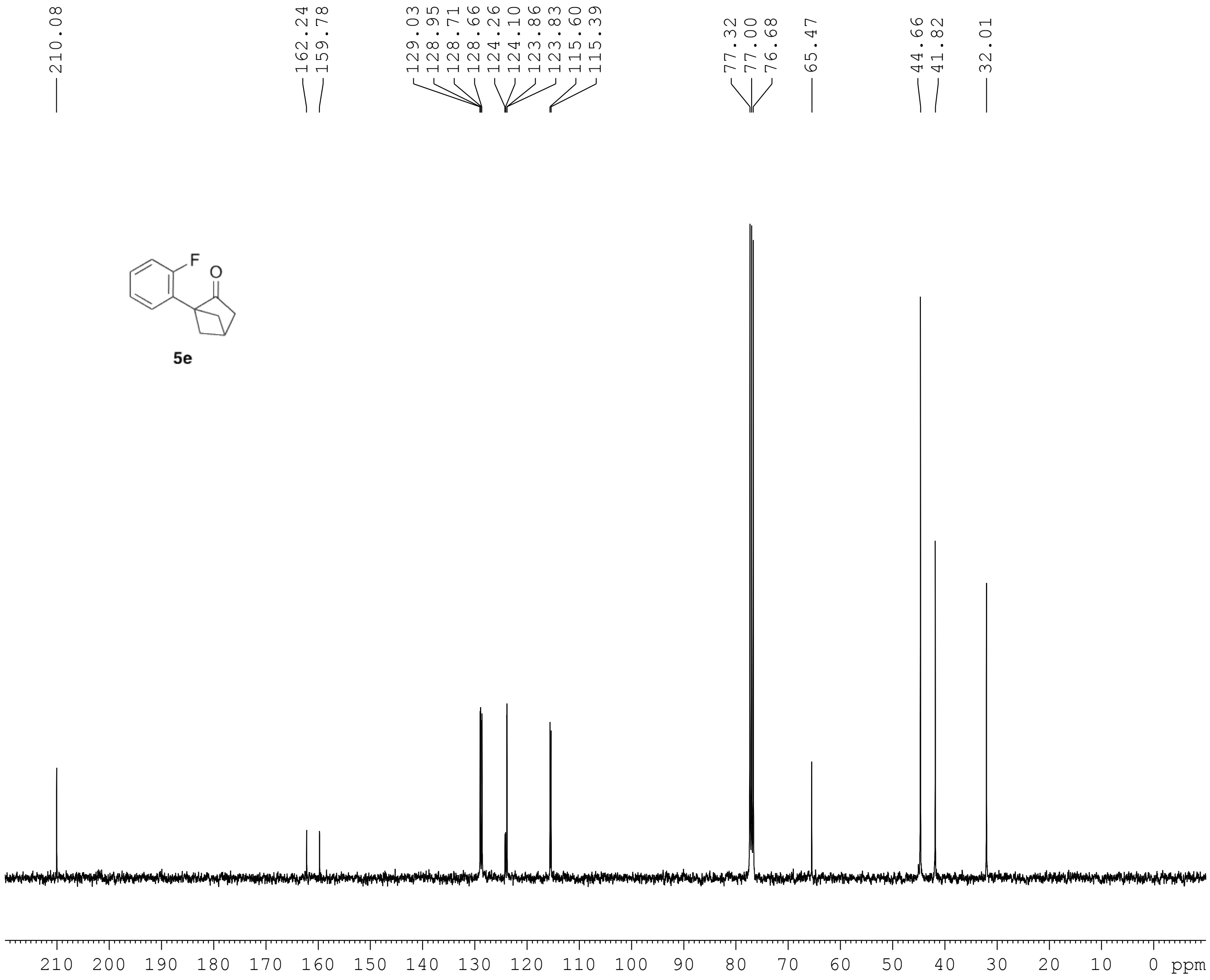


```
Current Data Parameters
NAME                R 2F C
EXPNO                    7
PROCNO                   1

F2 - Acquisition Parameters
Date_             20240302
Time                 11.04 h
INSTRUM              spect
PROBHD   Z108618_0411 (
PULPROG            zgpg30
TD                   65536
SOLVENT              CDCl3
NS                     250
DS                       0
SWH              28409.092 Hz
FIDRES            0.866977 Hz
AQ               1.1534336 sec
RG                  212.49
DW                  17.600 usec
DE                    6.50 usec
TE                   298.1 K
D1              2.00000000 sec
D11             0.03000000 sec
TD0                      1
SFO1        100.6258487 MHz
NUC1                   13C
P1                   10.50 usec
PLW1           42.50000000 W
SFO2        400.1316005 MHz
NUC2                    1H
CPDPRG[2           waltz16
PCPD2                90.00 usec
PLW2            9.89999962 W
PLW12           0.29363999 W
PLW13           0.14747000 W

F2 - Processing parameters
SI                   32768
SF          100.6127739 MHz
WDW                     EM
SSB                      0
LB                    3.00 Hz
GB                       0
PC                    1.40
```

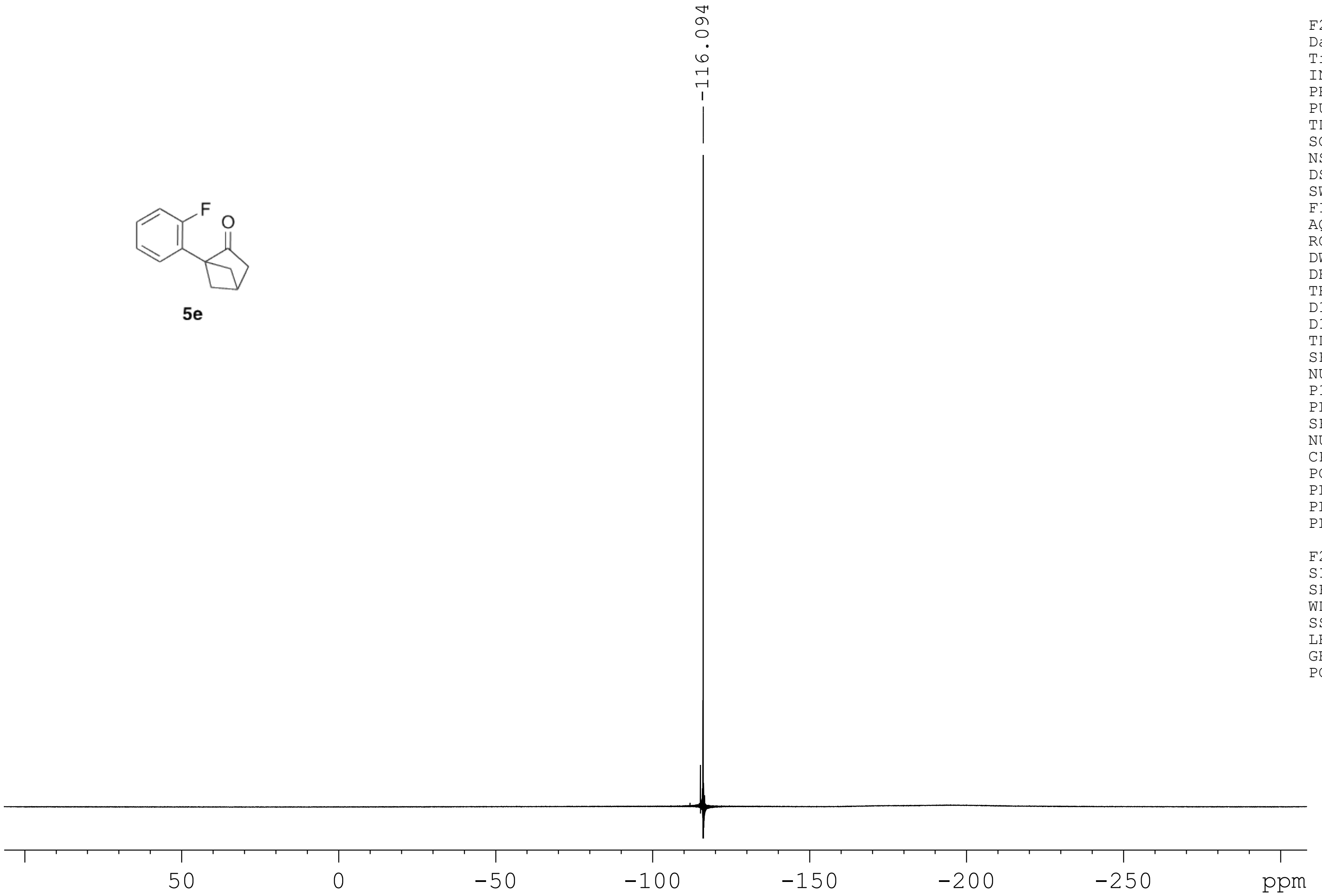


Current Data Parameters
NAME 20240808_R_2F
EXPNO 1
PROCNO 1

F2 - Acquisition Parameters
Date_ 20240808
Time 16.46 h
INSTRUM spect
PROBHD Z108618_0411 (
PULPROG zgpg30
TD 65536
SOLVENT CDCl3
NS 14
DS 2
SWH 156250.000 Hz
FIDRES 4.768372 Hz
AQ 0.2097152 sec
RG 212.49
DW 3.200 usec
DE 6.92 usec
TE 298.1 K
D1 1.50000000 sec
D11 0.03000000 sec
TD0 1
SFO1 376.4607162 MHz
NUC1 19F
P1 15.50 usec
PLW1 11.89999962 W
SFO2 400.1316005 MHz
NUC2 1H
CPDPRG[2 garp4
PCPD2 90.00 usec
PLW2 9.89999962 W
PLW12 0.29363999 W
PLW13 0.14747000 W

F2 - Processing parameters
SI 131072
SF 376.4987069 MHz
WDW EM
SSB 0
LB 0 Hz
GB 0
PC 1.00

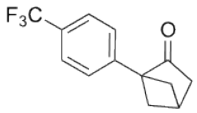
F3C
O

**5f**

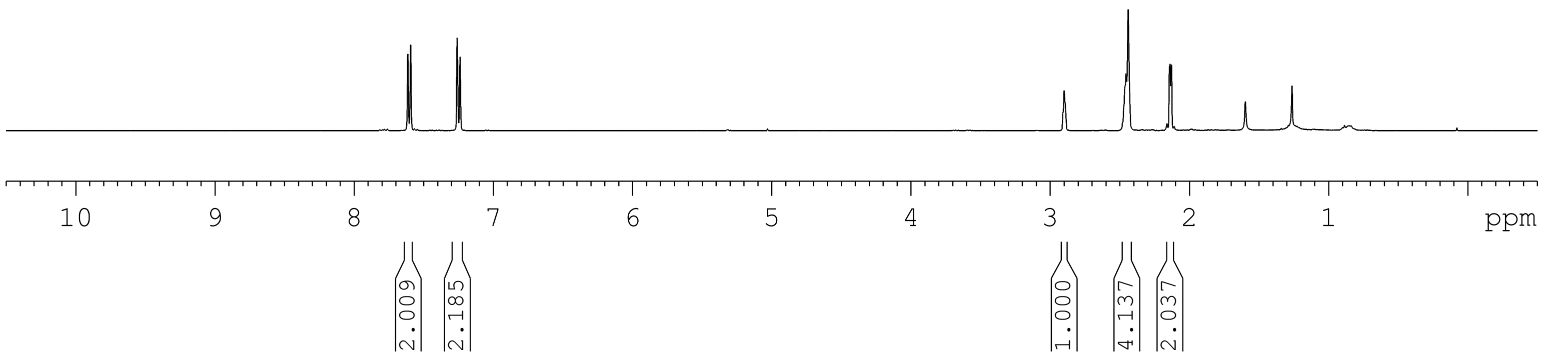
7.615
7.595
7.262
7.260
7.240
2.913
2.910
2.905
2.901
2.893
2.889
2.886
2.456
2.440
2.144
2.140
2.133
2.129
Current Data Parameters
NAME 20240503-4Cf3 HNMR
EXPNO 5
PROCNO 1
F2 - Acquisition Parameters
Date_ 20240503
Time 10.11 h
INSTRUM spect
PROBHD Z108618_0411 (
PULPROG zg30
TD 32768
SOLVENT CDCl3
NS 8
DS 0
SWH 8802.817 Hz
FIDRES 0.537281 Hz
AQ 1.8612224 sec
RG 118.08
DW 56.800 usec
DE 14.47 usec
TE 298.0 K
D1 1.00000000 sec
TD0 1
SFO1 400.1328009 MHz
NUC1 1H
P1 15.50 usec
PLW1 9.89999962 W
F2 - Processing parameters
SI 131072
SF 400.1300082 MHz
WDW EM
SSB 0
LB 0 Hz
GB 0
PC 1.00
10
9
8
7
6
5
4
3
2
1
ppm
2.009
2.185
1.000
4.137
2.037

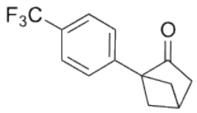

**5f**

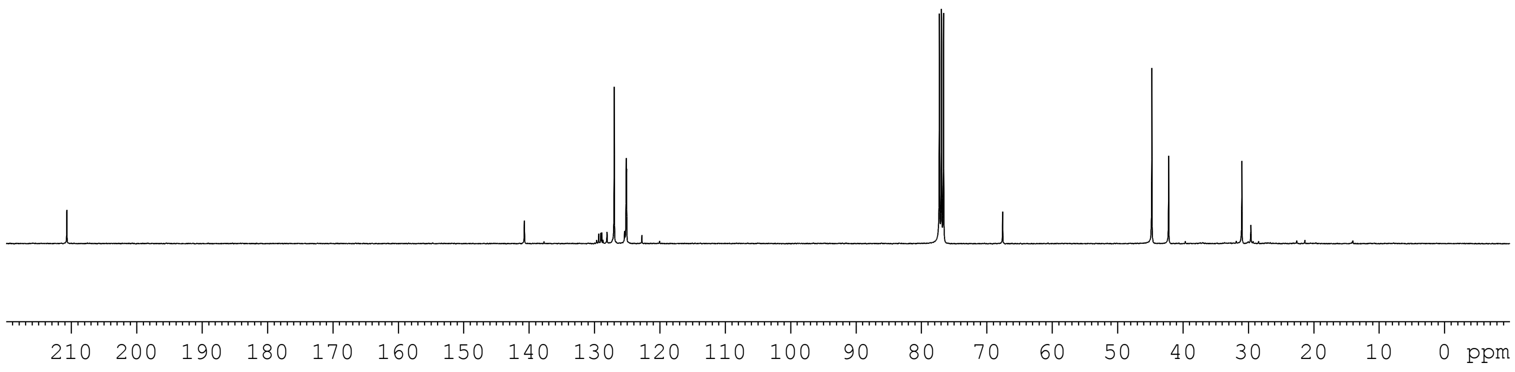


```
Current Data Parameters
NAME     ring 4cf3 CNMR
EXPNO                 1
PROCNO                1

F2 - Acquisition Parameters
Date_          20240808
Time               7.58 h
INSTRUM           spect
PROBHD   Z108618_0411 (
PULPROG         zgpg30
TD                65536
SOLVENT           CDCl3
NS                10934
DS                    0
SWH           28409.092 Hz
FIDRES         0.866977 Hz
AQ            1.1534336 sec
RG               212.49
DW               17.600 usec
DE                 6.50 usec
TE                298.1 K
D1           2.00000000 sec
D11          0.03000000 sec
TD0                   1
SFO1        100.6258487 MHz
NUC1                13C
P1                10.50 usec
PLW1        42.50000000 W
SFO2        400.1316005 MHz
NUC2                 1H
CPDPRG[2        waltz16
PCPD2             90.00 usec
PLW2         9.89999962 W
PLW12        0.29363999 W
PLW13        0.14747000 W

F2 - Processing parameters
SI                32768
SF          100.6127792 MHz
WDW                  EM
SSB                   0
LB                 3.00 Hz
GB                    0
PC                 1.40
```

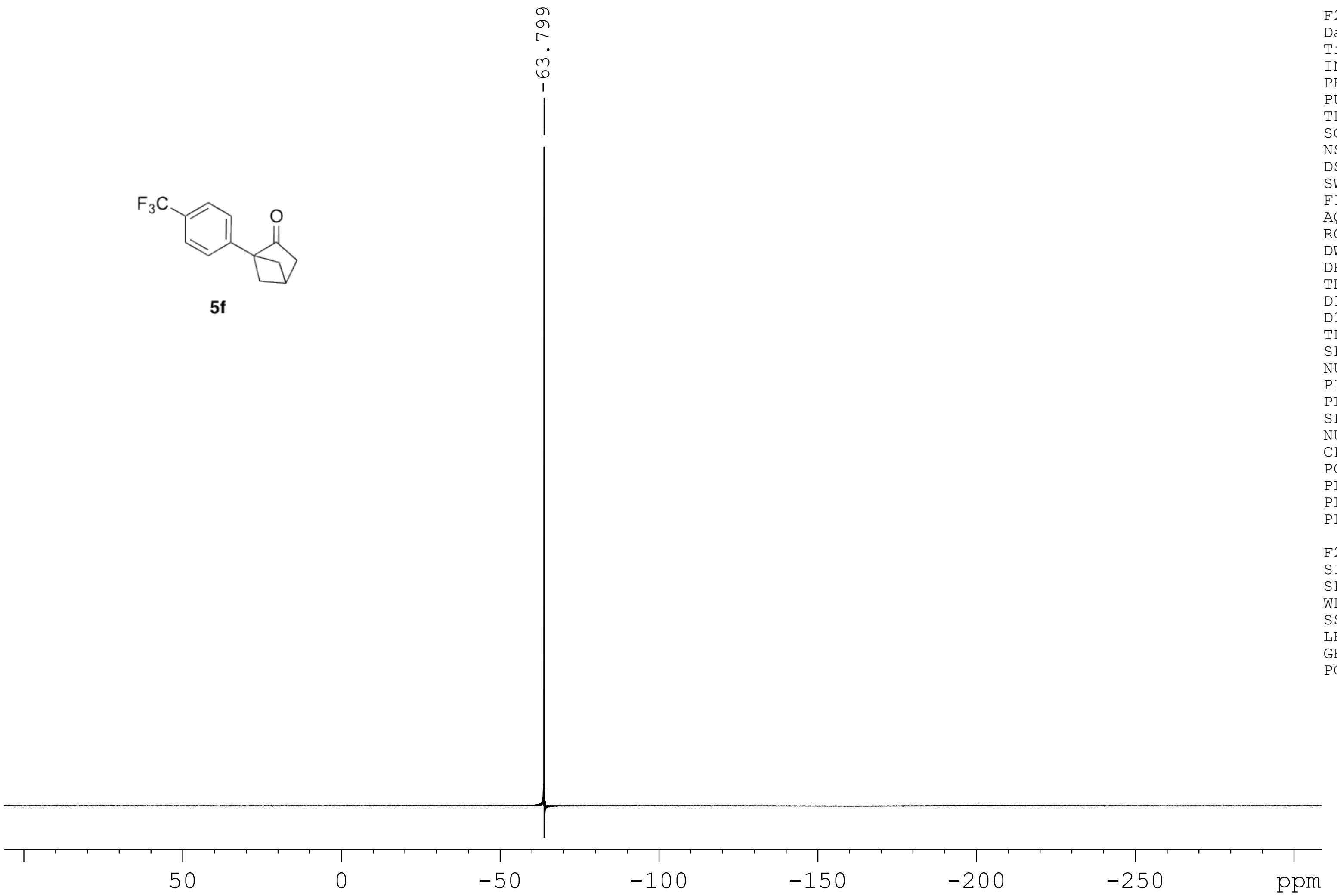


```
Current Data Parameters
NAME      20240808_R_4CF3
EXPNO                  1
PROCNO                 1

F2 - Acquisition Parameters
Date_           20240809
Time               10.44 h
INSTRUM            spect
PROBHD   Z108618_0411 (
PULPROG          zgpg30
TD                 65536
SOLVENT            CDCl3
NS                    12
DS                     2
SWH           156250.000 Hz
FIDRES          4.768372 Hz
AQ             0.2097152 sec
RG                212.49
DW                 3.200 usec
DE                  6.92 usec
TE                 298.1 K
D1            1.50000000 sec
D11           0.03000000 sec
TD0                    1
SFO1         376.4607162 MHz
NUC1                 19F
P1                 15.50 usec
PLW1         11.89999962 W
SFO2         400.1316005 MHz
NUC2                  1H
CPDPRG[2           garp4
PCPD2              90.00 usec
PLW2          9.89999962 W
PLW12         0.29363999 W
PLW13         0.14747000 W

F2 - Processing parameters
SI                131072
SF           376.4988347 MHz
WDW                   EM
SSB                    0
LB                     0 Hz
GB                     0
PC                  1.00
```

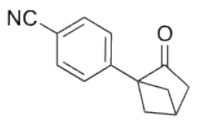

**5g**

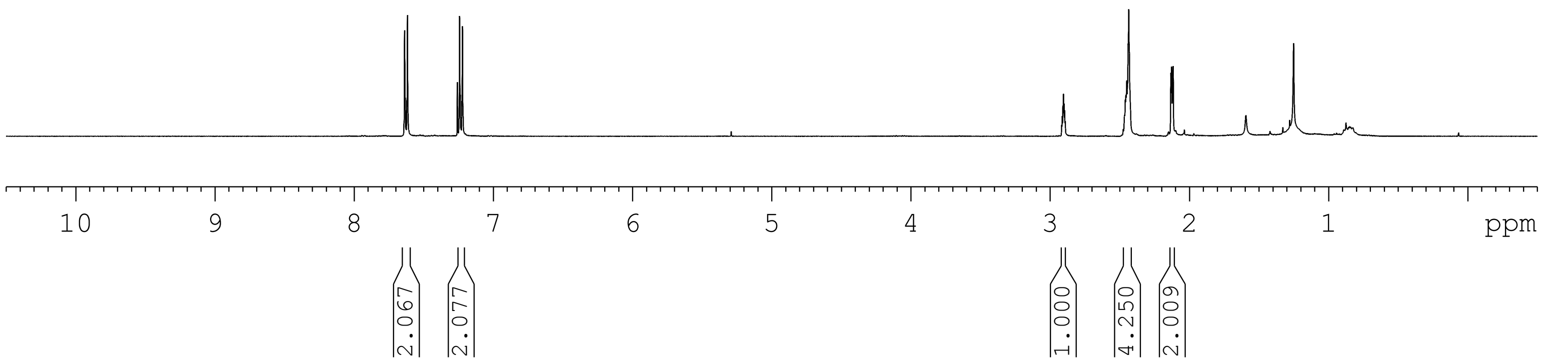


Current Data Parameters
NAME R 4CN H
EXPNO 1
PROCNO 1

F2 - Acquisition Parameters
Date_ 20240327
Time 18.36 h
INSTRUM spect
PROBHD Z108618_0411 (
PULPROG zg30
TD 32768
SOLVENT CDCl3
NS 10
DS 0
SWH 8802.817 Hz
FIDRES 0.537281 Hz
AQ 1.8612224 sec
RG 143.54
DW 56.800 usec
DE 14.47 usec
TE 298.0 K
D1 1.00000000 sec
TD0 1
SFO1 400.1328009 MHz
NUC1 1H
P1 15.50 usec
PLW1 9.89999962 W

F2 - Processing parameters
SI 131072
SF 400.1300097 MHz
WDW EM
SSB 0
LB 0 Hz
GB 0
PC 1.00

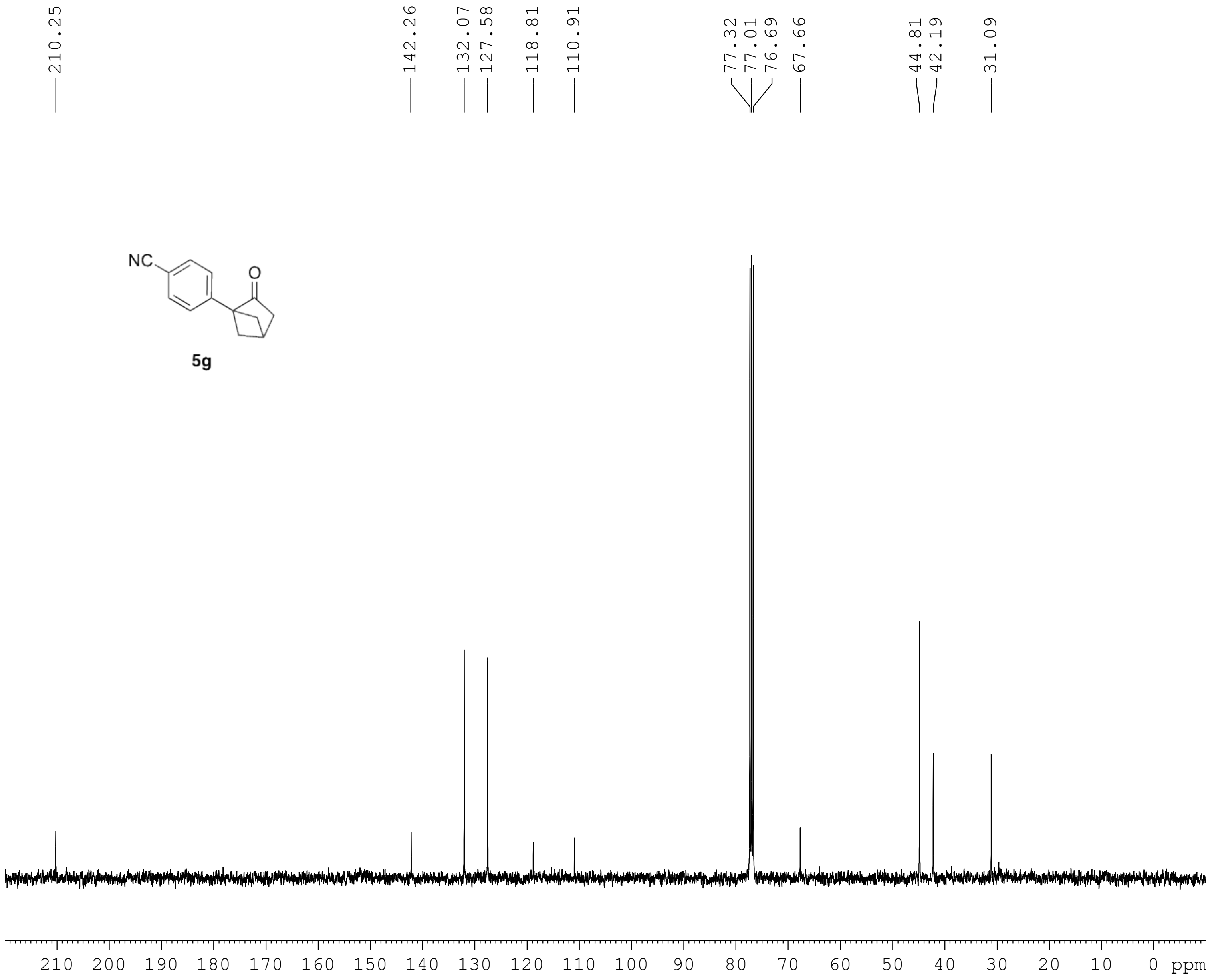


Current Data Parameters
NAME R 4CN C
EXPNO 2
PROCNO 1

F2 - Acquisition Parameters
Date_ 20240328
Time 17.56 h
INSTRUM spect
PROBHD Z108618_0411 (
PULPROG zgpg30
TD 65536
SOLVENT CDCl3
NS 87
DS 4
SWH 28409.092 Hz
FIDRES 0.866977 Hz
AQ 1.1534336 sec
RG 212.49
DW 17.600 usec
DE 6.50 usec
TE 298.1 K
D1 2.00000000 sec
D11 0.03000000 sec
TD0 1
SFO1 100.6258487 MHz
NUC1 13C
P1 10.50 usec
PLW1 42.50000000 W
SFO2 400.1316005 MHz
NUC2 1H
CPDPRG[2 waltz16
PCPD2 90.00 usec
PLW2 9.89999962 W
PLW12 0.29363999 W
PLW13 0.14747000 W

F2 - Processing parameters
SI 32768
SF 100.6127725 MHz
WDW EM
SSB 0
LB 3.00 Hz
GB 0
PC 1.40

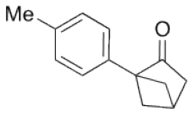


**5h**

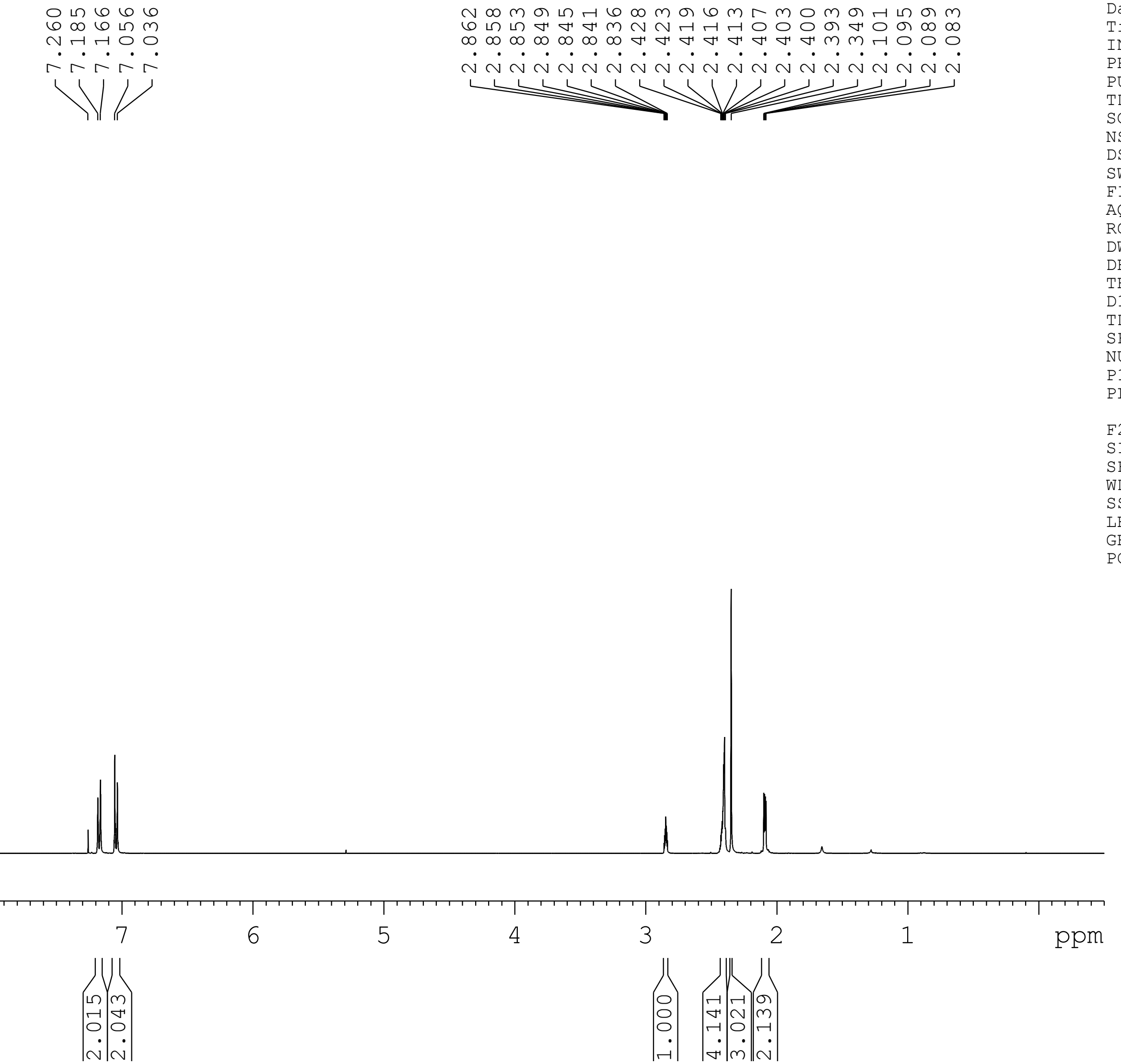


```
Current Data Parameters
NAME     20240220_Rearrange_4Me
EXPNO                     1
PROCNO                    1

F2 - Acquisition Parameters
Date_              20240220
Time                  17.43 h
INSTRUM               spect
PROBHD    Z108618_0411 (
PULPROG                zg30
TD                    32768
SOLVENT               CDCl3
NS                       25
DS                        0
SWH               8802.817 Hz
FIDRES            0.537281 Hz
AQ               1.8612224 sec
RG                    62.98
DW                   56.800 usec
DE                    14.47 usec
TE                    298.0 K
D1              1.00000000 sec
TD0                       1
SFO1          400.1328009 MHz
NUC1                     1H
P1                    15.50 usec
PLW1            9.89999962 W

F2 - Processing parameters
SI                   131072
SF            400.1300097 MHz
WDW                      EM
SSB                       0
LB                        0 Hz
GB                        0
PC                     1.00
```

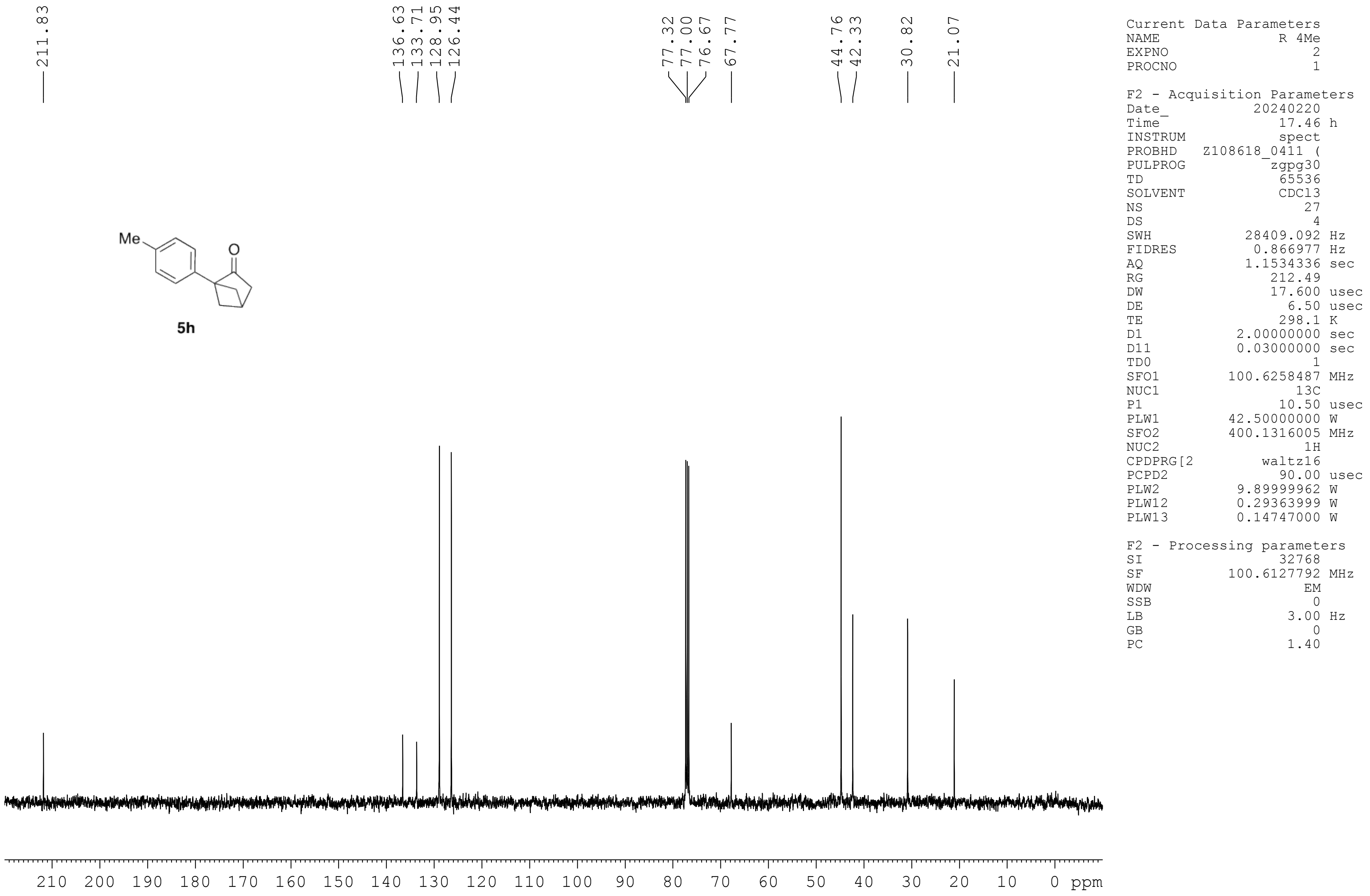
211.83
136.63
133.71
128.95
126.44
77.32
77.00
76.67
67.77
44.76
42.33
30.82
21.07
Me
O
5h
210 200 190 180 170 160 150 140 130 120 110 100 90 80 70 60 50 40 30 20 10 0 ppm
Current Data Parameters
NAME R 4Me
EXPNO 2
PROCNO 1
F2 - Acquisition Parameters
Date_ 20240220
Time 17.46 h
INSTRUM spect
PROBHD Z108618_0411 (
PULPROG zgpg30
TD 65536
SOLVENT CDCl3
NS 27
DS 4
SWH 28409.092 Hz
FIDRES 0.866977 Hz
AQ 1.1534336 sec
RG 212.49
DW 17.600 usec
DE 6.50 usec
TE 298.1 K
D1 2.00000000 sec
D11 0.03000000 sec
TD0 1
SFO1 100.6258487 MHz
NUC1 13C
P1 10.50 usec
PLW1 42.50000000 W
SFO2 400.1316005 MHz
NUC2 1H
CPDPRG[2 waltz16
PCPD2 90.00 usec
PLW2 9.89999962 W
PLW12 0.29363999 W
PLW13 0.14747000 W
F2 - Processing parameters
SI 32768
SF 100.6127792 MHz
WDW EM
SSB 0
LB 3.00 Hz
GB 0
PC 1.40

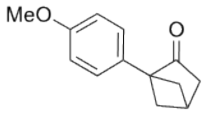

5i

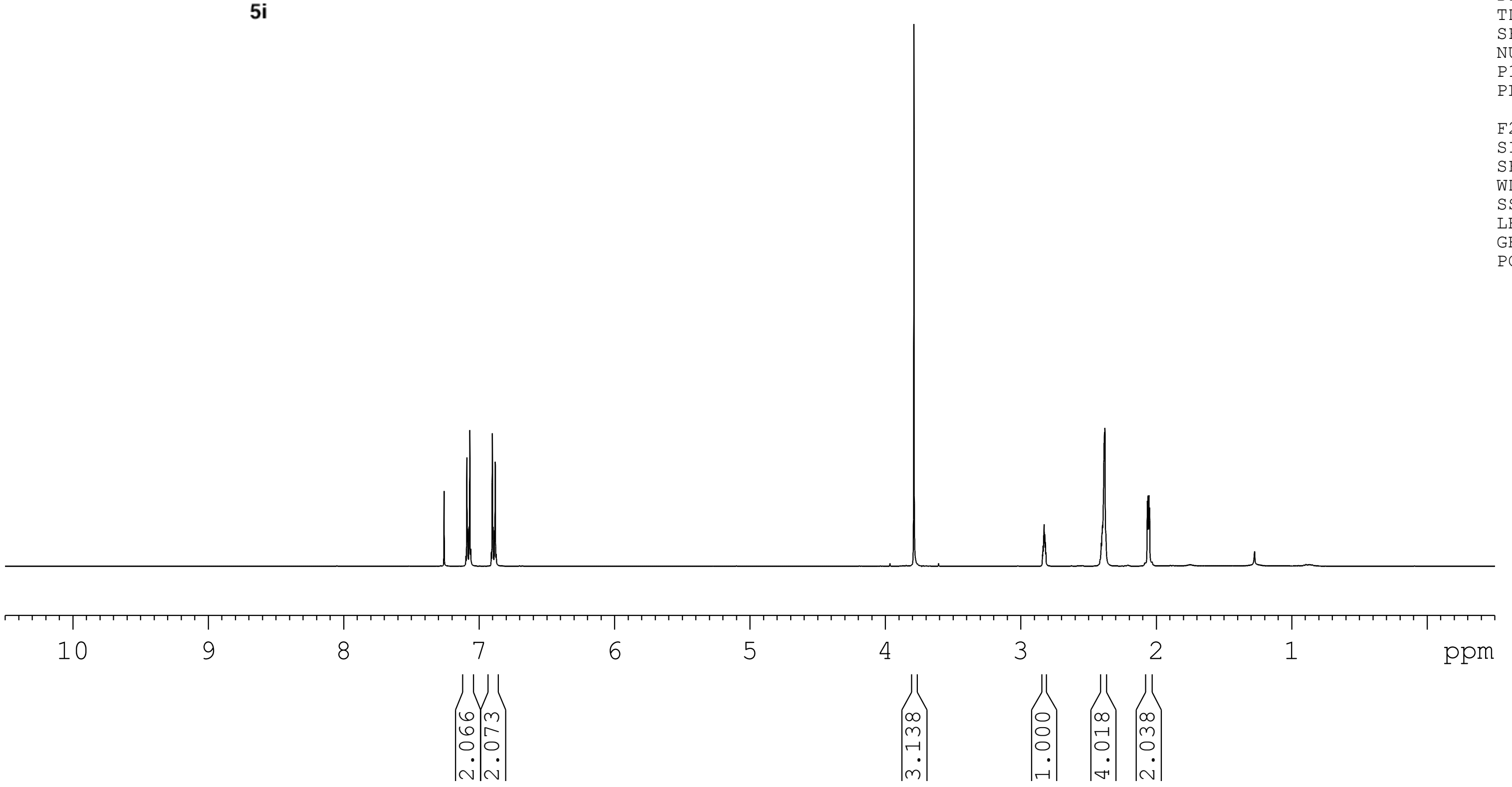


Current Data Parameters
NAME 20240102_Rearrange_4OMe
EXPNO 2
PROCNO 1

F2 - Acquisition Parameters
Date_ 20240103
Time 13.17 h
INSTRUM spect
PROBHD Z108618_0411 (
PULPROG zg30
TD 32768
SOLVENT CDCl3
NS 14
DS 0
SWH 8802.817 Hz
FIDRES 0.537281 Hz
AQ 1.8612224 sec
RG 51.61
DW 56.800 usec
DE 14.47 usec
TE 298.0 K
D1 1.00000000 sec
TD0 1
SFO1 400.1328009 MHz
NUC1 1H
P1 15.50 usec
PLW1 9.89999962 W

F2 - Processing parameters
SI 131072
SF 400.1300094 MHz
WDW EM
SSB 0
LB 0 Hz
GB 0
PC 1.00

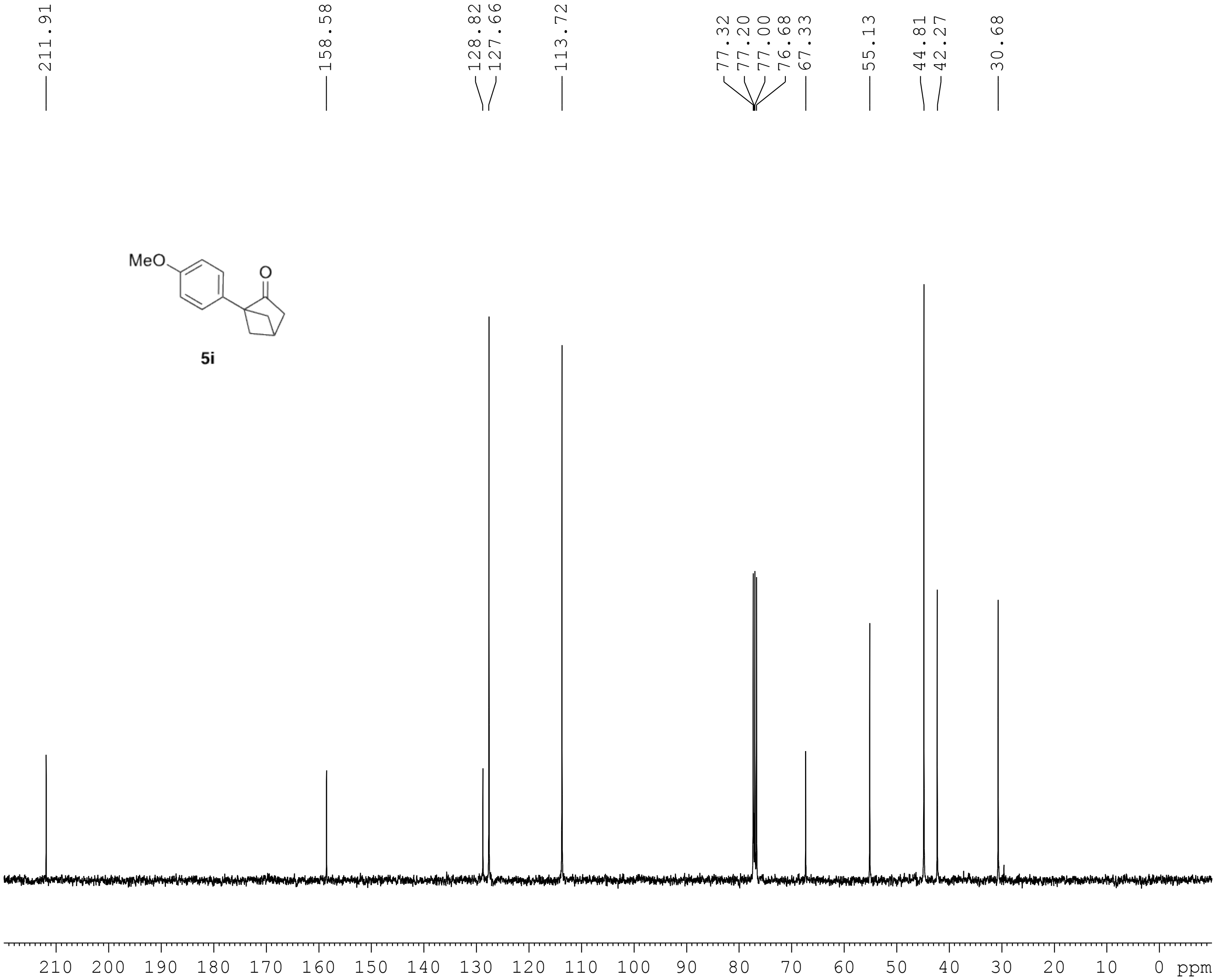


Current Data Parameters
NAME R 4OMe
EXPNO 3
PROCNO 1

F2 - Acquisition Parameters
Date_ 20240103
Time 13.21 h
INSTRUM spect
PROBHD Z108618_0411 (
PULPROG zgpg30
TD 65536
SOLVENT CDCl3
NS 46
DS 4
SWH 28409.092 Hz
FIDRES 0.866977 Hz
AQ 1.1534336 sec
RG 212.49
DW 17.600 usec
DE 6.50 usec
TE 298.1 K
D1 2.00000000 sec
D11 0.03000000 sec
TD0 1
SFO1 100.6258487 MHz
NUC1 13C
P1 10.50 usec
PLW1 42.50000000 W
SFO2 400.1316005 MHz
NUC2 1H
CPDPRG[2 waltz16
PCPD2 90.00 usec
PLW2 9.89999962 W
PLW12 0.29363999 W
PLW13 0.14747000 W

F2 - Processing parameters
SI 32768
SF 100.6127835 MHz
WDW EM
SSB 0
LB 3.00 Hz
GB 0
PC 1.40

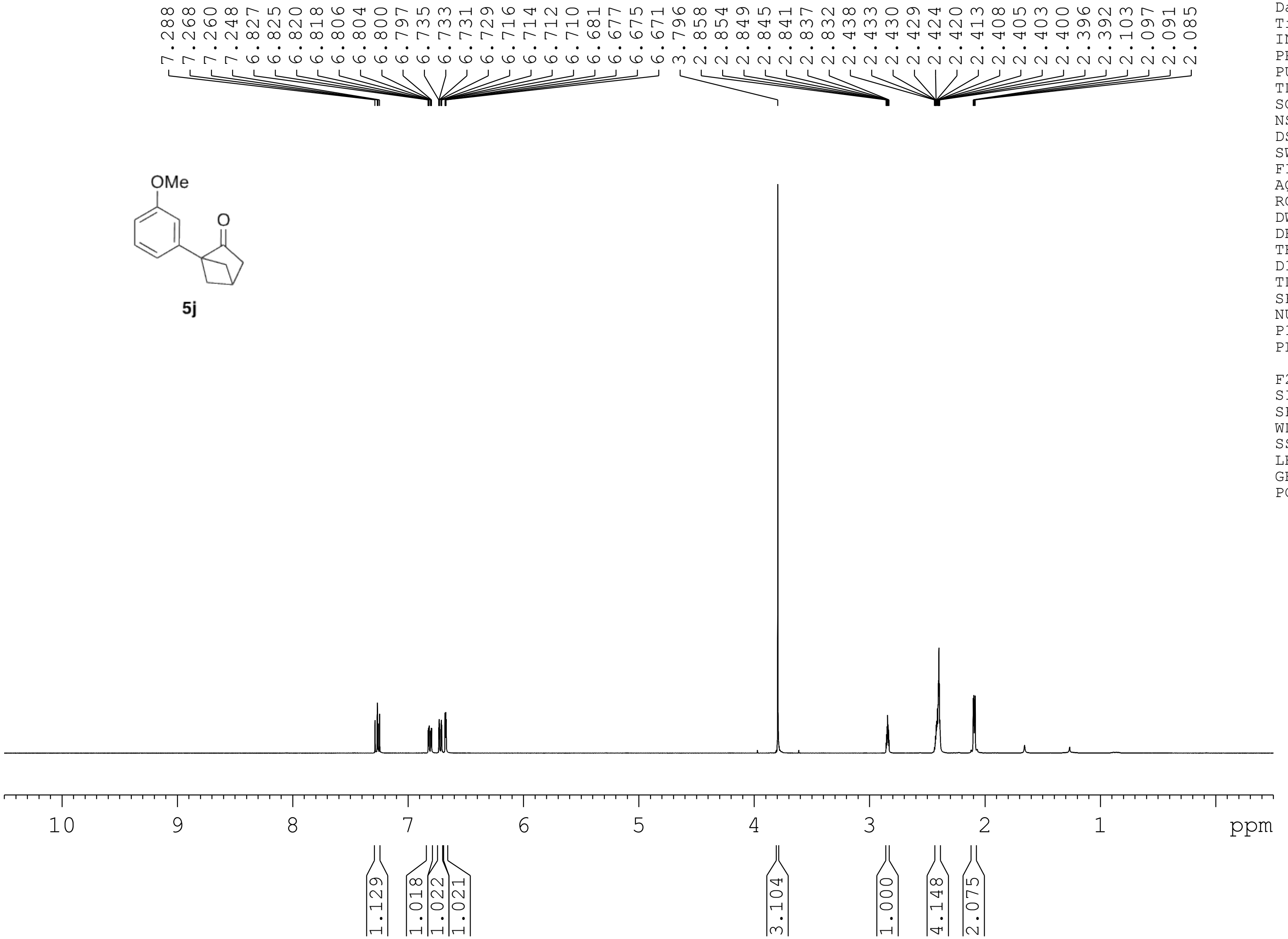


Current Data Parameters
NAME R 3OMe
EXPNO 2
PROCNO 1

F2 - Acquisition Parameters
Date_ 20240131
Time 16.57 h
INSTRUM spect
PROBHD Z108618_0411 (
PULPROG zg30
TD 32768
SOLVENT CDCl3
NS 18
DS 0
SWH 8802.817 Hz
FIDRES 0.537281 Hz
AQ 1.8612224 sec
RG 71.05
DW 56.800 usec
DE 14.47 usec
TE 298.0 K
D1 1.00000000 sec
TD0 1
SFO1 400.1328009 MHz
NUC1 1H
P1 15.50 usec
PLW1 9.89999962 W

F2 - Processing parameters
SI 131072
SF 400.1300096 MHz
WDW EM
SSB 0
LB 0 Hz
GB 0
PC 1.00

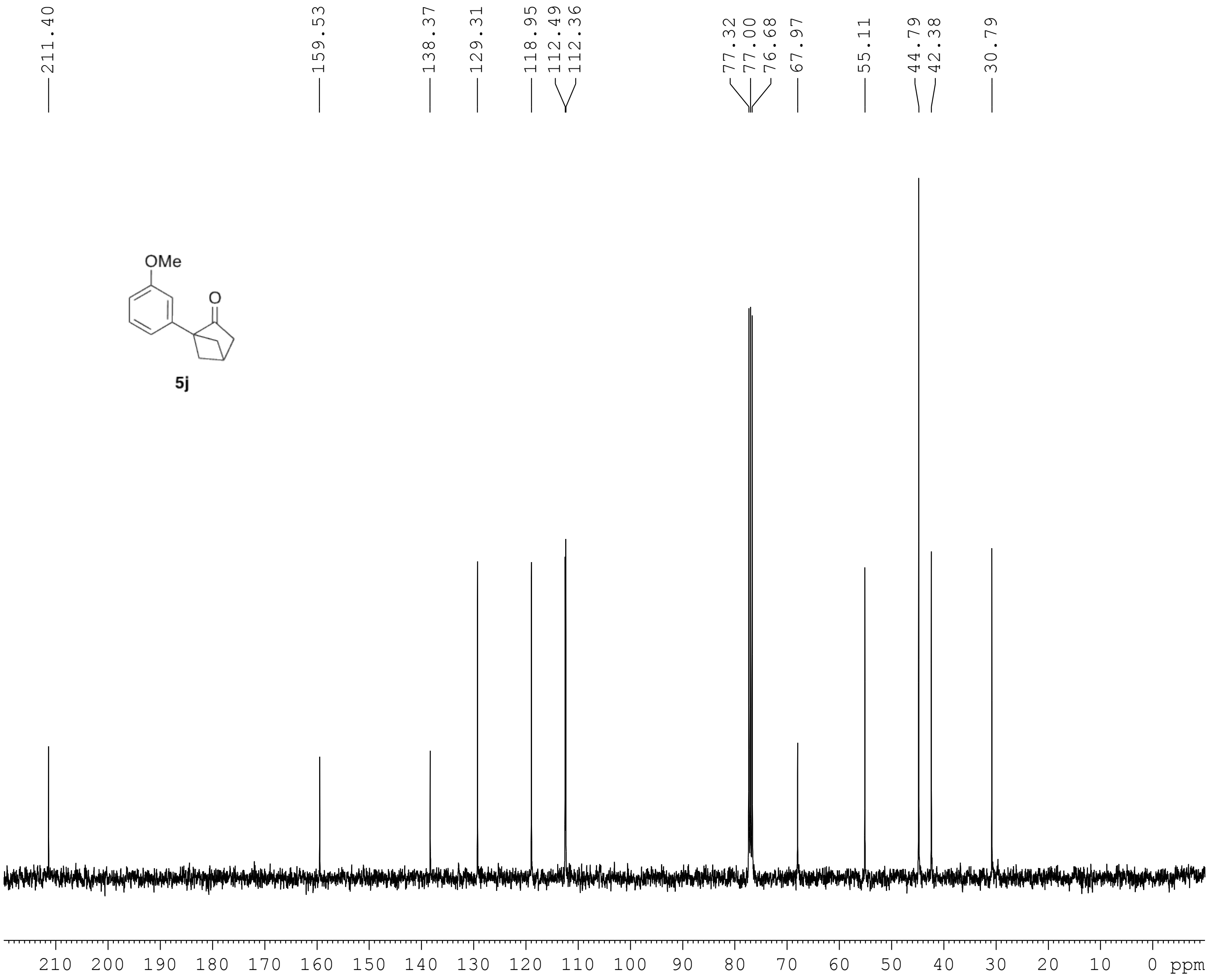

5j



```
Current Data Parameters
NAME                R 3OMe
EXPNO                    3
PROCNO                   1

F2 - Acquisition Parameters
Date_             20240131
Time                 17.00 h
INSTRUM              spect
PROBHD   Z108618_0411 (
PULPROG            zgpg30
TD                   65536
SOLVENT              CDCl3
NS                      43
DS                       4
SWH              28409.092 Hz
FIDRES            0.866977 Hz
AQ               1.1534336 sec
RG                  212.49
DW                  17.600 usec
DE                    6.50 usec
TE                   298.1 K
D1              2.00000000 sec
D11             0.03000000 sec
TD0                      1
SFO1        100.6258487 MHz
NUC1                   13C
P1                   10.50 usec
PLW1           42.50000000 W
SFO2        400.1316005 MHz
NUC2                    1H
CPDPRG[2           waltz16
PCPD2                90.00 usec
PLW2            9.89999962 W
PLW12           0.29363999 W
PLW13           0.14747000 W

F2 - Processing parameters
SI                   32768
SF          100.6127765 MHz
WDW                     EM
SSB                      0
LB                    3.00 Hz
GB                       0
PC                    1.40
```

```
Current Data Parameters
NAME          R 2OMe H
EXPNO                2
PROCNO               1

F2 - Acquisition Parameters
Date_          20240302
Time              10.27 h
INSTRUM           spect
PROBHD   Z108618_0411 (
PULPROG            zg30
TD                32768
SOLVENT           CDCl3
NS                   19
DS                    0
SWH            8802.817 Hz
FIDRES         0.537281 Hz
AQ            1.8612224 sec
RG               118.08
DW               56.800 usec
DE                14.47 usec
TE                298.0 K
D1           1.00000000 sec
TD0                   1
SFO1        400.1328009 MHz
NUC1                 1H
P1                15.50 usec
PLW1         9.89999962 W

F2 - Processing parameters
SI               131072
SF          400.1300094 MHz
WDW                  EM
SSB                   0
LB                    0 Hz
GB                    0
PC                 1.00
```

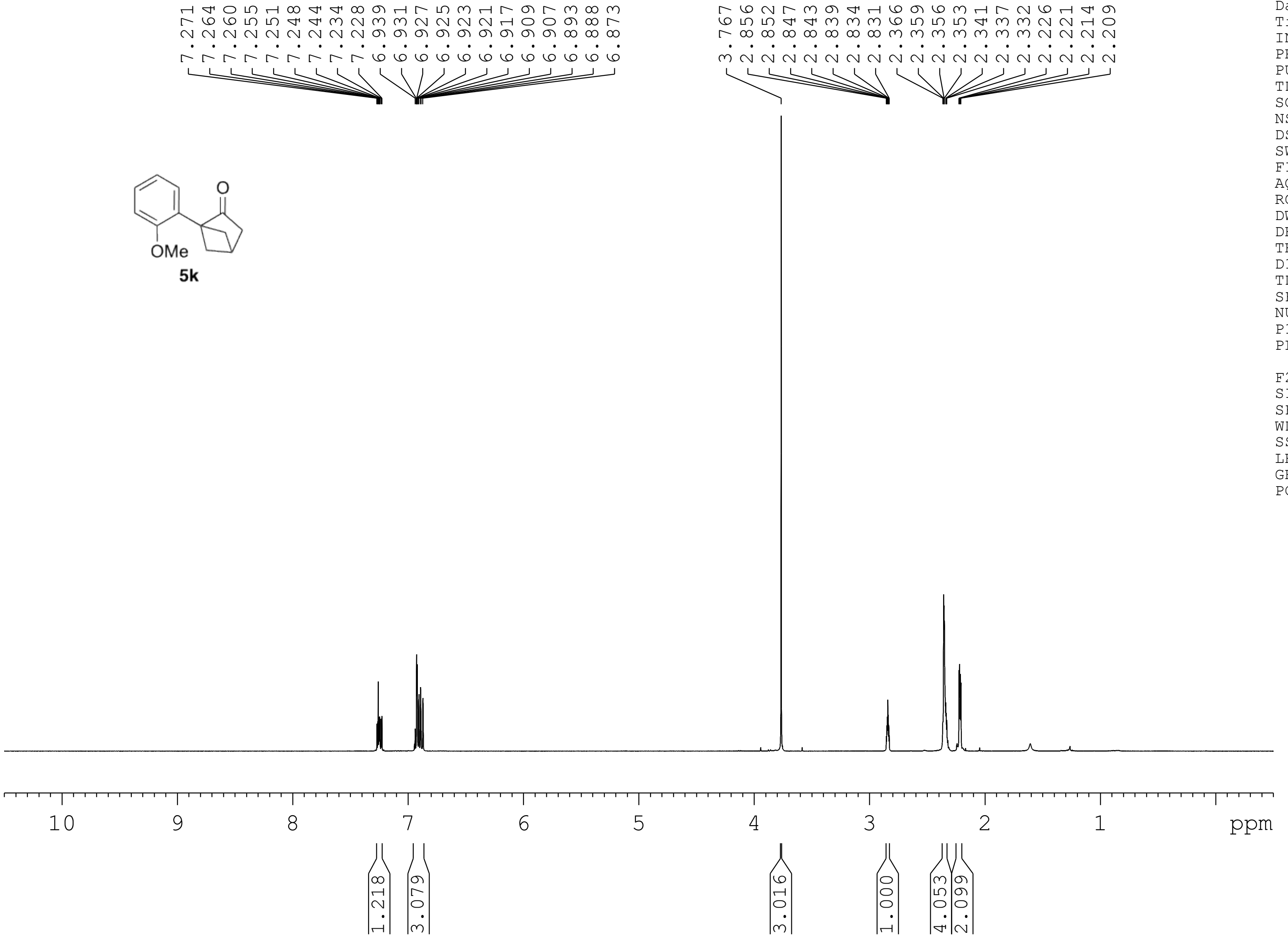

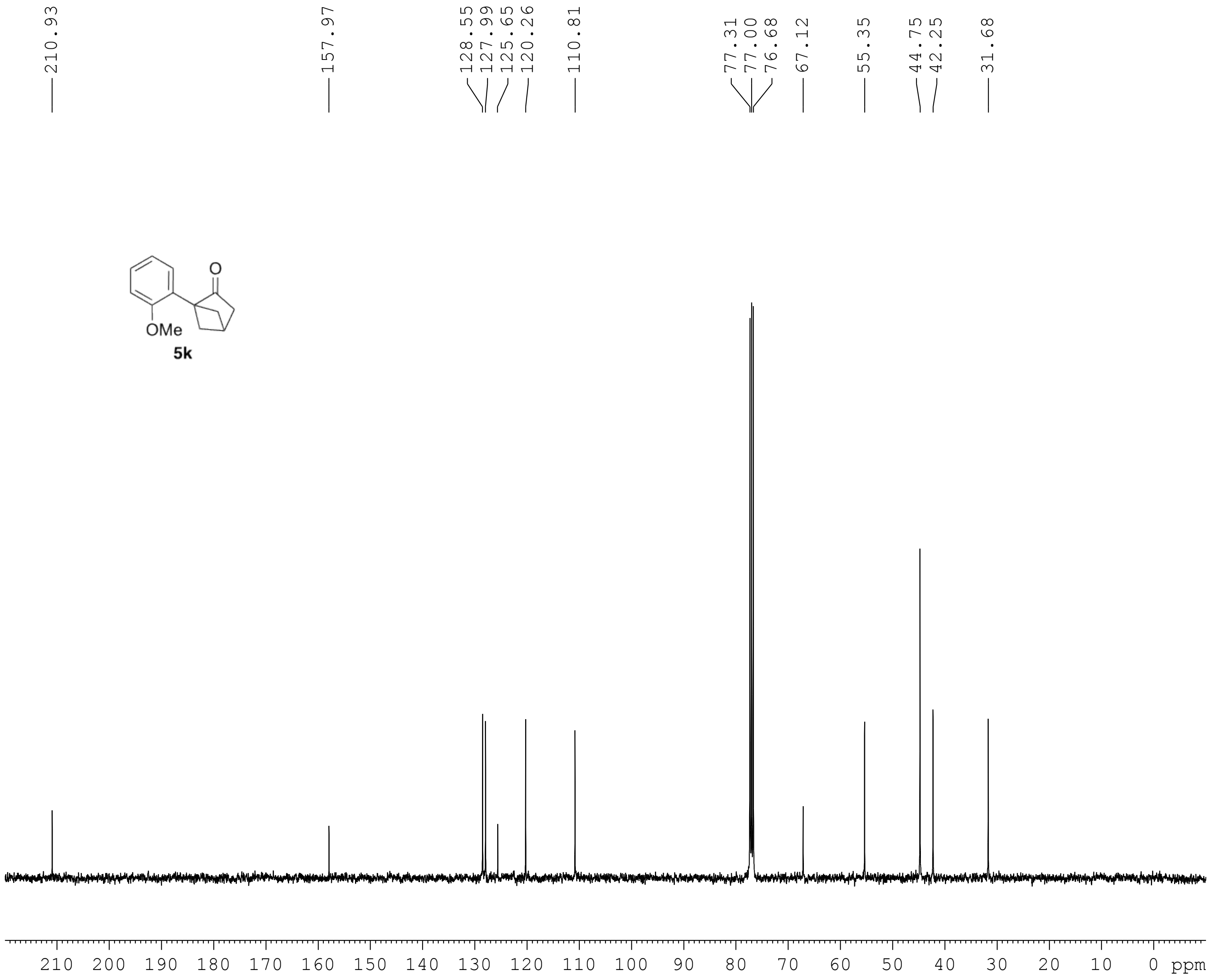


**5k**

```
Current Data Parameters
NAME             R 2OMe C
EXPNO                   3
PROCNO                  1

F2 - Acquisition Parameters
Date_            20240302
Time                10.37 h
INSTRUM             spect
PROBHD   Z108618_0411 (
PULPROG           zgpg30
TD                 65536
SOLVENT            CDCl3
NS                   165
DS                     0
SWH            28409.092 Hz
FIDRES          0.866977 Hz
AQ             1.1534336 sec
RG                212.49
DW                17.600 usec
DE                  6.50 usec
TE                 298.1 K
D1            2.00000000 sec
D11           0.03000000 sec
TD0                    1
SFO1         100.6258487 MHz
NUC1                 13C
P1                 10.50 usec
PLW1         42.50000000 W
SFO2         400.1316005 MHz
NUC2                  1H
CPDPRG[2         waltz16
PCPD2              90.00 usec
PLW2          9.89999962 W
PLW12         0.29363999 W
PLW13         0.14747000 W

F2 - Processing parameters
SI                 32768
SF           100.6127739 MHz
WDW                   EM
SSB                    0
LB                  3.00 Hz
GB                     0
PC                  1.40
```

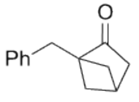


**5n**

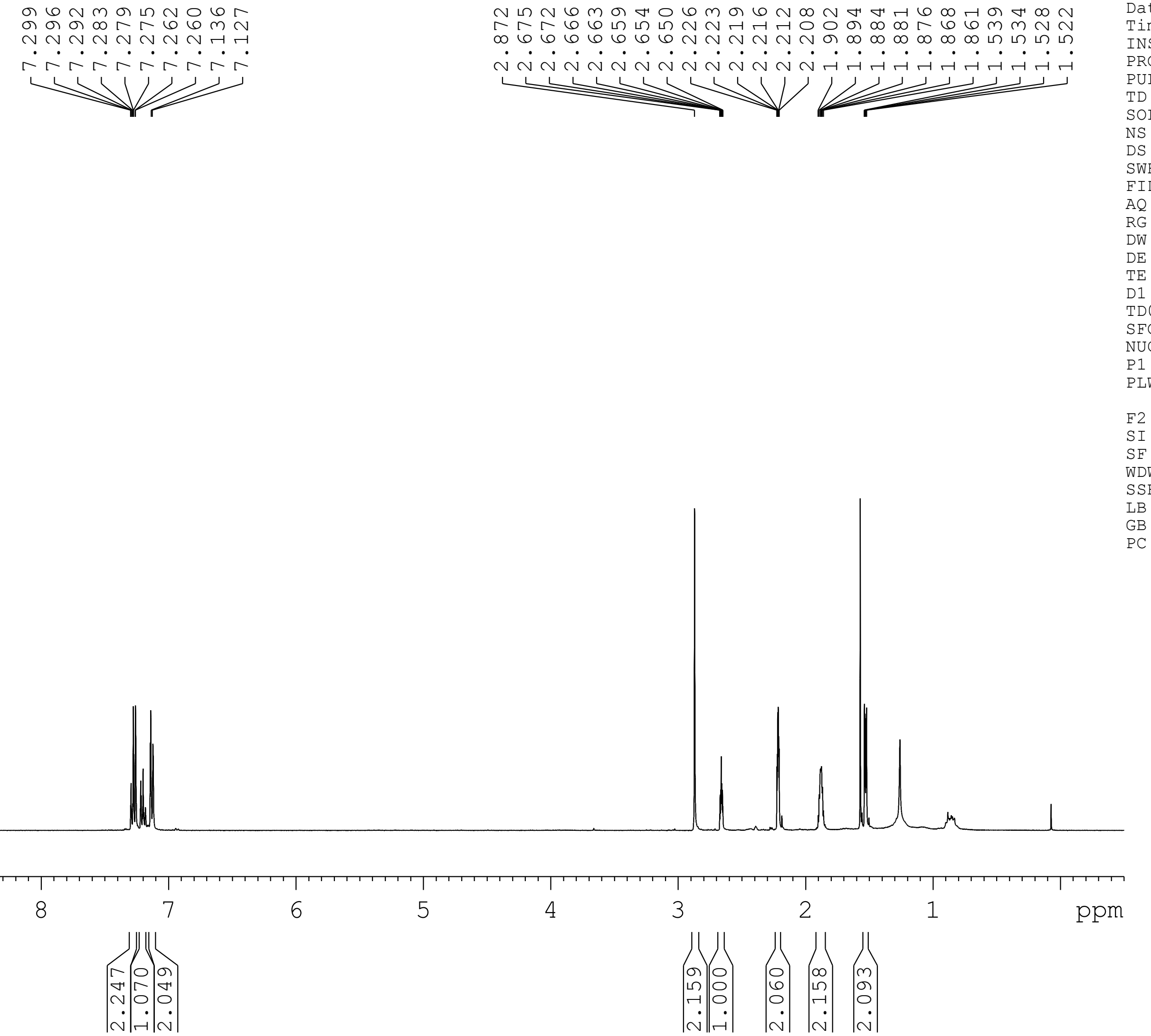


```
Current Data Parameters
NAME     20240621_Rearrange_benzyl
EXPNO                    1
PROCNO                   1

F2 - Acquisition Parameters
Date_            20240621
Time               12.36 h
INSTRUM            spect
PROBHD   Z108618_0411 (
PULPROG             zg30
TD                 32768
SOLVENT            CDCl3
NS                    33
DS                     0
SWH             8802.817 Hz
FIDRES          0.537281 Hz
AQ             1.8612224 sec
RG                 188.2
DW                56.800 usec
DE                 14.47 usec
TE                 298.0 K
D1            1.00000000 sec
TD0                    1
SFO1         400.1328009 MHz
NUC1                  1H
P1                 15.50 usec
PLW1          9.89999962 W

F2 - Processing parameters
SI                131072
SF           400.1300090 MHz
WDW                   EM
SSB                    0
LB                     0 Hz
GB                     0
PC                  1.00
```

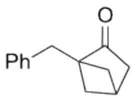

**5n**

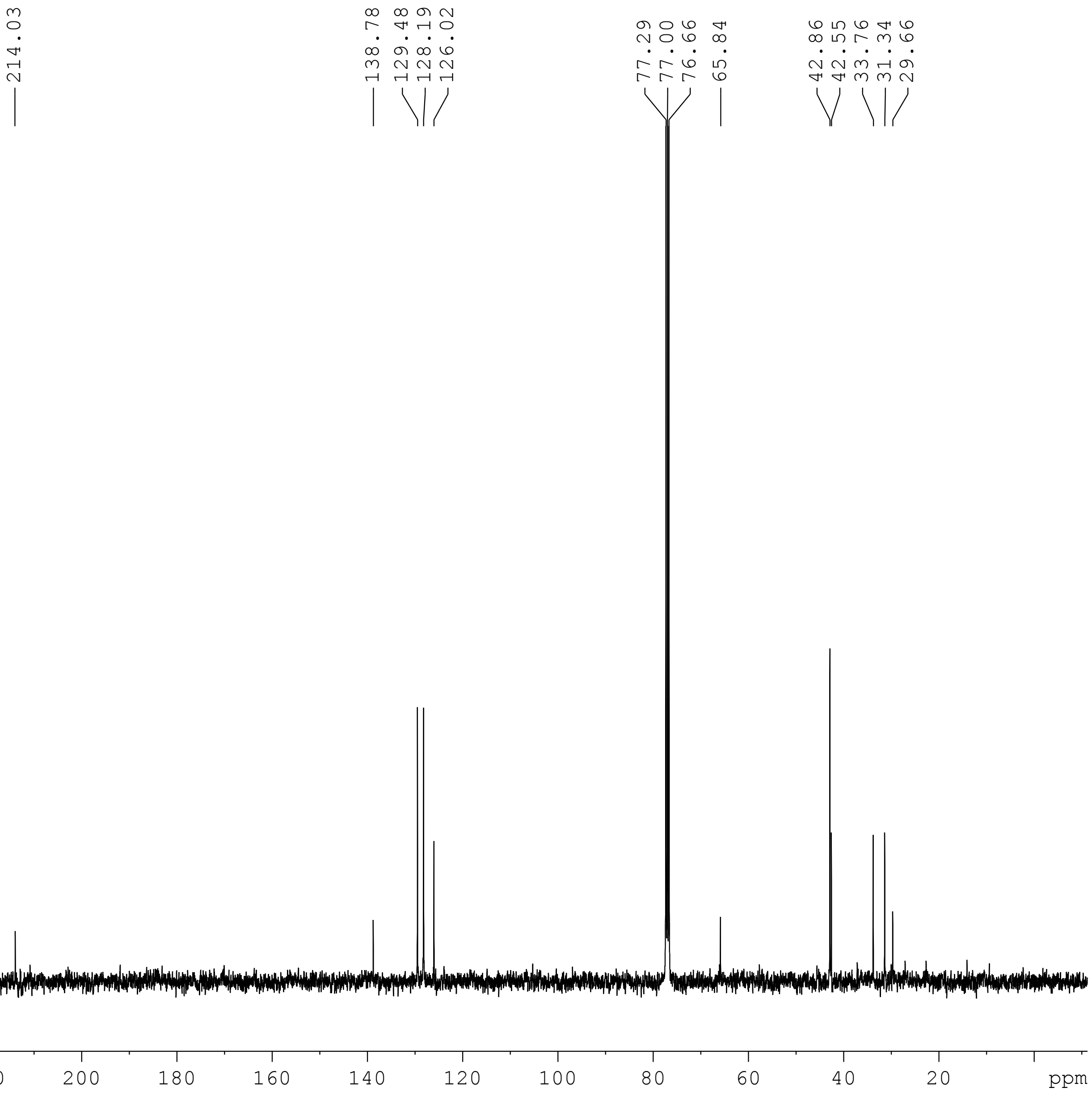


Current Data Parameters
NAME R benzyl CNMR
EXPNO 6
PROCNO 1

F2 - Acquisition Parameters
Date_ 20240623
Time 15.30 h
INSTRUM spect
PROBHD Z108618_0411 (
PULPROG zgpg30
TD 65536
SOLVENT CDCl3
NS 300
DS 0
SWH 28409.092 Hz
FIDRES 0.866977 Hz
AQ 1.1534336 sec
RG 212.49
DW 17.600 usec
DE 6.50 usec
TE 298.1 K
D1 2.00000000 sec
D11 0.03000000 sec
TD0 1
SFO1 100.6258487 MHz
NUC1 13C
P1 10.50 usec
PLW1 42.50000000 W
SFO2 400.1316005 MHz
NUC2 1H
CPDPRG[2 waltz16
PCPD2 90.00 usec
PLW2 9.89999962 W
PLW12 0.29363999 W
PLW13 0.14747000 W

F2 - Processing parameters
SI 32768
SF 100.6127729 MHz
WDW EM
SSB 0
LB 3.00 Hz
GB 0
PC 1.40

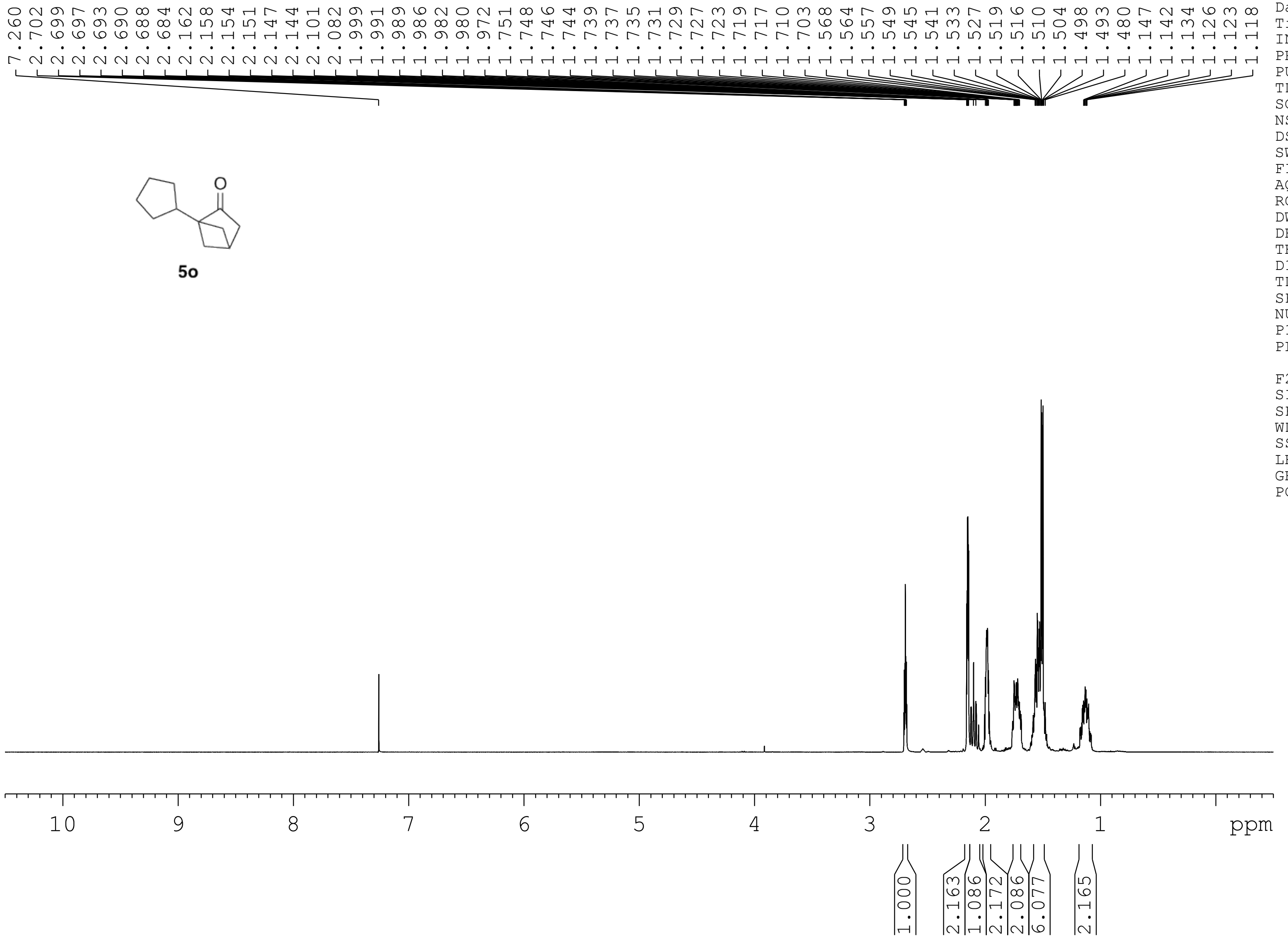


**5o**

```
Current Data Parameters
NAME      R cyclopentane
EXPNO                  1
PROCNO                 1

F2 - Acquisition Parameters
Date_           20240118
Time               17.35 h
INSTRUM            spect
PROBHD   Z108618_0411 (
PULPROG             zg30
TD                 32768
SOLVENT            CDCl3
NS                    53
DS                     0
SWH            8802.817 Hz
FIDRES         0.537281 Hz
AQ            1.8612224 sec
RG                 57.72
DW                56.800 usec
DE                 14.47 usec
TE                 298.0 K
D1            1.00000000 sec
TD0                    1
SFO1        400.1328009 MHz
NUC1                  1H
P1                 15.50 usec
PLW1          9.89999962 W

F2 - Processing parameters
SI                131072
SF          400.1300095 MHz
WDW                   EM
SSB                    0
LB                     0 Hz
GB                     0
PC                  1.00
```

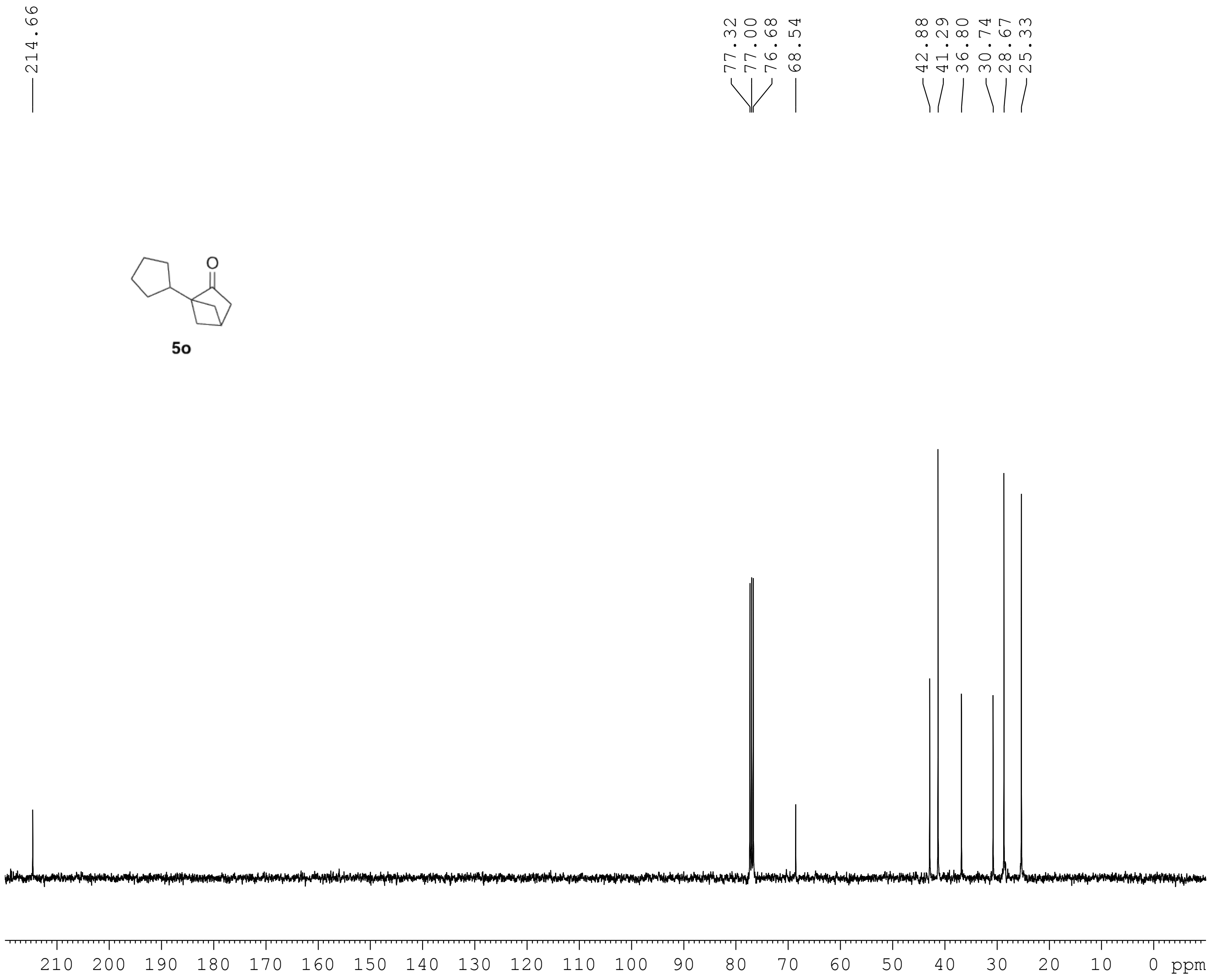

**5o**



```
Current Data Parameters
NAME      R cyclopentane
EXPNO                  2
PROCNO                 1

F2 - Acquisition Parameters
Date_           20240118
Time               17.39 h
INSTRUM            spect
PROBHD   Z108618_0411 (
PULPROG          zgpg30
TD                 65536
SOLVENT            CDCl3
NS                     1
DS                     4
SWH            28409.092 Hz
FIDRES          0.866977 Hz
AQ             1.1534336 sec
RG                212.49
DW                17.600 usec
DE                  6.50 usec
TE                 297.9 K
D1            2.00000000 sec
D11           0.03000000 sec
TD0                    1
SFO1         100.6258487 MHz
NUC1                 13C
P1                 10.50 usec
PLW1         42.50000000 W
SFO2         400.1316005 MHz
NUC2                  1H
CPDPRG[2         waltz16
PCPD2              90.00 usec
PLW2          9.89999962 W
PLW12         0.29363999 W
PLW13         0.14747000 W

F2 - Processing parameters
SI                 32768
SF           100.6127730 MHz
WDW                   EM
SSB                    0
LB                  3.00 Hz
GB                     0
PC                  1.40
```

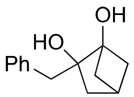


**4n**

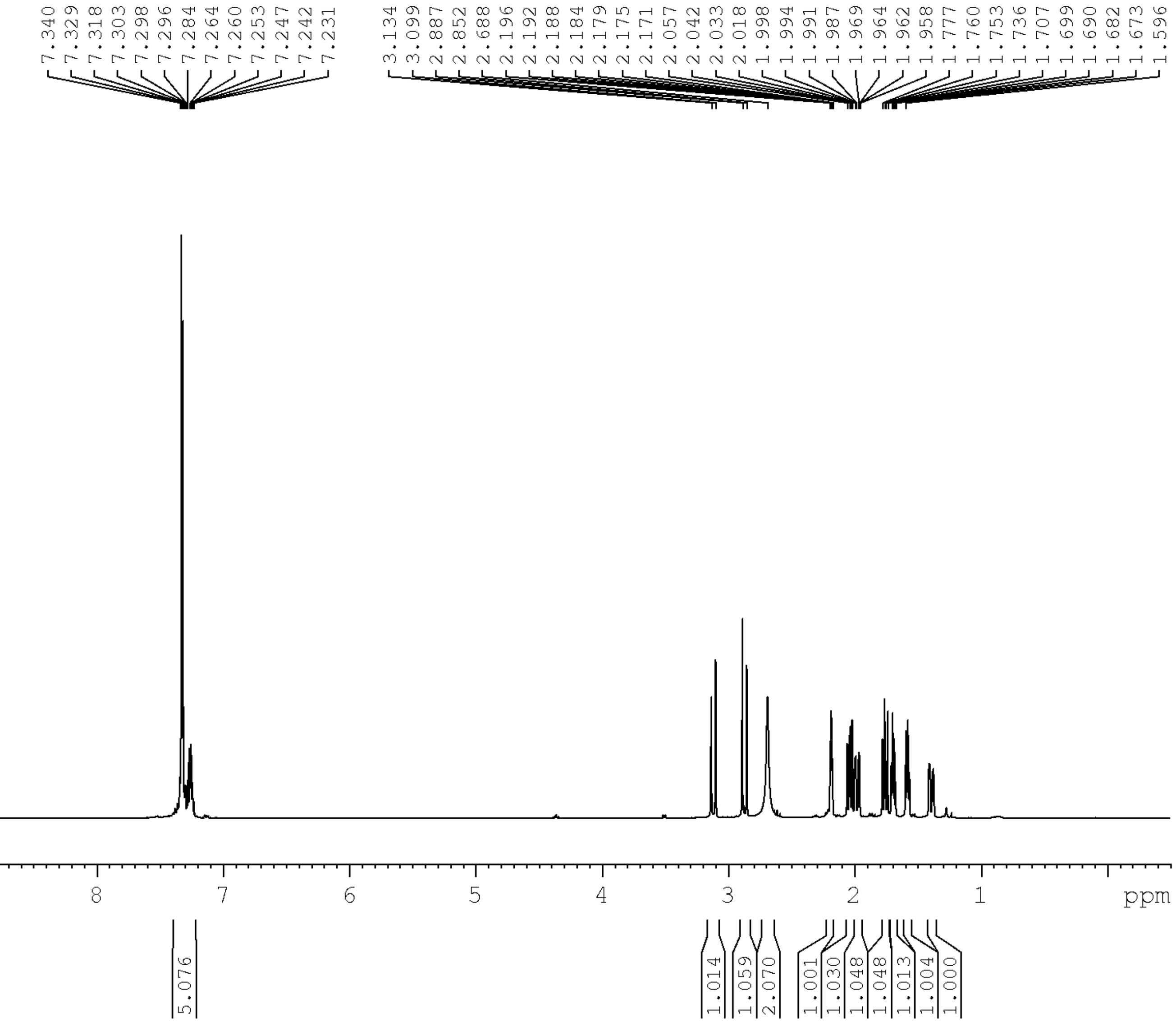


Current Data Parameters
NAME 20241014_deacyl
EXPNO 1
PROCNO 1

F2 - Acquisition Paramet
Date_ 20241014
Time 20.28
INSTRUM spect
PROBHD Z108618_0411 (
PULPROG zg30
TD 32768
SOLVENT CDCl3
NS 30
DS 0
SWH 8802.817
FIDRES 0.537281
AQ 1.8612224
RG 51.61
DW 56.800
DE 14.47
TE 297.9
D1 1.00000000
TD0 1
SFO1 400.1328009
NUC1 1H
P1 15.50
PLW1 9.89999962

F2 - Processing paramete
SI 131072
SF 400.1300099
WDW EM
SSB 0
LB 0
GB 0
PC 1.00

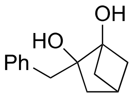


**4n**

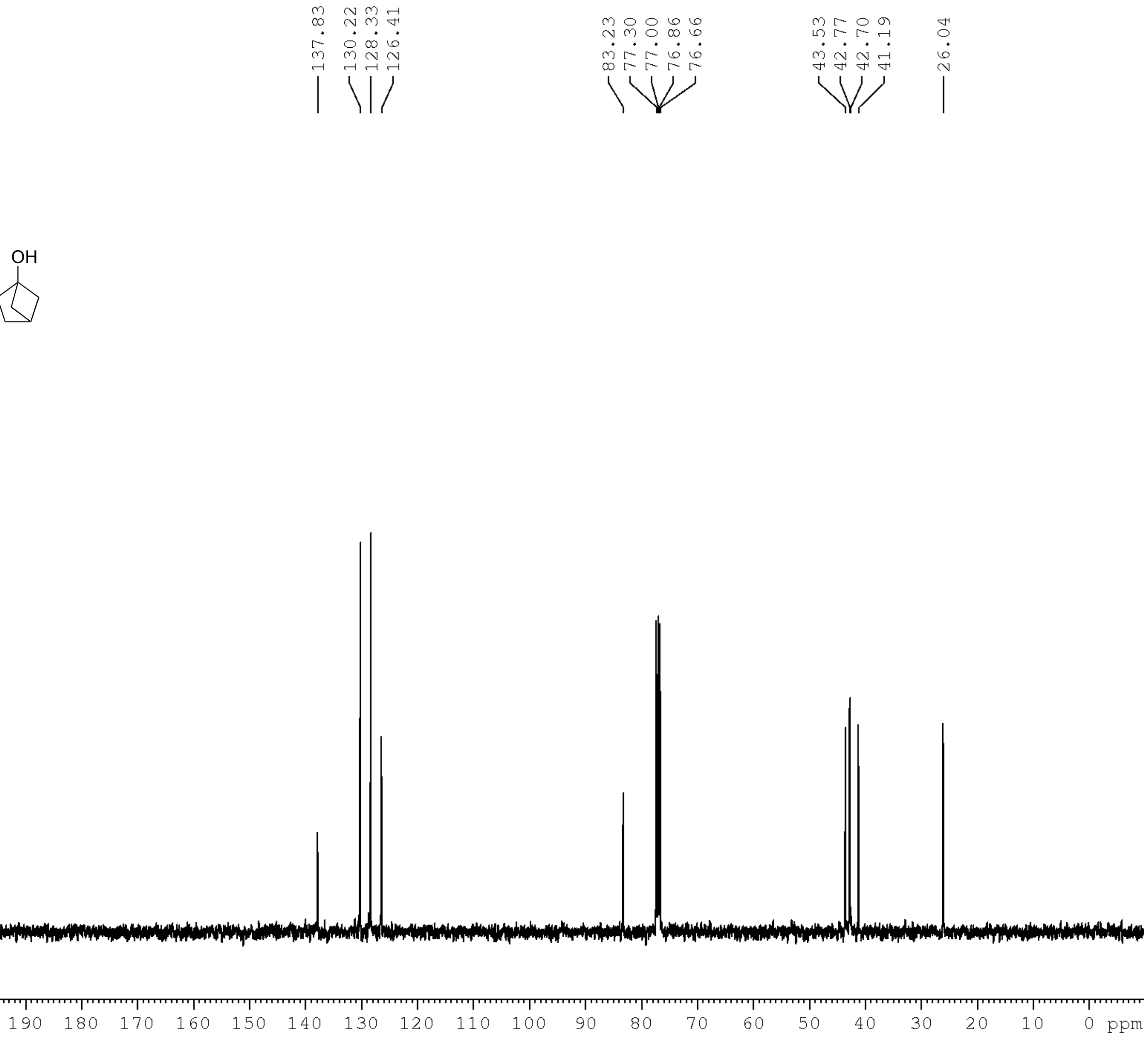


```
Current Data Parameters
NAME     20241014_deacyl
EXPNO                  2
PROCNO                 1

F2 - Acquisition Paramet
Date_           20241014
Time               20.30
INSTRUM            spect
PROBHD   Z108618_0411 (
PULPROG          zgpg30
TD                 65536
SOLVENT            CDCl3
NS                    19
DS                     4
SWH            28409.092
FIDRES          0.866977
AQ             1.1534336
RG                212.49
DW                17.600
DE                  6.50
TE                 298.1
D1            2.00000000
D11           0.03000000
TD0                    1
SFO1         100.6258487
NUC1                 13C
P1                 10.50
PLW1         42.50000000
SFO2         400.1316005
NUC2                  1H
CPDPRG[2         waltz16
PCPD2              90.00
PLW2          9.89999962
PLW12         0.29363999
PLW13         0.14747000

F2 - Processing paramete
SI                 32768
SF           100.6127792
WDW                   EM
SSB                    0
LB                  3.00
GB                     0
PC                  1.40
```

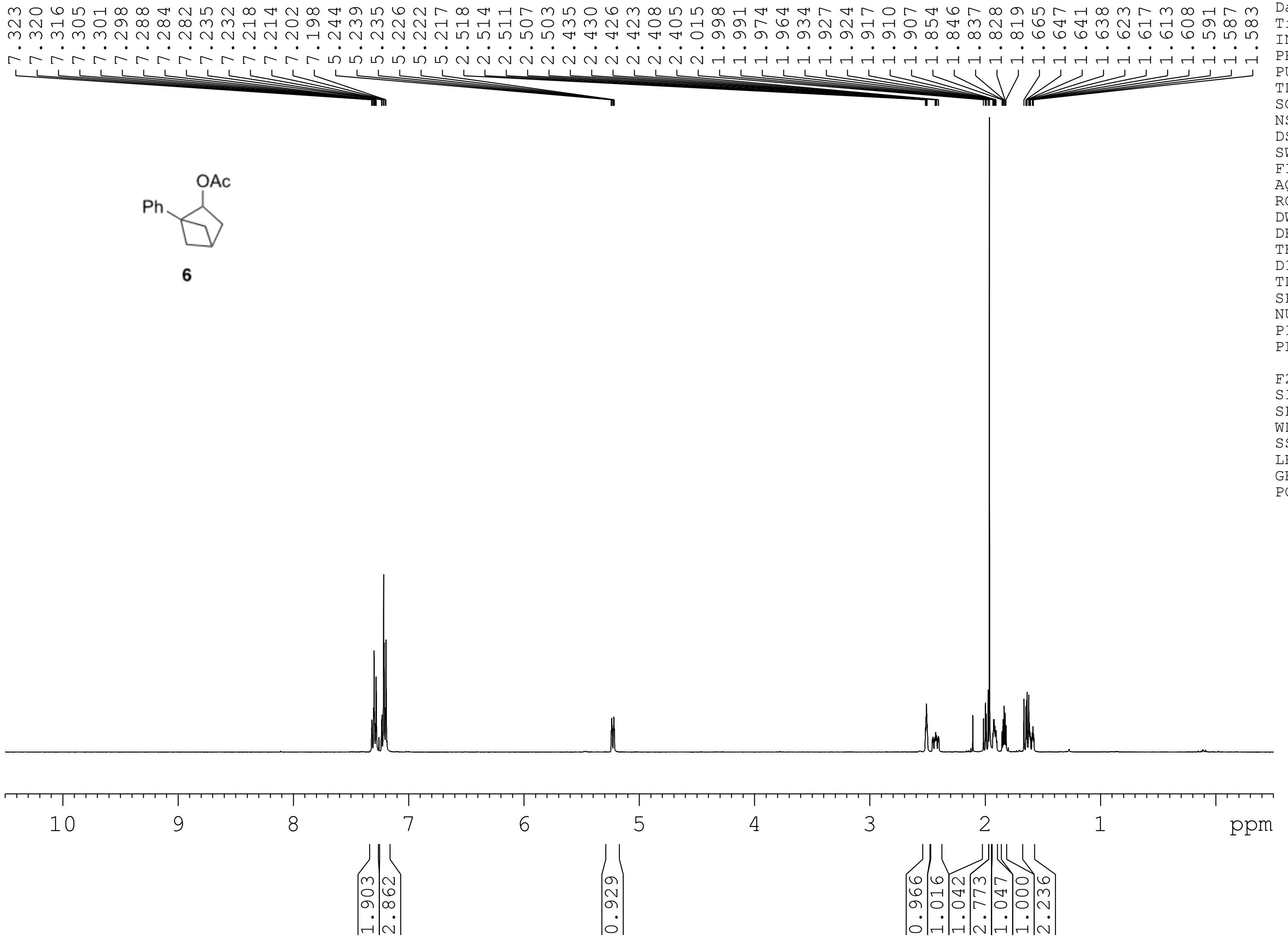


Current Data Parameters
NAME Ph to OAc
EXPNO 1
PROCNO 1

F2 - Acquisition Parameters
Date_ 20240509
Time 16.17 h
INSTRUM spect
PROBHD Z108618_0411 (
PULPROG zg30
TD 32768
SOLVENT CDCl3
NS 16
DS 0
SWH 8802.817 Hz
FIDRES 0.537281 Hz
AQ 1.8612224 sec
RG 71.05
DW 56.800 usec
DE 14.47 usec
TE 298.0 K
D1 1.00000000 sec
TD0 1
SFO1 400.1328009 MHz
NUC1 1H
P1 15.50 usec
PLW1 9.89999962 W

F2 - Processing parameters
SI 131072
SF 400.1300098 MHz
WDW EM
SSB 0
LB 0 Hz
GB 0
PC 1.00

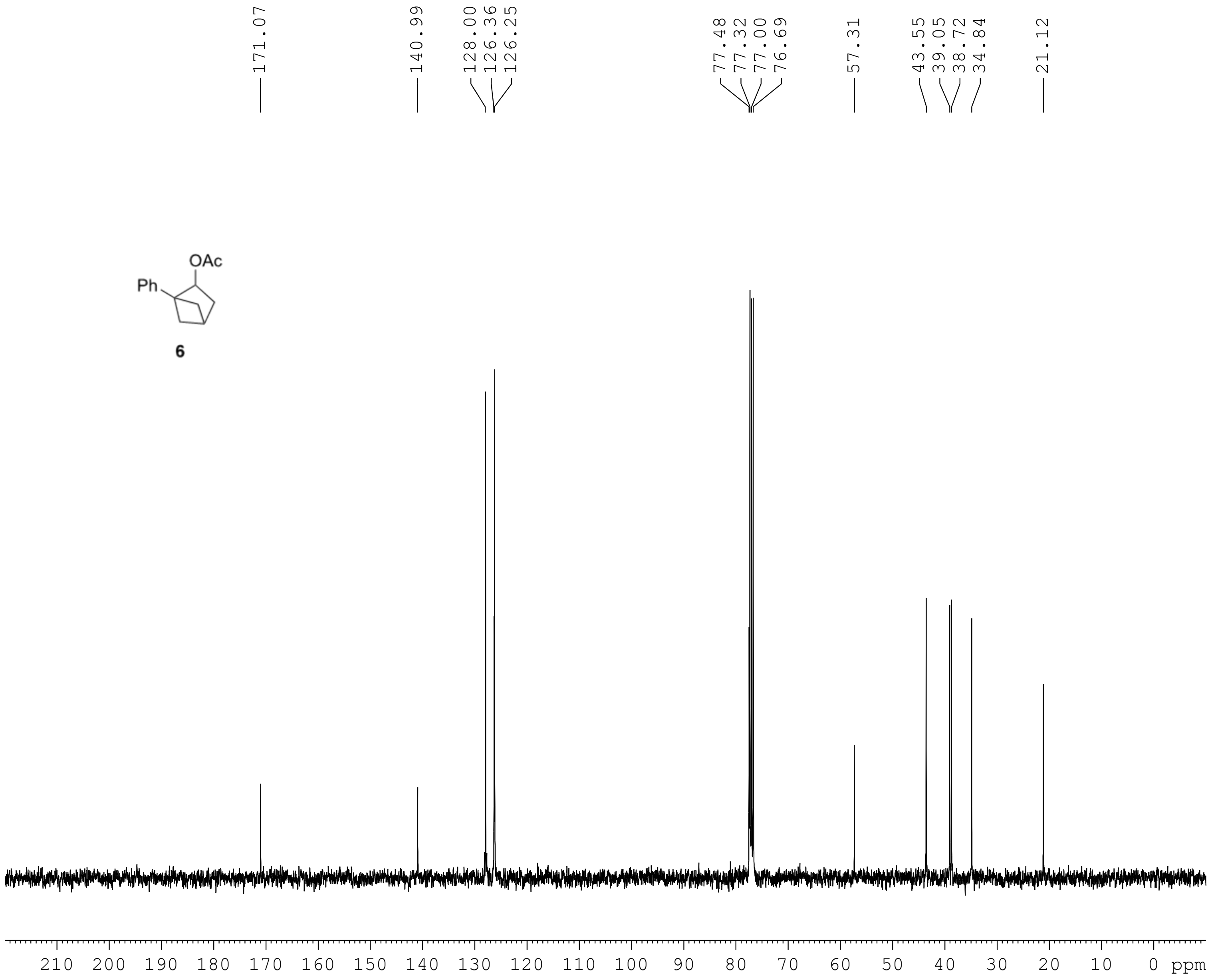


```
Current Data Parameters
NAME             Ph to OAc
EXPNO                    2
PROCNO                   1

F2 - Acquisition Parameters
Date_             20240509
Time                 16.20 h
INSTRUM              spect
PROBHD   Z108618_0411 (
PULPROG            zgpg30
TD                   65536
SOLVENT              CDCl3
NS                      40
DS                       4
SWH              28409.092 Hz
FIDRES            0.866977 Hz
AQ               1.1534336 sec
RG                  212.49
DW                  17.600 usec
DE                    6.50 usec
TE                   298.1 K
D1              2.00000000 sec
D11             0.03000000 sec
TD0                      1
SFO1        100.6258487 MHz
NUC1                   13C
P1                   10.50 usec
PLW1           42.50000000 W
SFO2        400.1316005 MHz
NUC2                    1H
CPDPRG[2           waltz16
PCPD2                90.00 usec
PLW2            9.89999962 W
PLW12           0.29363999 W
PLW13           0.14747000 W

F2 - Processing parameters
SI                   32768
SF          100.6127740 MHz
WDW                     EM
SSB                      0
LB                    3.00 Hz
GB                       0
PC                    1.40
```

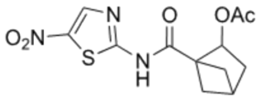

**7**

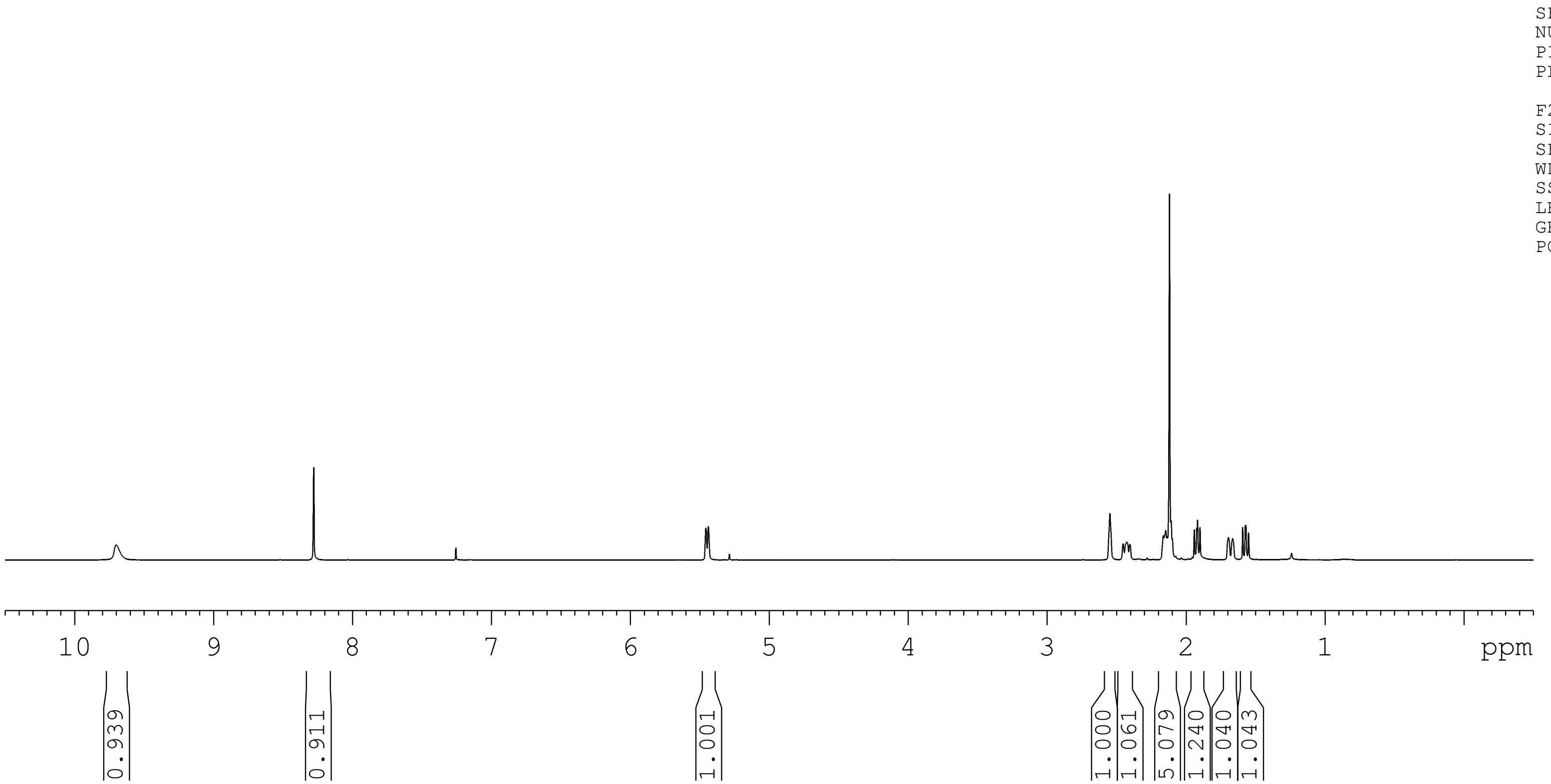


Current Data Parameters
NAME Drug H
EXPNO 1
PROCNO 1

F2 - Acquisition Parameters
Date_ 20240516
Time 17.09 h
INSTRUM spect
PROBHD Z108618_0411 (
PULPROG zg30
TD 32768
SOLVENT CDCl3
NS 20
DS 0
SWH 8802.817 Hz
FIDRES 0.537281 Hz
AQ 1.8612224 sec
RG 133.5
DW 56.800 usec
DE 14.47 usec
TE 298.0 K
D1 1.00000000 sec
TD0 1
SFO1 400.1328009 MHz
NUC1 1H
P1 15.50 usec
PLW1 9.89999962 W

F2 - Processing parameters
SI 131072
SF 400.1300107 MHz
WDW EM
SSB 0
LB 0 Hz
GB 0
PC 1.00

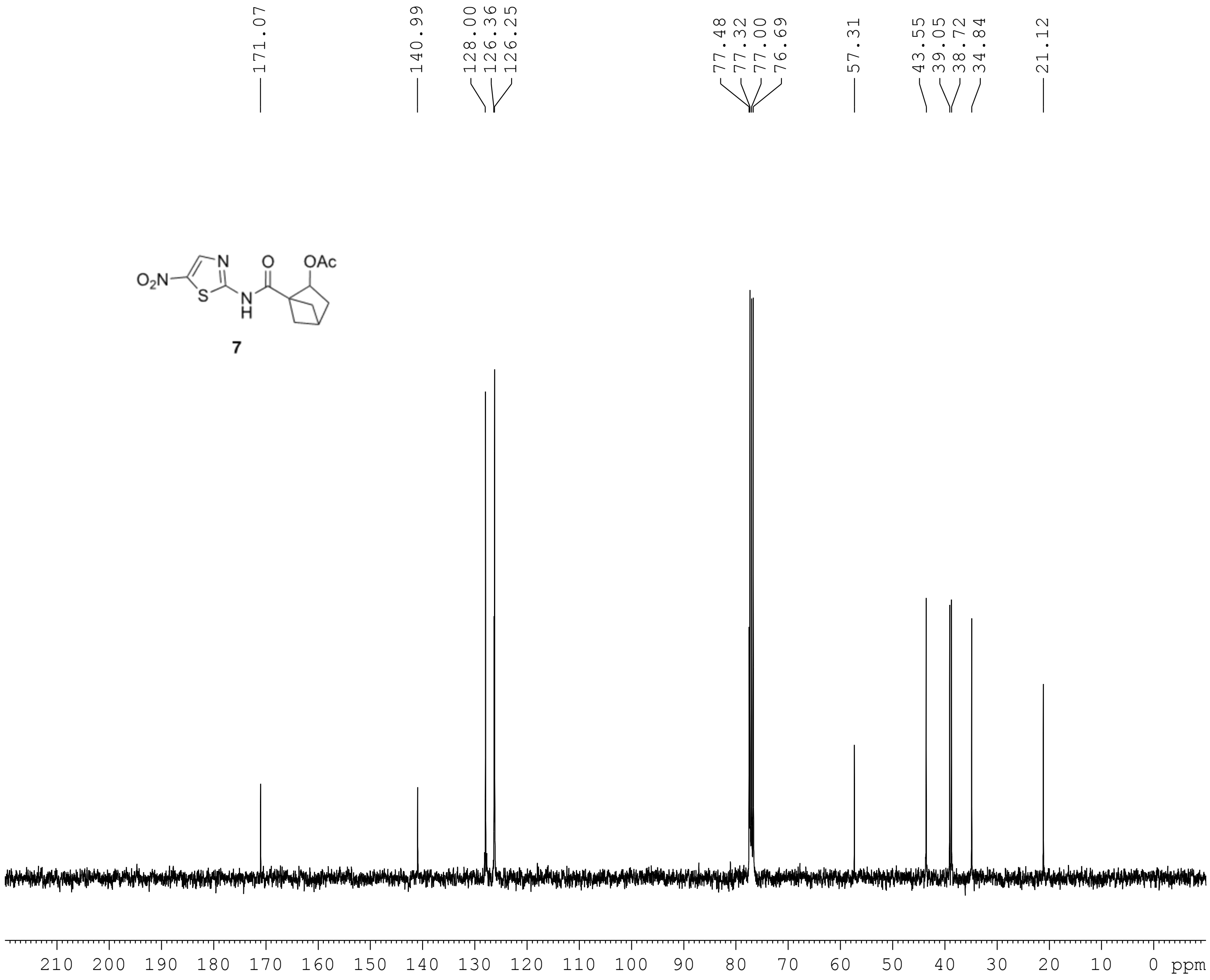


Current Data Parameters
NAME Ph to OAc
EXPNO 2
PROCNO 1

F2 - Acquisition Parameters
Date_ 20240509
Time 16.20 h
INSTRUM spect
PROBHD Z108618_0411 (
PULPROG zgpg30
TD 65536
SOLVENT CDCl3
NS 40
DS 4
SWH 28409.092 Hz
FIDRES 0.866977 Hz
AQ 1.1534336 sec
RG 212.49
DW 17.600 usec
DE 6.50 usec
TE 298.1 K
D1 2.00000000 sec
D11 0.03000000 sec
TD0 1
SFO1 100.6258487 MHz
NUC1 13C
P1 10.50 usec
PLW1 42.50000000 W
SFO2 400.1316005 MHz
NUC2 1H
CPDPRG[2 waltz16
PCPD2 90.00 usec
PLW2 9.89999962 W
PLW12 0.29363999 W
PLW13 0.14747000 W

F2 - Processing parameters
SI 32768
SF 100.6127740 MHz
WDW EM
SSB 0
LB 3.00 Hz
GB 0
PC 1.40
